# The role of the minor colonization factor CS14 in adherence to intestinal cell models by geographically diverse ETEC isolates

**DOI:** 10.1128/msphere.00302-23

**Published:** 2023-10-03

**Authors:** Emily M. Smith, Antonia Papadimas, Caitlin Gabor, Ceanna Cooney, Tao Wu, David Rasko, Eileen M. Barry

**Affiliations:** 1 Center for Vaccine Development and Global Health, University of Maryland School of Medicine, Baltimore, Maryland, USA; 2 Institute for Genome Sciences, University of Maryland School of Medicine, Baltimore, Maryland, USA; 3 Department of Microbiology and Immunology, University of Maryland School of Medicine, Baltimore, Maryland, USA; University of Michigan-Ann Arbor, Ann Arbor, Michigan, USA

**Keywords:** ETEC, colonization factor, adherence, enteroid, vaccines, fimbriae, CS14

## Abstract

Enterotoxigenic *Escherichia coli* (ETEC) is a primary causative agent of diarrhea in travelers and young children in low- to middle-income countries. ETEC adheres to small intestinal epithelia via colonization factors (CFs) and secretes heat-stable toxin and/or heat-labile toxin, causing dysregulated ion transport and water secretion. There are over 30 CFs identified, including major CFs associated with moderate-to-severe diarrhea (MSD) and minor CFs for which a role in pathogenesis is less clear. The Global Enteric Multicenter Study identified CS14, a class 5a fimbriae, as the only minor CF significantly associated with MSD and was recommended for inclusion in ETEC vaccines. Despite detection of CS14 in ETEC isolates, the sequence conservation of the CS14 operon, its role in adherence, and functional cross-reactivity to other class 5a fimbriae like CFA/I and CS4 are not understood. Sequence analysis determined that the CS14 operon is >99.9% identical among seven geographically diverse isolates with expanded sequence analysis demonstrating SNPs exclusively in the gene encoding the tip adhesin CsuD. Western blots and electron microscopy demonstrated that CS14 expression required the growth of isolates on CFA agar with the iron chelator deferoxamine mesylate. CS14 expression resulted in significantly increased adherence to cultured intestinal cells and human enteroids. Anti-CS14 antibodies and anti-CS4 antibodies, but not anti-CFA/I antibodies, inhibited the adherence of a subset of ETEC isolates, demonstrating CS14-specific inhibition with partial cross-reactivity within the class 5a fimbrial family. These data provide support for CS14 as an important fimbrial CF and its consideration as a vaccine antigen in future strategies.

**IMPORTANCE:**

Enterotoxigenic *Escherichia coli* (ETEC) infection causes profuse watery diarrhea in adults and children in low- to middle-income countries and is a leading cause of traveler's diarrhea. Despite increased use of rehydration therapies, young children especially can suffer long-term effects including gastrointestinal dysfunction as well as stunting and malnutrition. As there is no licensed vaccine for ETEC, there remains a need to identify and understand specific antigens for inclusion in vaccine strategies. This study investigated one adhesin named CS14. This adhesin is expressed on the bacterial surface of ETEC isolates and was recently recognized for its significant association with diarrheal disease. We demonstrated that CS14 plays a role in bacterial adhesion to human target cells, a critical first step in the disease process, and that adherence could be blocked by CS14-specific antibodies. This work will significantly impact the ETEC field by supporting inclusion of CS14 as an antigen for ETEC vaccines.

## INTRODUCTION

Enterotoxigenic *Escherichia coli* (ETEC) is a primary causative agent of diarrheal disease in children under 5 years of age in low- to middle-income countries (LMICs) and in travelers from industrialized countries, where it causes ~84 million diarrheal episodes and 44,000 estimated deaths per year in children younger than five (1 – 5). ETEC adheres to the small intestinal epithelium using surface-expressed colonization factors (CFs) that are fimbrial, fibrillar, or non-fimbrial structures. This host-pathogen interaction is critical for pathogenesis, as the subsequent secretion of heat-stable toxin (ST) and/or heat-labile toxin (LT) leads to the disruption of cyclic nucleotide production and ion transporter expression and function, resulting in profuse watery diarrhea (6 – 9). In addition to CFs and toxins, ETEC expresses other virulence factors, including EatA and EtpA, which contribute to pathogenesis and disease (10 – 14).

Over 30 types of antigenically distinct CFs have been identified in ETEC. Major CFs include CFA/I and coli surface (CS) antigens CS1-CS6 and are most prevalently associated with isolates causing moderate-to-severe diarrhea (MSD) (15). The role of minor CFs, including CS14, in pathogenesis is less understood. CS14 was first identified as the putative colonization factor O166 (PCFO166) on geographically diverse ST-only clinical ETEC isolates belonging to the prevalent O166 serogroup (16). CS14 was subsequently found to be a common CF in multiple epidemiological studies involving children under five (17 – 29), military personnel (30), and adults in LMICs (24, 31, 32). From these studies, a combined average of 10.4% of all ETEC strains isolated from individuals with diarrhea contained the genes encoding CS14 (15, 17, 19 – 22, 24 – 32). Limited studies investigating CS14 expression in a subset of ETEC strains used CFA or SP1 media with bile salts (33). Among ST-only and LT/ST-ETEC strains expressing a single CF, the Global Enteric Multicenter Study (GEMS) identified CS14 as the only minor CF with a statistically significant association with MSD; thus, CS14 has been recommended for consideration for inclusion in future ETEC vaccine development and prophylactic strategies (15).

**Fig 1:**
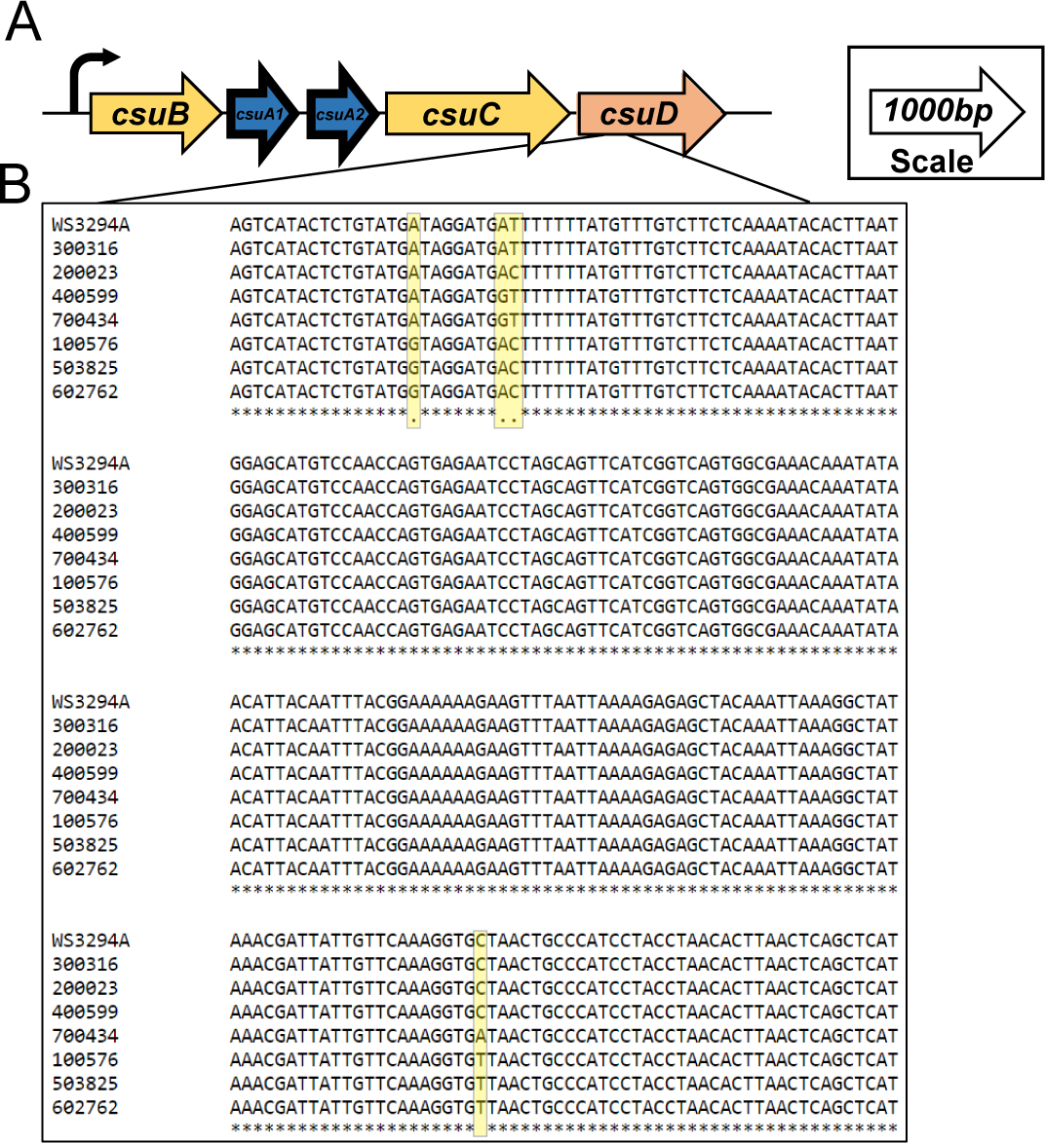
Sequence analysis of the CS14 operon in GEMS clinical ETEC isolates. (**A**) The CS14 operon is depicted with arrows indicating coding sequences and lines representing intergenic regions. The size of the arrows represents relative size (bp) of genes. The bolded subunits (blue) represent the major subunit of the operon. Curved arrows indicate a putative promoter. (**B**) The CS14 operon in seven ETEC isolates was sequenced using Sanger sequencing, and sequences were compared to the CS14 reference strain WS3294A (GenBank AY283611.1) to determine sequence identity using clustal format alignment by MAFFT. Nucleotide differences in the *csuD* gene are highlighted in yellow.

CS14 is encoded by a five-gene operon that uniquely encodes two nearly identical major subunits and is assembled by the chaperone-usher pathway (Fig. 1) (34). The genes were originally identified on a 98-kDa plasmid that also contained genes encoding ST (16). CS14 is classified as a class 5 fimbriae based on homology with other CFs in this family, including CFA/I, CS1, CS2, CS4, CS17, and CS19; CS14 has the highest homology to those in the class 5a family, including CFA/I (90.73% operon nucleotide identity) and CS4 (93.1% operon nucleotide identity) (34 – 38). One study comparing nucleotide and amino acid homology reported this using a reference CS14 ETEC strain WS3294A; however, additional studies are needed to understand the sequence conservation of the CS14 operon across global ETEC isolates (35).

The high level of homology among heterologous CFs in the class 5a fimbrial family led to investigation of the potential cross-reactivity of antibodies induced against a single CF. Cross-reactivity of IgG and IgA antibodies against CFA/I and CS14 has been observed *in vitro* using antibodies and antibody-secreting cells isolated from naturally infected individuals as well as vaccinated adults in clinical trials (23, 39 – 41). However, no functional assays were included. A series of other studies assessed polyclonal and monoclonal antibodies generated against purified fimbriae or specific fimbrial subunits for cross-reactivity. Anti-CfaE polyclonal and monoclonal antibodies were shown to cross react with CS14 and/or CS4 using enzyme-linked immunosorbent assay (ELISA) (35, 39, 42, 43). Anti-CfaE polyclonal antibodies, but not monoclonal antibodies, were able to inhibit CS14+ ETEC hemagglutination of bovine and human erythrocytes (35, 39, 42). However, these anti-CfaE polyclonal antibodies were not able to inhibit binding of CS14+ ETEC to Caco-2 cells (35, 42). The potential generation of functionally cross-reactive antibody responses against CFs has significant implications for ETEC vaccine development strategies.

There is no licensed ETEC vaccine, and most vaccine development strategies do not include antigens targeting minor CFs, such as CS14. Despite the growing epidemiological evidence for the importance of CS14 in ETEC-mediated diarrheal disease, there are limited studies that investigate its role in adherence to intestinal cells and the possibility of functional cross-reactive immune responses among CS14, CFA/I, and CS4 (44 – 47). The current study presents data on the sequence conservation of CS14 across geographical isolates, a novel iron-limiting growth condition for optimal CS14 expression, CS14-mediated adherence, and the ability of homologous anti-CS14 or heterologous anti-CFA/I or anti-CS4 antibodies to inhibit adherence using HT-29 human intestinal cell lines and the human enteroid model.

Enteroids are derived from Lgr5+ stem cells, isolated from small intestinal crypts of healthy human donors, which can be differentiated to include multiple intestinal cell types, including goblet, Paneth, enteroendocrine, and epithelial cells (48, 49). With these specialized cell types, enteroids recapitulate the major functions of human intestine and can be seeded as monolayers on transwells to allow apical infection to understand gastrointestinal physiology and pathogenic mechanisms of a variety of microbes (50 – 52). Enteroids have recently been used to demonstrate a role for CFs and other virulence factors, including CFA/I, EatA, and EtpA, in ETEC adherence as well as toxin delivery (14, 53 – 55). This model also allows the evaluation of the blocking capacity of anti-CF antibodies (55). The direct relevance to human biology, rigor of this system, and feasibility of use in tissue culture allow the human enteroid to serve as an invaluable and sensitive tool to study CS14-specific roles during ETEC pathogenesis.

## MATERIALS AND METHODS

### Strains used in the study and culture media

ETEC clinical isolates and mutant strains are described in Table 1. Seven CS14-only, ST-only ETEC clinical isolates, one from each of the seven geographical sites from the GEMS, were evaluated. ETEC strains from GEMS were grown on CFA agar (56) or CFA agar with 200 µM of deferoxamine mesylate (DFOM) for expression experiments, electron microscopy (EM), and adherence assays with HT-29 cells and enteroid monolayers. ETEC strains from GEMS were also grown on CFA agar with 0.15% bile salts (No. 3, Becton Dickenson) for a supplemental adherence assay with HT-29 cells. The wild-type ETEC strains H10407 and DS9-1 were grown on CFA agar (57). TOP10 *E. coli* (pBAD-CS14) was grown on Luria-Bertani (LB) agar with carbenicillin (100 µg/mL) and 0.2% arabinose to induce CS14 expression or LB agar with carbenicillin (100 µg/mL) for no CS14 expression. An additional 84 CS14+ ETEC strains from the GEMS were studied exclusively for *csuD* sequence analysis (15, 58).

**TABLE 1:** Bacterial strains used in this study

Strain/plasmid	Description (country of origin)	Reference
100576	ST, CS14 (The Gambia)	(15)
200023	ST, CS14 (Mali)	(15)
300316	ST, CS14 (Mozambique)	(15)
400599	ST, CS14 (Kenya)	(15)
503825	ST, CS14 (India)	(15)
602762	ST, CS14 (Bangladesh)	(15)
700434	ST, CS14 (Pakistan)	(15)
*E. coli*(pBAD-CS14)	pBAD/myc-HisC::CS14, high level of inducible CS14 expression	This study
WS3294A	CS14 reference strain	(35)
H10407	LT, ST, CFA/I (Bangladesh)	(59)
DS9-1	ST, CS4, CS6 (Saudi Arabia)	(57)

### Construction of strain *E. coli* (pBAD-CS14)

*E. coli* (pBAD-CS14) was engineered for this study, and primers used are shown in Table 2. The CS14 operon was amplified by PCR using primers E04_F and E06_R from ETEC clinical isolate 200023; the PCR product was digested by NcoI and SalI. The digested CS14 fragment was ligated into similarly digested pBAD/myc-HisC (NEB) using NEB T4 DNA ligase and transformed into TOP10 competent cells (Invitrogen). Positive transformants were confirmed by PCR using primers E06_F and E07_R, digestion, and sequencing. Western blot confirmed CS14 expression under pBAD promoter induction with arabinose.

**TABLE 2:** Primers used in this study

Primer	Description	Sequence (5′→3′)
E01-F	CS14 operon_first half, sequencing	CGGCGGTTATGGAAGAGGTGG
E01-R	CS14 operon_first half, sequencing	CGAAAACGTCCCATTGCATATCACC
E02-F	CS14 operon_second half, sequencing	GACACAGCCGCGCCTGTTCCT
E02-R	CS14 operon_second half, sequencing	TCTAGTAGAGATTTATTGCGATGC
E03-F	Promoter – *csuB*, sequencing	GGCTACCGTCTGACAGCTAT
E04-F	*csuB,* strain construction	TAACCATGGAGAAATTATTTTATTTACTAAGTTTAC
E05-F	*csuB – csuA1*, sequencing	CCCGGTACAACTGAAGAAC
E06-F	*csuB – csuA1 – csuA2*, strain construction, endpoint PCR	ACGGGAATGTTAGAGCAGGTGTTAAAG
E03-R	*csuA2 – csuC*, sequencing	CCCCATAAATCACGGAAAGAGTCGGG
E07-F	*csuC*, sequencing	CTGGATTCTGAAGCACACAG
E08-F	*csuC*, sequencing	ACTTCCTTTACCCGATATTGATGG
E09-F	*csuC*, sequencing	GGTTGGAGTGGATGCTACG
E10-F	*csuC – csuD*, sequencing	GCAGAAGGTAGACTTTCAGGTC
E11-F	*csuD*, sequencing	TGTTAATAGCACACGTTCCTTACCAG
E04-R	*csuD*, sequencing	GTTAACAAACAGATTACCCC
E05-R	*csuD*, sequencing	TAATACGTAGAGTGTTTGACTACTTGGTGTG
E06-R	strain construction	TAAGTCGACCTAGAGTGTTTGACTACTTGGTG
E07-R	strain construction	TCATTAAGGAATGAGCAGCTACGAATG
E12-F	*sth,* endpoint PCR	GTATTGTCTTTTTCACCTTTC
E12-R	*sth,* endpoint PCR	GCACCCGGTACAAGCAGGA
E13-F	*etpA,* endpoint PCR	CAGACAGCTACACCAAC
E13-R	*etpA*, endpoint PCR	CGATTGAGTCGTCTCAG
E14-R	*csuA1/csuA2*, endpoint PCR	GAGTCGGGTATATCATCTGCA

### CF-specific antibodies

CS14 fimbriae were purified from the strain *E. coli* (pBAD-CS14) that contains the CS14 operon from the strain 200023 for expression. CFA/I fimbriae were purified from the strain *E. coli* (pGA2-CFA/I) that contains the CFA/I operon from the strain H10407 for expression (60). CS4 fimbriae were purified from the strain *E. coli* (pGA2-CS4) that contains the CS4 operon from the strain E11881A for expression (61). CS14, CFA/I, and CS4 fimbriae were purified at the Center for Vaccine Development and Global Health (62). Anti-CS14, anti-CFA/I, and anti-CS4 polyclonal antibodies were generated as a fee-for-service contract with Rockland Immunochemicals using the purified fimbriae. Rabbits were immunized with purified fimbriae, and total polyclonal sera were collected. The sera were absorbed to reduce non-specific antibodies with whole cell lysate of *E. coli* DH5α. Non-specific sera were also collected from these rabbits prior to immunization.

### Sanger sequencing of CS14 operon and analysis

The CS14 operon from the GEMS strains 100576, 200023, 300316, 400599, 503826, 602782, and 700434 was amplified in two parts using E01-F and E01-R for the first half (3458 bp) and E02-F and E02-R for the second half (3906 bp). The PCR products were purified for sequencing using gel extraction. Primers were used to sequence the CS14 operon using Sanger sequencing (Genewiz) (Table 2). Alignment of the operon sequences from all seven GEMS strains with the reference strain WS3294A (GenBank accession AY283611) was performed using clustal format alignment by MAFFT (Fig. S1).

The amino acid sequence of CsuD from the seven GEMS strains was also determined using the Open Reading Frame Finder (ORFfinder) tool from NCBI (RRID:SCR_016643), and predicted protein sequences were aligned using clustal format alignment by MAFFT v. 2.1 (Fig. S2).

### Bioinformatics analyses

Differences in gene content, specifically genes encoding ETEC colonization factors, regulators, and virulence factors, between ETEC WS3294A and the seven ETEC CS14+ strains from the GEMS were identified using BLASTN large-scale BLAST score ratio (LS-BSR) analysis as previously described (58). LS-BSR analysis was performed using the whole genome sequences of the seven CS14+ ETEC strains, obtained using Illumina sequencing methods. The protein-coding genes of each genome were assigned to gene clusters with >90% nucleotide identity and >90% alignment length using CD-HIT v. 4.6.7. Gene clusters identified with a BSR value of >0.8 were considered to represent significant similarity, while gene clusters with a BSR value of <0.4 were considered absent. Specific genes were confirmed to be present or absent using endpoint PCR with primers described in Table 2.

Specific *csuD* sequence analysis was also performed using an additional 84 CS14+ ETEC strains from the GEMS (15). These strains were included based on sequence availability (58), and all geographical sites from the GEMS were represented. Sequences of *csuD* from these 84 ETEC strains were obtained using the whole genome sequencing method Illumina. The gene sequence from the seven GEMS strains and the additional 84 ETEC strains (for a total of 91 strains) was aligned using gapped BLASTn and PSI-BLAST (63), compared to the *csuD* gene sequence from the reference strain WS3294A.

### Expression of colonization factors

Whole cell bacterial lysates were prepared for Western blot analysis as follows. Bacteria were harvested from overnight growth on CFA agar with or without 200 µM DFOM in PBS, and the OD_600_ was used to normalize to the samples for bacterial numbers (55). The normalized suspensions were then mixed 1:1 with 2× Laemmli Sample Buffer (Bio-Rad). Proteins were separated on a 12% Mini-Protean TGX Precast Gel (Bio-Rad) and transferred to PVDF membrane. The membrane was blocked in a 10% (wt/vol) nonfat milk buffer in PBS and incubated with absorbed polyclonal Rabbit α-CS14 (Rockland Immunochemicals). The secondary antibody was Goat α-Rabbit 680 nm (Invitrogen), and proteins were visualized using the LI-COR Odyssey Laser Scanner. Additional Western blots of CFA agar with or without 200 µM DFOM grown strains were performed and stained with Rabbit anti-DnaK (Invitrogen) and Goat α-Rabbit 680 nm (Invitrogen) antibodies to confirm equal loading.

### Electron microscopy

Transmission electron microscopy (TEM) was used for visualization of colonization factors. Bacteria grown on CFA agar with or without 200 µM DFOM were resuspended in buffer (1% bovine serum albumin [BSA] and 1% Tween 20 in PBS) and stained with 0.5% uranyl acetate (Electron Microscopy Sciences) on Formvar carbon-coated copper grids 400 mesh (Electron Microscopy Sciences) before examination using Tecnai T12 TEM. Immunogold labeling of CS14 was performed using Formvar carbon-coated nickel grids 400 mesh (Electron Microscopy Sciences). The representative CS14-expressing ETEC strain 200023 was incubated in rabbit anti-CS14 primary antibodies (Rockland Immunochemicals) for 1 h. The secondary antibody consisted of 10-nm gold particles conjugated to Goat α-Rabbit antibody (Sigma). The 0.5% uranyl acetate-negative stain was added to grids for 1 min before visualization using TEM.

### HT-29 cell culture and adherence assays

Human HT-29 (ATCC HTB-38) monolayers were cultured in Dulbecco’s Modified Eagle Medium (DMEM) supplemented with 10% Fetalplex and 2% HEPES in 150-cm^2^ flasks. The cells were incubated in 5% CO2 at 37°C and passaged once a week. For the adherence assays, cells were seeded at a density of 7 × 10^5^ cells per well in a 24-well plate and incubated overnight. Bacterial inocula were prepared in DMEM by resuspending a loopful of bacteria from CFA agar with or without 200 µM DFOM. The bacterial suspension was diluted to the desired bacterial concentration in DMEM (2.8  ×  10^8^ cells/mL). Monolayers were washed with DMEM and infected with 250-µL bacteria for a final concentration of 7  ×  10^7^ CFU (multiplicity of infection [MOI] = 100) on HT-29 monolayers. For experiments with CF-specific antibodies, 810 µL of bacterial strains and 90 µL of undiluted CS14-specific, CFA/I-specific, or CS4-specific antibodies (Rockland Immunochemicals) were incubated for 1 h at 37°C on a rotator for a final 1:10 dilution of antibody to bacteria. These studies were performed based on the ETEC inhibition assays as performed previously (64). The infected monolayers were incubated at 37°C with 5% CO_2_ for 2 h to allow bacterial adherence to cell monolayers. To isolate adherent bacteria, remaining monolayers were washed with PBS three times on the plate shaker at 550 rpm and lysed using 1% Triton X-100 by gentle scraping and incubation at room temperature for 20 min. Serial dilutions were plated in quadruplicate on LB agar and incubated overnight at 37°C. The percentage of CFU recovery was determined as follows: adherent bacteria/bacterial inoculum × 100%. For CF-antibody adherence inhibition studies, the percentage of bacterial binding inhibition was calculated using the average of wild-type bacteria enumerated as 100% binding as follows: 100% − [(number of bacteria incubated with CF-specific antibody/number of wild-type bacteria) × 100%] (64). Antibody inhibition experiments were also similarly performed with *E. coli* (pBAD-CS14). This control strain was incubated with either CS14-specific antibodies or non-specific rabbit preimmune sera prior to infection.

### Intestinal enteroid culture and adherence assays

Human enteroid cultures from ileum were established from biopsy specimens obtained after endoscopic or surgical procedures by utilizing methods developed by the laboratory of Hans Clevers (49). One enteroid line was used in this work (46I). This line is secretor positive, as determined by immunostaining with a UEA-1 conjugate, and is type O blood group (55). Enteroids isolated from intestinal crypt cells were cultured as 3D cysts embedded in Matrigel (Corning) and passaged approximately every 7  days. All enteroid media were prepared as reported previously (65). 3D enteroids were harvested by incubation in an organoid harvesting solution (Cultrex) followed by vigorous shaking and trituration. To form enteroid monolayers, the triturated enteroids were resuspended and seeded on polycarbonate membrane 24-well cell culture inserts with a 0.4-µm pore size (transwell filters; Corning) that were precoated with human collagen IV solution (Sigma). Monolayers were incubated in enteroid propagation media with Y-27632 and CHIR99021 (65, 66). Typically, confluence in these enteroid cultures was achieved in 10 to 14 days as assessed by the increase in transepithelial electrical resistance measured using an epithelial volt/ohmmeter (EVOM^2^; World Precision Instruments). Confluent monolayers were differentiated by incubation with Wnt3A-free and R-spondin-1-free medium for 5 days. Bacterial adherence assays were performed at 5 days post differentiation.

Adherence assays were performed using differentiated enteroid monolayers. Bacterial inocula were prepared in DMEM by resuspending a loopful of bacteria from CFA agar with or without 200 µM DFOM. The bacterial suspension was diluted to the desired bacterial concentration (1  ×  10^7^ CFU/mL) in DMEM, and monolayers were washed with DMEM. The 100  µL of the inoculum (∼1  ×  10^6^ CFU) and 100 µL of DMEM were added to enteroid monolayers. For experiments with CF-specific antibodies, 810 µL of bacterial strains and 90 µL of undiluted CS14-specific, CFA/I-specific, or CS4-specific antibodies (Rockland Immunochemicals) were incubated for 1 h at 37°C on a rotator for a final 1:10 dilution of antibody to bacteria. The infected monolayers were incubated at 37°C with 5% CO_2_ for 4 h to allow bacterial adherence to enteroid monolayers. To isolate adherent bacteria, remaining monolayers were washed with PBS three times on the plate shaker at 550 rpm and lysed using 1% Triton X-100 by gentle scraping and incubation at room temperature for 20 min. Serial dilutions were plated in quadruplicate on LB agar and incubated overnight at 37°C. The percentage of CFU recovery was determined as follows: adherent bacteria/bacterial inoculum × 100%. For CF-antibody adherence inhibition studies, the percentage of bacterial binding inhibition was calculated using the average of wild-type bacteria enumerated as 100% binding as follows: 100% − [(number of bacteria incubated with CF-specific antibody/number of wild-type bacteria) × 100%] (64).

### Statistical analyses

Statistical significance was determined using two-tailed, unpaired Student’s *t* test. For multiple comparisons, analysis of variance (ANOVA) was used with a Sidak (HT29 adherence assays) or Bonferroni (enteroid adherence assays) posttest to determine statistical differences within specific groups, as noted in figure legends. Each dot in the figures represents data collected from an individual monolayer. Replicates from multiple independent experiments were pooled. The number of independent experiments pooled for each figure is reported in the figure legends. GraphPad Prism software was used for all statistical analyses.

## RESULTS

### CS14 sequence conservation across clinical ETEC isolates from seven geographical areas

The sequence conservation of the CS14 operon across global ETEC isolates has not been investigated. Given the impact of sequence variability on antigen design for vaccine development, we sequenced the CS14 operons from seven geographically diverse CS14+ ETEC strains from the GEMS (Tables 1 and 2) and performed an analysis using the clustal alignment tool MAFFT (Fig. 1; Fig. S1). The ~5.5-kb operons from all seven strains and the reference strain WS3294A were >99.93% identical to each other (Table 3). The genes encoding the chaperone CsuB, usher CsuC, and repeating major subunits CsuA1/CsuA2 were 100% identical.

**TABLE 3:** Percent nucleotide identity of the CS14 operon (*csuB – csuD*) of GEMS clinical ETEC isolates determined by multiple sequence alignment using clustal format alignment tool MAFFT^*a*^

Isolate	WS3294A	200023	300316	400599	700434	100576	503825	602762
**WS3294A**	**100**	99.98	**100**	99.98	99.96	99.95	99.95	99.95
**200023**	99.98	**100**	99.98	99.96	99.95	99.96	99.96	99.96
**300316**	**100**	99.98	**100**	99.98	99.96	99.95	99.95	99.95
**400599**	99.98	99.96	99.98	**100**	99.98	*99.93*	*99.93*	*99.93*
**700434**	99.96	99.95	99.96	99.98	**100**	*99.93*	*99.93*	*99.93*
**100576**	99.95	99.96	99.95	*99.93*	*99.93*	**100**	**100**	**100**
**503825**	99.95	99.96	99.95	*99.93*	*99.93*	**100**	**100**	**100**
**602762**	99.95	99.96	99.95	*99.93*	*99.93*	**100**	**100**	**100**

^*a*^ Italicized percentages indicate the lowest level of identity. Bolded percentages indicate the highest level of identity.

Specific nucleotide polymorphisms (SNPs) in four locations or “hot spots” were observed exclusively in the gene encoding the fimbrial tip adhesin CsuD (Fig. 1B; Table S1). Five different SNP patterns were identified among the seven geographically distinct ETEC isolates encompassing these hot spots (Table 4). Pattern 1 (AATC) was observed in the reference strain and one of seven GEMS isolates. Pattern 2 (AACC), pattern 3 (AGTC), and pattern 4 (AGTA) were each identified in only one of seven isolates. Pattern 5 (GACT) was identified in three strains. These SNP patterns were not grouped according to geographic location; the four strains from Africa and three strains from Asia were not more similar to each other by sequence. Notably, *csuD* from strains 100576, 503825, and 602762 were 100% identical to each other, sharing the same mutations in *csuD* (Fig. 1B; Table S1). This was also observed between the reference strain WS3294A and the GEMS strain 300316 (Fig. 1B; Table S1). In addition, each SNP results in substitutions in the amino acid sequence of CsuD, which may affect the structure of CsuD and how it interacts with CS14-specific antibodies (Fig. S2) (35).

**TABLE 4:** SNP patterns identified in the “hot spots” of the *csuD* gene following sequence analysis of 91 ETEC strains, including the seven CS14+ GEMS clinical ETEC isolates

Pattern	Hot spot in *csuD*				ETEC strains with pattern (#)	ETEC isolates with pattern
	1	2	3	4		
Pattern 1	A	A	T	C	54	WS3294A, 300316
Pattern 2	A	A	C	C	4	200023
Pattern 3	A	G	T	C	1	400599
Pattern 4	A	G	T	A	1	700434
Pattern 5	G	A	C	T	17	100576, 503825, 602762
Pattern 6	A	A	T	T	2	
Pattern 7	G	A	C	C	2	
Pattern 8	A	A	C	T	9	
Pattern 9	A	A	G	C	1	

The analysis of *csuD* was expanded from these seven GEMS strains to include 91 total CS14+ ETEC clinical strains from the seven regions of the GEMS (15) (Fig. S3). Notably, the five SNP patterns identified in the initial analysis of seven GEMS isolates were also observed across the 91 strains, compared to the reference strain WS3294A (Table 4). Fifty-nine percent (54/91) of strains shared the pattern 1 (AATC), similar to the reference strain, while 19% (17/91) of strains shared pattern 5 (GACT). Other SNP patterns were observed at lower frequencies (Table 4). The expanded analysis of the 91 strains revealed four additional SNP patterns across the four “hot spots” in *csuD*, including pattern 6 (AATT), pattern 7 (GACC), pattern 8 (AACT), and pattern 9 (AAGC). Additional SNPs were observed in the *csuD* gene of ETEC strains but did not appear as a pattern or at the same incidence as the identified SNP patterns (Fig. S3). Given the critical role of the tip adhesin to ETEC adherence, it is important to understand the sequence variability among strains in order to determine the impact on antigen design for vaccines (39, 43, 67).

The upstream sequences (322 bp) of the CS14 operons were also analyzed and compared using the alignment tool MAFFT. The three previously described binding sites for the CF regulator Rns (68) and a putative binding site for the iron-sulfur cluster regulator IscR (69) were identified and were nearly 100% identical among the seven geographical isolates and the reference strain WS3294A with the exception of strain 602762 (Fig. S4). Strain 602762 has one additional nucleotide in the putative binding site for IscR, but this may not affect the site given that IscR binds to conserved binding motifs and not specific sequences (Fig. S4) (70). Only one other nucleotide difference was observed between the strains in the upstream region but was not located in a known regulator binding site or other transcriptional site (Fig. S4). Previous studies investigating class 5 fimbrial expression and, more specifically, CS14 expression have identified Rns and IscR as important regulators (68, 69). Given that the binding sites for these regulators are highly similar, it is probable that they regulate CF expression similarly in these ETEC strains.

Further genomic analysis was performed to query the presence of genes encoding regulators and other known ETEC virulence factors. Using a large-scale blast ratio (LSBSR) analysis that was further confirmed by endpoint PCR for a subset of the genes investigated, all seven GEMS isolates were observed to share other non-CF virulence genes, including *sth, etpBAC,* and *yghJ* (Table S2). They also shared the homologous genes of important regulators, including *rns, iscR,* and *hns* (Table S2). A subset of strains had *fimH* or *eatA*, which can both contribute to adherence by ETEC. Additional studies are required to confirm expression of these regulators and non-CF adhesins in these strains.

### CS14 expression is induced in ETEC clinical isolates following growth in iron-limiting conditions

**Fig 2:**
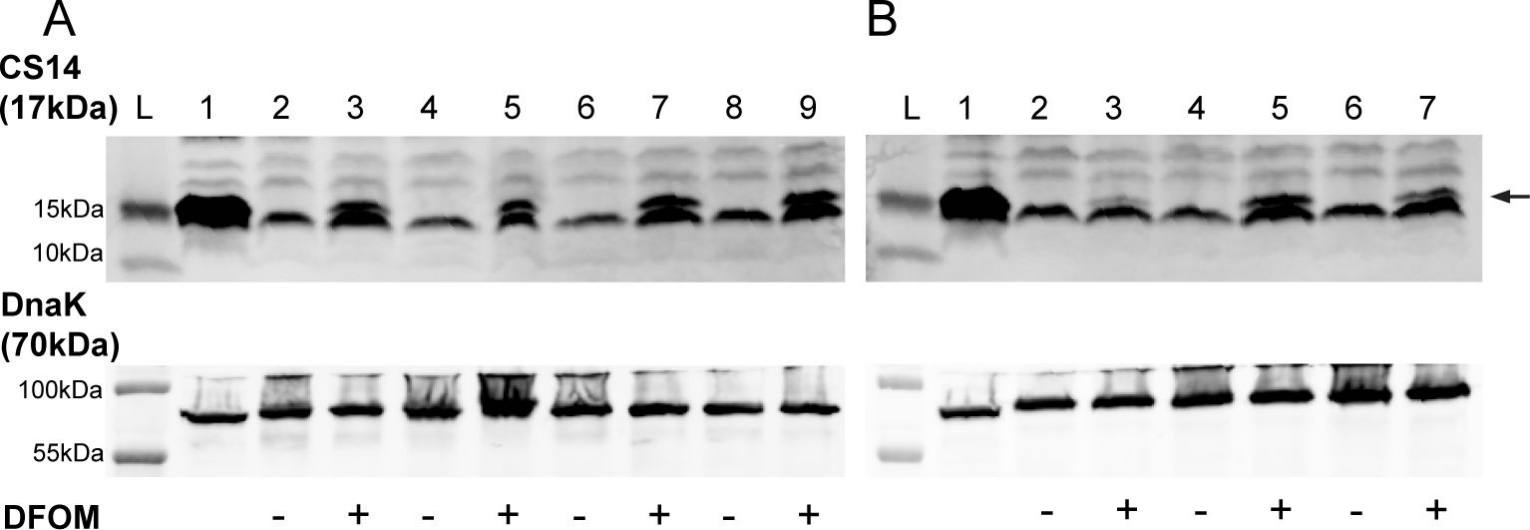
Induced expression of CS14 by GEMS clinical ETEC isolates grown in iron-limiting conditions. ETEC strains were grown on CFA agar with or without the iron chelator deferoxamine mesylate (DFOM 200 µM). Whole cell bacterial lysates were probed with anti-CS14 or anti-DnaK antibodies. (A) Ladder (**L**); lane 1, *E. coli* (pBAD-CS14); lane 2 and 3, 100576; lane 4 and 5, 200023; lane 6 and 7, 300316; lane 8 and 9, 400599. (B) Ladder (**L**); lane 1, *E. coli* (pBAD-CS14); lane 2 and 3, 503825; lane 4 and 5, 602762; lane 6 and 7, 700434. Arrow indicates band for CsuA at approximately 17 kDa. DnaK Western blots confirmed equal loading of samples with band size of 70 kDa.

Expression of individual CFs is known to be regulated by a series of complex differential CF-specific mechanisms. Previous studies have demonstrated similar expression regulation of CFA/I and CS14 by the CF regulator Rns (68, 71, 72) as well as comparable expression in differential growth media conditions, including CFA agar with bile salts or SP1 media with bile salts (33). While dot blotting and hemagglutination assays have been used to confirm CS14 expression in some ETEC clinical isolates (15, 16, 33), additional studies were performed to confirm the major subunit expression and surface localization in the seven GEMS strains. CFA agar has historically been used for maximum expression for CFA/I and CS4 (56). We first grew the seven CS14+ ETEC isolates on CFA agar; however, there was no observed CS14 expression using Western blot analysis (Fig. 2). Previous studies have shown that addition of the iron chelator deferoxamine mesylate (DFOM) to the agar media increased CFA/I expression (69). Given the homology of CFA/I and CS14 and the identification of a putative upstream IscR binding site, we tested the effects of iron limitation on CS14 expression. For all seven ETEC strains, CS14 expression was observed when strains were grown on CFA agar with DFOM (200 µM). CsuA, the major subunit of CS14, was observed at 17 kDa (Fig. 2). *E. coli* (pBAD-CS14) was used as a control for CsuA expression. These data demonstrate growth condition-specific CS14 expression that requires low iron conditions in all ETEC isolates tested.

**Fig 3:**
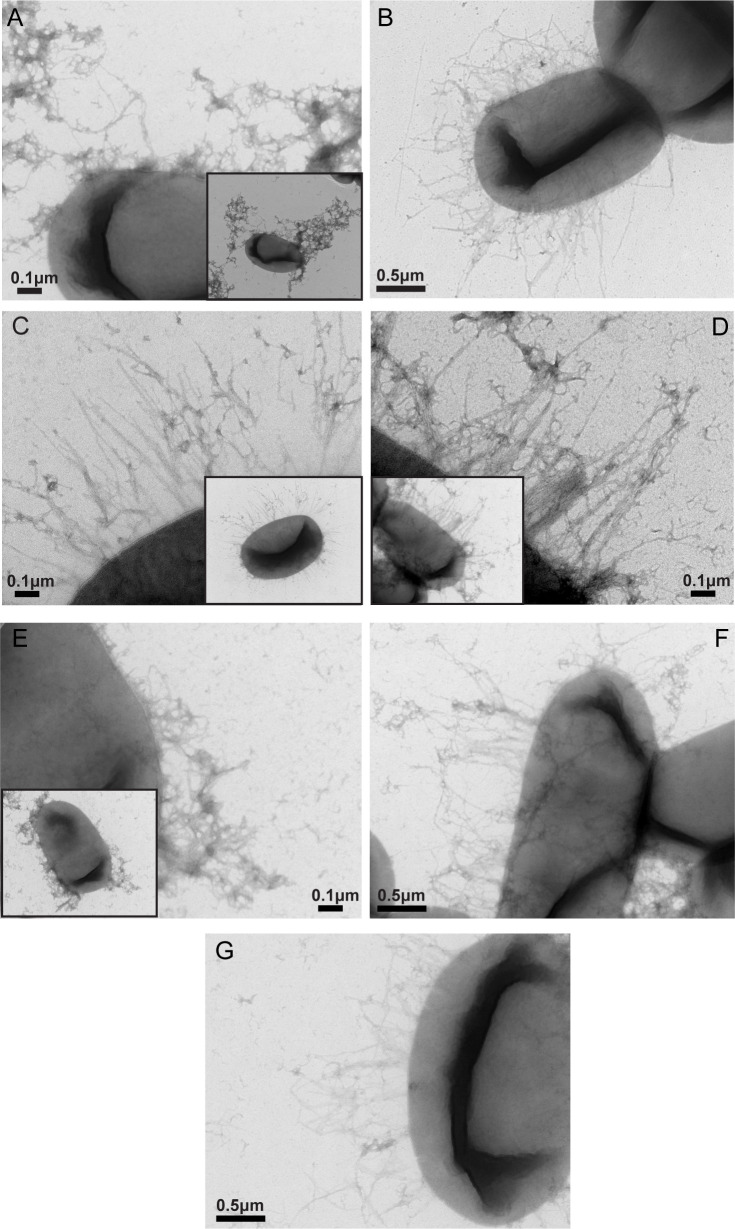
Surface expression of CS14 by GEMS clinical ETEC strains using negative staining and TEM. ETEC strains grown on CFA agar with DFOM (200 µM) were stained with uranyl acetate and visualized using TEM. Strains are as follows: 100576 (**A**), 200023 (**B**), 300316 (C), 400599 (**D**), 503825 (**E**), 602762 (**F**), and 700434 (**G**). Scale bar size indicated in micromolars (μM). The insets in panels A, C, D, and E show the whole bacterium producing the CF of interest.

**Fig 4:**
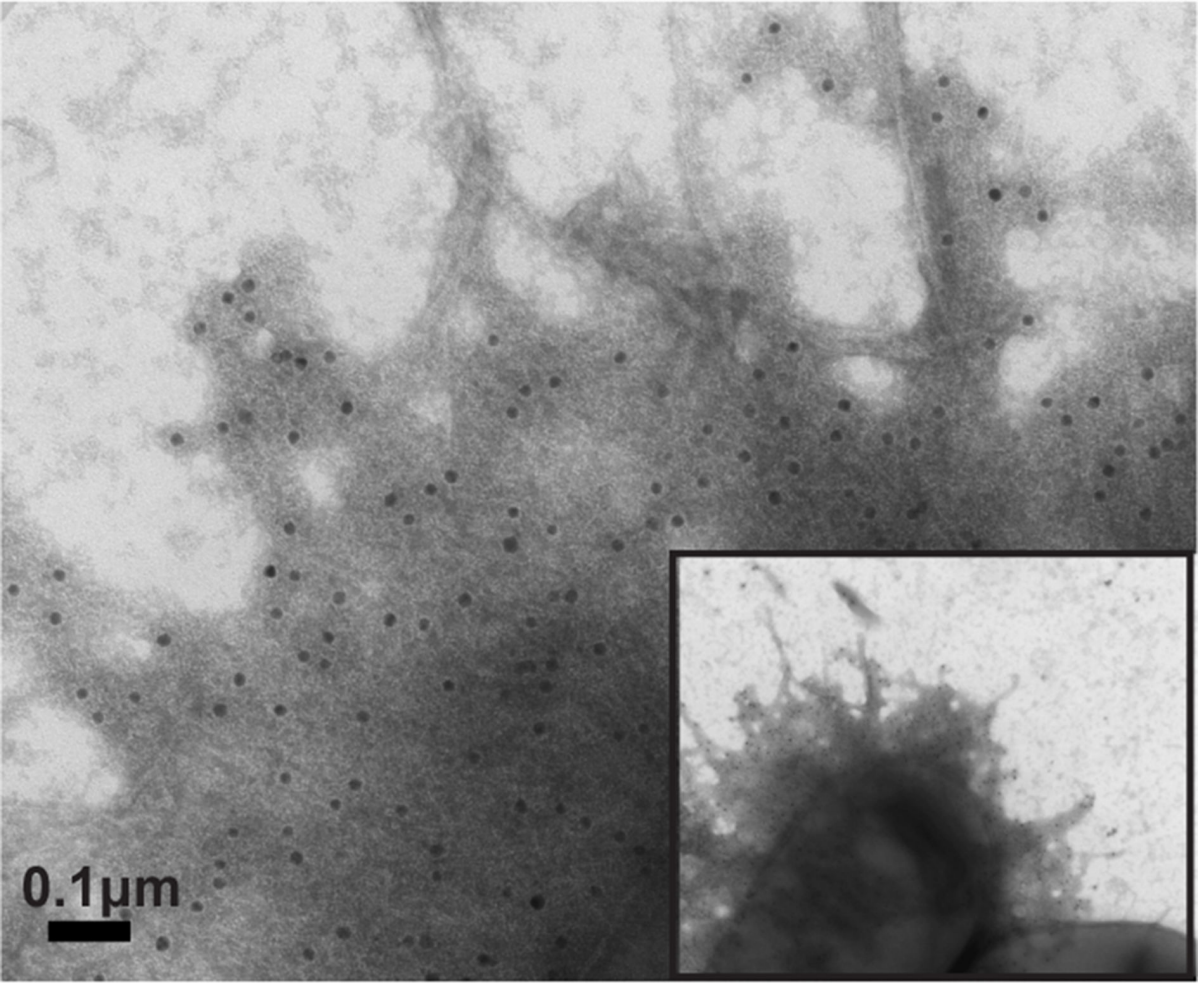
Surface expression of CS14 by ETEC strain 200023 using immunogold electron microscopy. ETEC strain 200023 was grown on CFA agar with DFOM (200 µM). The bacteria and CFs were visualized by TEM following immunogold staining with CS14-specific antibodies. Scale bar size indicated in micromolars (μM).

TEM was used to visualize surface expression of CS14 using negative staining and immunogold labeling. Strains were grown on CFA agar with DFOM (200 µM), and CF-specific expression was detected using anti-CS14 antibodies. In agreement with Western blot findings, CS14 fimbriae were observed on all ETEC clinical isolates (Fig. 3). CS14 was not observed on strains grown on CFA agar without DFOM (Fig. S5). Specific CS14 expression was confirmed on a representative ETEC strain 200023 using immunogold electron microscopy (Fig. 4), further supporting the surface expression of intact CS14 fimbriae on the ETEC clinical isolates.

### CS14 mediates adherence to human intestinal cell monolayers

**Fig 5:**
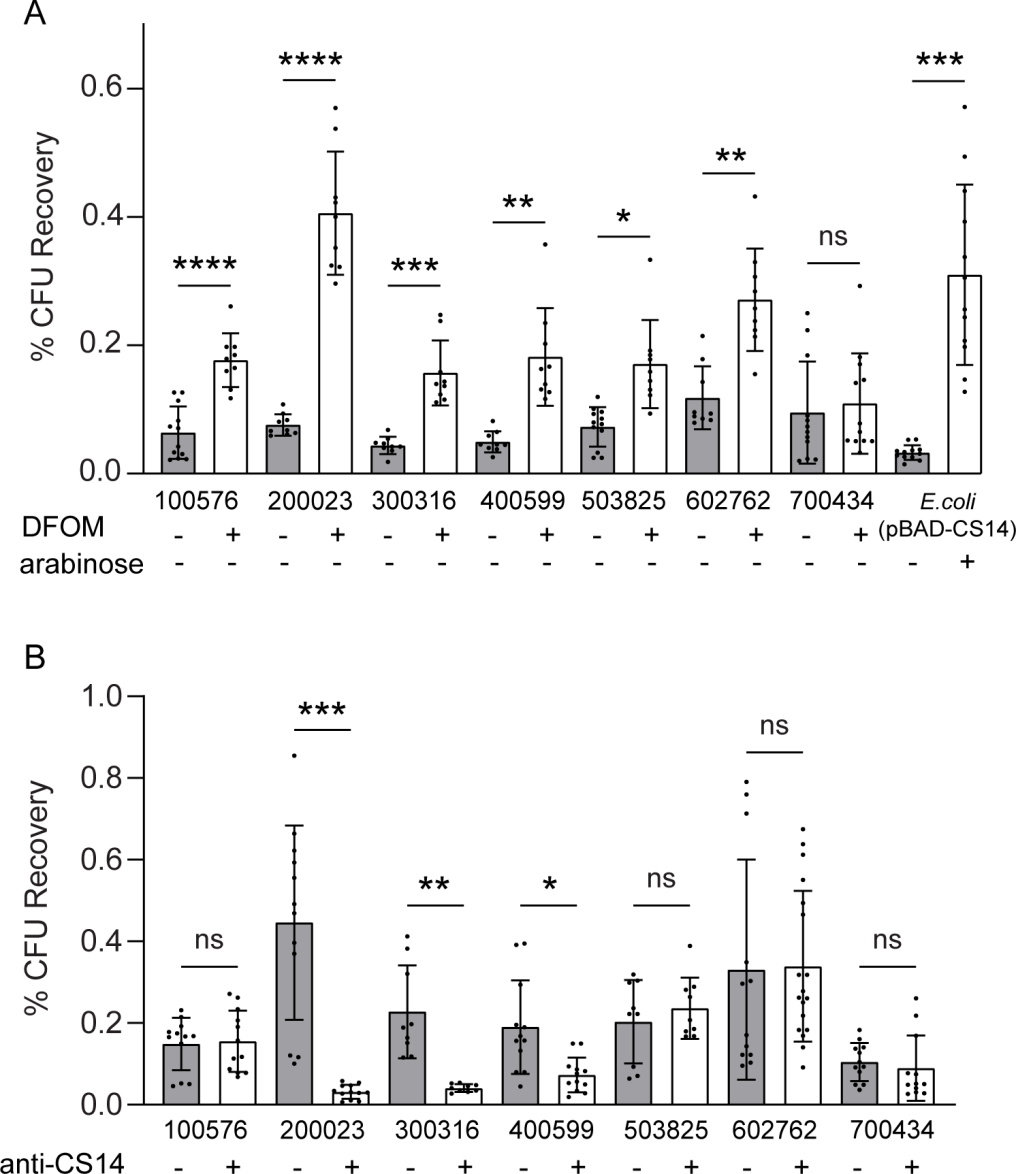
CS14 mediates adherence of ETEC to human intestinal cell monolayers. Human HT-29 cell monolayers were infected with wild-type ETEC strains grown on CFA agar with (+) or without (−) DFOM (200 µM) or with the control strain *E. coli* (pBAD-CS14) for 2 h (**A**). Human HT-29 cell monolayers were infected with wild-type ETEC strains for 2 h with or without pre-incubation with anti-CS14 antibodies (**B**). Monolayers were washed and lysed to quantify adherent bacteria expressed as percentage of initial inoculum (CFU recovery). Data presented are pooled from six independent experiments (**A**) or seven independent experiments (**B**). Each dot represents data collected from an individual monolayer. Error bars indicate standard deviations from the means. The asterisks above the bars indicate statistically significant differences determined using Welch ANOVA with Sidak multiple-comparison test. *P < 0.05; **P < 0.01; ***P < 0.001; ****P < 0.0001.

The contribution of CS14 to adherence was assessed using HT29 colonic cell monolayers. The clinical strains were grown on CFA agar with or without DFOM (200 µM) to control expression of CS14 (Fig. 2 to 4), and adherence was evaluated following 2 h of infection (Fig. 5). Adherence was significantly increased in six of seven ETEC clinical isolates grown on CFA with DFOM (200 µM) compared to those grown on CFA agar, demonstrating a global increase in adherence to monolayers by strains grown in iron-limiting conditions (Fig. 5A). The induction of CS14 expression by *E. coli* (pBAD-CS14) also resulted in significantly increased adherence to monolayers(Fig. 5A). One outlier ETEC strain 700434 did not exhibit increased adherence following growth in the presence of DFOM, despite evidence of CS14 expression by Western blotting and EM. This increase in adherence by CS14+ ETEC isolates was also observed when strains were grown on CFA agar and bile salts, a growth condition previously shown to induce CS14 expression (Fig. S6) (33).

In order to confirm CS14-specific adherence by these ETEC isolates, we quantified the adherence-blocking ability of CS14-specific antibodies against ETEC clinical isolates expressing CS14. Strains grown on CFA agar with DFOM (200 µM) were incubated with anti-CS14 antibody and used to infect HT29 cell monolayers for 2 h. Anti-CS14 antibody inhibited adherence of ETEC strains 200023, 300316, and 400599 by 92.9%, 82.1%, and 61.6%, respectively (Fig. 5B). Interestingly, the other four strains were not inhibited by anti-CS14 antibodies, which may be due to the expression of other important non-CF adhesins on the surface including EtpA (Table S1) (11, 73 – 77). Adhesion of the control strain *E. coli* (pBAD-CS14) was also inhibited by anti-CS14 antibody by 88.8% but not by non-specific rabbit antibodies (Fig. S7). These data demonstrate that CS14 drives adherence in some ETEC strains and in control *E. coli* exclusively expressing CS14 while other adhesins or virulence factors may be impacting ETEC adherence as well.

### Antibodies against class 5a fimbriae CS4, but not CFA/I, inhibit CS14-mediated adherence to intestinal cell monolayers

**Fig 6:**
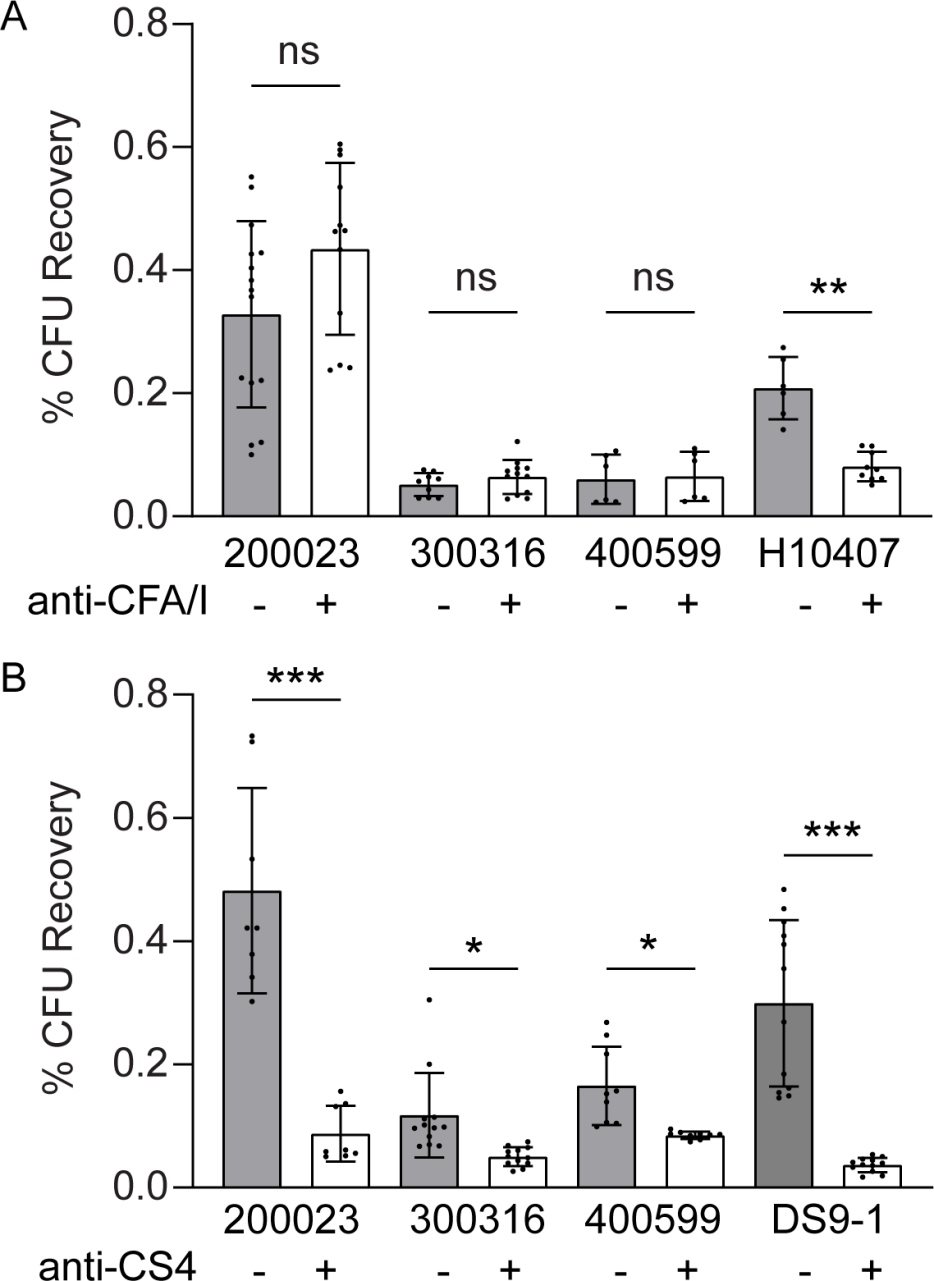
Antibodies to CS4, but not CFA/I, inhibit adherence by CS14-expressing ETEC to human intestinal cell monolayers. Human HT-29 cell monolayers were infected with wild-type ETEC strains 200023, 300316, and 400599 grown on CFA agar with DFOM (200 µM) for 2 h with or without pre-incubation with anti-CFA/I antibodies (**A**) or anti-CS4 antibodies (**B**). ETEC strains H10407 and DS9-1 were used as control strains for CFA/I- (**A**) and CS4-mediated adherence, respectively (**B**). Monolayers were washed and lysed to quantify adherent bacteria expressed as percentage of initial inoculum (CFU recovery). Data presented are pooled from eight independent experiments (**A**) or four independent experiments (**B**). Each dot represents data collected from an individual monolayer. Error bars indicate standard deviations from the means. The asterisks above the bars indicate statistically significant differences determined using Welch ANOVA with Sidak multiple-comparison test. *P < 0.05; **P < 0.01; ***P < 0.001.

Previous studies have reported the cross-reactivity of antibodies specific to class 5a fimbriae through antigen recognition using ELISA or ELISpot (23, 39); however, there are conflicting studies on the potential functional inhibition of these cross-reactive antibodies, particularly among the class 5 fimbriae (35, 39, 46). We quantified the adherence-blocking ability of CFA/I-specific or CS4-specific antibodies against ETEC clinical isolates expressing CS14 that were inhibited by the CS14-specific antibodies. Strains 200023, 300316, and 400599 were grown on CFA agar with DFOM (200 µM), incubated with either anti-CFA/I or anti-CS4 antibody, and used to infect HT29 cell monolayers for 2 h. Anti-CFA/I antibody did not inhibit adherence of any of the ETEC strains tested, as there was no significant difference in attachment between the CS14+ strains pre-incubated with or without antibody (Fig. 6A). Adherence of the control CFA/I + H10407 strain was inhibited by anti-CFA/I antibodies by 61.1% (Fig. 6A).

In contrast, anti-CS4 antibody inhibited adherence of ETEC 200023, 300316, and 400599 by 81.8%, 57.1%, and 48.6%, respectively (Fig. 6B). Adherence of the control CS4+ strain DS9-1 was inhibited by anti-CS4 antibodies by 87.7% (Fig. 6B). These data demonstrate that not all antibodies specific to class 5a fimbriae can inhibit adherence of CS14+ ETEC strains; however, these results are consistent with the high level of homology between CS4 and CS14 fimbriae, particularly in the tip adhesin (35).

### CS14 mediates adherence to human enteroid monolayers and is inhibited by antibodies against CS4 but not CFA/I

**Fig 7:**
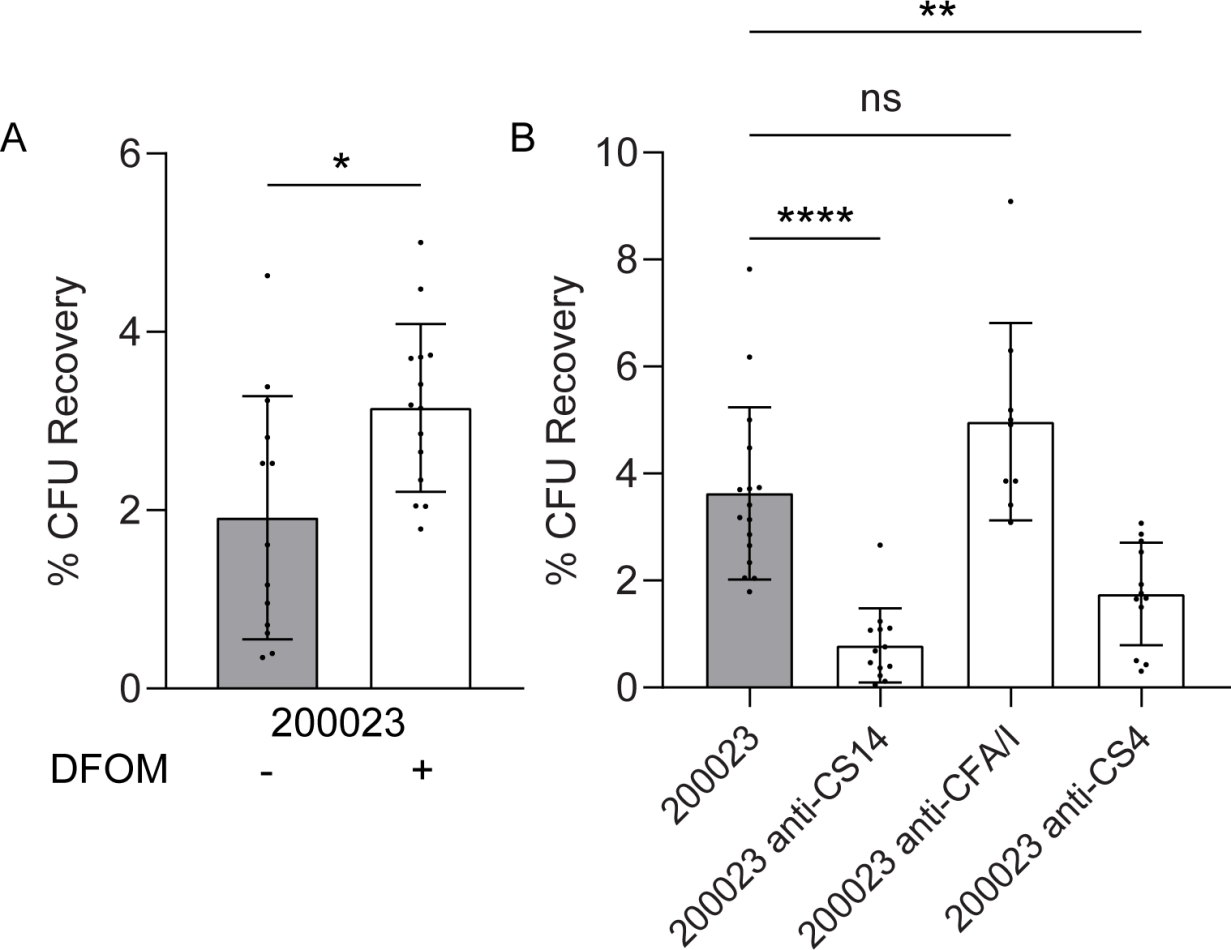
CS14 mediates adherence to human ileal enteroid monolayers. Differentiated ileal enteroid monolayers (46I) were infected with ETEC strain 200023 grown on CFA agar with (+) or without (−) DFOM (200 µM) for 4 h (**A**). Differentiated ileal enteroid monolayers (46I) were infected with ETEC strain 200023 grown on CFA agar with DFOM (200 µM) for 4 h with or without pre-incubation with anti-CS14, anti-CFA/I, or anti-CS4 antibodies (**B**). Monolayers were washed and lysed to quantify adherent bacteria expressed as percentage of initial inoculum (CFU recovery). Data presented are pooled from four independent experiments (**A**) and five independent experiments (**B**). Each dot represents data collected from an individual monolayer. Error bars indicate standard deviations from the means. The asterisks above the bars indicate statistically significant differences determined using one-way ANOVA with Bonferroni’s multiple-comparison test. *P < 0.05; **P < 0.01; ****P < 0.0001.

The human enteroid serves as the most relevant model to measure the effect of CS14 expression on adherence. Using the ileal enteroid line 46I, we first measured CS14-specific adherence of one representative CS14-expressing ETEC strain 200023 to enteroid monolayers. Strain 200023 was grown on CFA agar alone or CFA agar with DFOM (200 µM) for differential expression of CS14 (Fig. 2 to 4), and adherence was evaluated following 4 h of infection (Fig. 7). Similar to the increased adherence observed in HT29 cell monolayers, adherence was significantly increased by strain 200023 grown on CFA agar with DFOM (200 µM) compared to that grown on only CFA agar (Fig. 7A).

CS14-specific adherence to enteroid monolayers by ETEC strain 200023 was also tested by quantifying the adherence-blocking ability of CS14-specific antibodies. Strain 200023 was grown on CFA agar with DFOM (200 µM), incubated with anti-CS14 antibody, and used to infect enteroid monolayers for 4 h. Anti-CS14 antibody inhibited adherence of ETEC 200023 by 78.3% to 46I monolayers (Fig. 7B). We also tested the ability of the cross-reactive anti-CFA/I and anti-CS4 antibodies to inhibit the adherence of the strain 200023 after 4 h of infection. No adherence inhibition was observed when the strain 200023 was incubated with anti-CFA/I antibodies; however, a significant decrease in adherence was observed when incubated with anti-CS4 by 58.7% in 46I monolayers, supporting the cross-reactive functional inhibition of anti-CS4 antibodies observed in HT29 cell monolayers (Fig. 7B).

## DISCUSSION

This study emphasizes the importance and functional role of the minor CF CS14 in ETEC pathogenesis across global ETEC isolates and supports CS14 as a target antigen for inclusion in future ETEC vaccine strategies. CS14 was first identified as PCFO166, a unique 7nm fimbriae expressed by ST-only ETEC clinical isolates with the serotype O166 that could also hemagglutinate erythrocytes (16). Subsequent epidemiological studies in various geographical sites identified CS14 as a prevalently expressed CF in ETEC clinical isolates and detected anti-CS14 antibodies in human sera from infected individuals (17 – 32). Most recently, the GEMS reported that CS14 was the only individually expressed minor CF that reached a high prevalence (>5%) in ST-only ETEC cases and the only minor CF in ST-only and ST/LT ETEC strains that was significantly associated with MSD (15). This important association with MSD, and the frequent identification of this CF in disease-causing isolates prompted further investigation to its role in pathogenesis and its potential for consideration as a vaccine antigen.

Using the HT29 intestinal cell line and the human enteroid model, we demonstrated that CS14 expression resulted in increased adherence by a collection of recent global isolates. The one exception, ETEC strain 700434, is most likely due to differential regulation of CS14 or expression of other virulence factors. Our results are in agreement with earlier reports of adherence by CS14+ ETEC strains E7476A and WS3294A to Caco-2 cells or human enterocytes (35, 45). In our studies, the use of CFA agar supplemented with the iron chelator DFOM allowed for differential expression of CS14 in all strains as confirmed by Western blot and EM, establishing a new growth condition that induces CS14 expression similar to that when grown in CFA and SP1 media with bile salts. Growth in the presence of bile salts also resulted in significantly increased adherence by the CS14+ ETEC isolates tested. While the use of HT29 cells allowed a broad assessment of adherence, our results using human enteroid monolayers provided additional human relevant evidence for the role of CS14.

Previous studies investigated the expression of CS14 and the highly homologous CFA/I in different growth media. Lower levels of CS14 expression were observed following growth in SP1 media compared to that of CFA/I (33). While high levels of CFA/I are expressed following growth on CFA agar alone, CS14 expression required the addition of bile salts to CFA agar (16, 33, 38, 56). Another study determined that ETEC grown in iron-limiting conditions using the iron chelator DFOM in CFA broth resulted in increased CFA/I expression (69). Based on these reports, we demonstrated that CS14 was expressed when DFOM was present in CFA agar.

The effects of iron on CFA/I and CS14 expression suggest similar regulation, possibly through Rns and IscR. Rns-directed expression of CFA/I is mediated through two Rns binding sites in the upstream promoter region (71, 72, 78). Similarly, three Rns binding sites required for CS14 gene expression have been identified upstream of the CS14-encoding operon in the reference strain WS3294A (68). Our sequence analysis confirmed that all geographical CS14+ ETEC strains contained the three upstream Rns binding sites in the CS14 promoter similar to the reference strain. In addition to Rns, the regulator IscR has been shown to control the increased expression of CFA/I in ETEC H10407 under iron-limiting conditions (69). Other reports demonstrated that IscR also regulates additional ETEC genes (79). Our sequence analysis identified a putative IscR binding site in the upstream promoter region of all CS14 operons analyzed, which overlaps with the most promoter-proximal Rns binding site, similar to that of the CFA/I promoter (69, 71). While further studies are needed to confirm the direct regulation of CS14 expression by IscR, sequence analysis confirmed that the *iscR* gene was present in all strains. However, the presence of genes encoding Rns and IscR as well as their binding sites does not explain the differences in regulation between CFA/I and CS14 in these growth conditions. Regulation of CF expression is complex, and various growth conditions and regulators have been described to affect CF expression (9). Other key regulators that affect CF expression are present in the CS14+ ETEC strains, including Crp, Hns, CpxR, and FNR, and future studies will define the role of these regulators in CS14 expression (9, 58, 80).

Data supporting the importance of CS14 in ETEC clinical isolates underscore its consideration for inclusion in a broadly protective ETEC vaccine. Our data demonstrate that the sequences of the CS14-encoding operons are highly conserved among the seven geographically diverse ETEC isolates from the GEMS (>99.93%). This high level of operon sequence identity suggests that optimal antigen development would lead to efficient coverage by ETEC vaccines for CS14. Given the identification of four “hot spots” for SNPs in the tip gene *csuD* that ultimately impacts the protein sequence and is located in the N-terminal region associated with antigen-antibody binding (35), further studies are needed to understand the impact of specific differences in the tip adhesin and if these impact vaccine design, despite the high sequence identity in the rest of the CS14 operon.

Clinical trial data have shown that blocking ETEC adherence with anti-CF or anti-tip adhesin antibodies prevents diarrheal disease (81, 82). Our data demonstrated that polyclonal anti-CS14 antibodies inhibited adherence in three of the seven examined CS14+ GEMS strains (200023, 300316, and 400599) as well as the control *E. coli* (pBAD-CS14) strain. Given that the other four GEMS strains were not inhibited by anti-CS14 antibodies, it is possible that other non-CF adhesins are contributing to this increased adherence phenotype, including EtpA, FimH, or possible unidentified CFs (11, 73 – 77). The genes encoding these adhesins were identified in all strains using LSBSR analysis, and previous studies have shown that these genes are upregulated in low iron conditions in reference isolates (69). Further studies will assess the expression of these non-CF adhesins and test if they contribute to adherence in CS14+ ETEC strains.

Another possibility for the lack of inhibition of four GEMS strains by anti-CS14 antibodies is the sequence variation observed in the tip adhesin gene *csuD*. ETEC strains 100576, 503825, and 602762, which were not inhibited by anti-CS14 antibodies, all share the same SNP pattern in *csuD* (pattern 5), while the strain 700434 also has another SNP pattern (pattern 4), which are all different than the SNP patterns observed in strains 200023, 300316, and 400599 that were inhibited by anti-CS14 antibodies. Given that the anti-CS14 antibodies are polyclonal, further studies could determine the ability of anti-CS14 antibodies to recognize specific residues of the tip adhesin and how that contributes to functional inhibition.

Previous studies have shown cross-reactive antigen recognition of CS14 by CFA/I- and CS4-specific antibodies that were either monoclonal derived or antibodies isolated from humans, including vaccinated children and adults or those after natural infection (23, 39 – 43, 46). Many studies have focused on the cross-reactivity between CFA/I and CS14 specifically, using ELISA or hemagglutination inhibition assays (23, 35, 39 – 43, 46). However, cross-reactivity has not been shown to result in functional inhibition, including adherence inhibition to intestinal cells (35, 39, 46). We observed that adherence of a subset of CS14-expressing strains was inhibited by cross-reactive antibodies specific to another class 5a fimbriae CS4 but not CFA/I, using HT29 cells and the human enteroid model. This may be due to the higher level of operon nucleotide sequence identity between CS4 and CS14 (93.1%) compared to CS14 and CFA/I (90.7%) (34, 35, 61).

These antibody inhibition experiments were performed using polyclonal bivalent agglutinating anti-CF antibodies. Many studies have employed this approach to study adherence mediated by other CFs, including CS2 (83), CS3 (83), CS6 (64), and CS21 (84, 85). The ability of agglutinating antibodies to inhibit ETEC disease, such as those induced by vaccination, has also been demonstrated in human clinical trials (86). However, it is important to note the shortcomings of using agglutinating antibodies given the potential disruption of fimbrial physical properties including aggregation and steric hinderance (87). Other studies have used Fab fragments to definitively demonstrate CF component-specific binding inhibition (35, 42), and future studies will use Fab fragments specific to CFs of interest to minimize the impact on the fimbriae.

Adherence inhibition of CS14+ ETEC clinical isolates by anti-CS4 antibodies, but not anti-CFA/I antibodies, raises important consideration for inclusion of CF antigens in ETEC vaccine development. Some vaccine strategies include a CS4 antigen, while others do not (88 – 92). Given the prevalence of CS14 in ETEC isolated from humans with MSD, this study supports the inclusion of CS14 in future ETEC vaccine strategies. Inclusion of this CF would address the limited functional cross-reactivity between class 5 fimbrial antigens as well as continue research of CS14 in clinical isolates to understand its sequence conservation and the impact on vaccine design.

## Data Availability

Whole genome sequences for all ETEC strains in this study are available in GenBank (58).

## SUPPLEMENTAL MATERIAL

Fig. S1 msphere.00302-23-s0001.pdf Nucleotide alignment of CS14 operons analyzed in GEMS clinical ETEC isolates.

Fig. S2 msphere.00302-23-s0002.tif Amino acid alignment of CS14 operons analyzed in GEMS clinical ETEC isolates.

Fig. S3 msphere.00302-23-s0003.pdf Alignment of *csuD* gene in 91 ETEC strains.

Fig. S4 msphere.00302-23-s0004.tif Nucleotide alignment of CS14 promoter and upstream region from GEMS clinical ETEC isolates.

Fig. S5 msphere.00302-23-s0005.png CS14 surface expression in strains grown without iron chelation.

Fig. S6 msphere.00302-23-s0006.tif CS14 mediates adherence of ETEC grown in bile salts to human intestinal cell monolayers.

Fig. S7 msphere.00302-23-s0007.tif Antibody inhibition of adherence by *E. coli*(pBAD-CS14) to human intestinal cell monolayers.

Legends msphere.00302-23-s0008.docx Supplemental figure legends.

Supplemental Tables msphere.00302-23-s0009.docx Tables S1 and S2.

ASM does not own the copyrights to Supplemental Material that may be linked to, or accessed through, an article. The authors have granted ASM a non-exclusive, world-wide license to publish the Supplemental Material files. Please contact the corresponding author directly for reuse.
