# Supplementary material for: The role of the minor colonization factor CS14 in adherence to intestinal cell models by geographically diverse ETEC isolates
Source: mSphere. 2023 Oct 3;8(5):e00302-23. doi: 10.1128/msphere.00302-23 (PMC10597352; doi:10.1128/msphere.00302-23)
Supplement: Fig. S1 — Nucleotide alignment of CS14 operons analyzed in GEMS clinical ETEC isolates. [file msphere.00302-23-s0001.pdf]

```
CLUSTAL format alignment by MAFFT FFT-NS-i (v7.487)


AY283611.1      TGAGTCCGTCGAAACGGCGGTTACTGGAAGAGGTGGTGTACGTCATAACGGAGACGGTGA
100576_CS14     ------------------------------------------------------------
503825_CS14     ------------------------------------------------------------
200023_CS14     ------------------------------------------------------------
300316_CS14     ------------------------------------------------------------
700434_CS14     ------------------------------------------------------------
602762_CS14     ------------------------------------------------------------
400599_CS14     ------------------------------------------------------------


AY283611.1      GAAAAACGGCGATGCAAATCAGGTGGAGAGGGATGTATCAGCGGTTACTGAAGGTTGACC
100576_CS14     ------------------------------------------------------------
503825_CS14     ------------------------------------------------------------
200023_CS14     ------------------------------------------------------------
300316_CS14     ------------------------------------------------------------
700434_CS14     ------------------------------------------------------------
602762_CS14     ------------------------------------------------------------
400599_CS14     ------------------------------------------------------------


AY283611.1      CGCTGAAATGCATTCTGTGCGGAAGTCAGATGCGGTTTACGGGACTGAAGAGGGGCTACC
100576_CS14     ------------------------------------------------------------
503825_CS14     ------------------------------------------------------------
200023_CS14     ------------------------------------------------------------
300316_CS14     ------------------------------------------------------------
700434_CS14     ------------------------------------------------------------
602762_CS14     ------------------------------------------------------------
400599_CS14     ------------------------------------------------------------


AY283611.1      GTCTGACAGCTATGATGCATAAGCCCCTGGCCCGGAAGCGGTGGTGCGGCTGAGAGCCAC
100576_CS14     --------------------------------------CGGTGGTGCGGCTGAGAGCCAC
503825_CS14     --------------------------------------CGGTGGTGCGGCTGAGAGCCAC
200023_CS14     --------------------------------------CGGTGGTGCGGCTGAGAGCCAC
300316_CS14     --------------------------------------CGGTGGTGCGGCTGAGAGCCAC
700434_CS14     --------------------------------------CGGTGGTGCGGCTGAGAGCCAC
602762_CS14     ---------------------------------------GGTGGTGCGGCTGAGAGCCAC
400599_CS14     ---------------------------------------GGTGGTGCGGCTGAGAGCCAC
                                                       *********************

AY283611.1      AGAGGGGAAGTTACGTCCATTTTTCGGGGAATGGAGCAAAAAACCATCAGATATCCCCTG
100576_CS14     AGAGGGGAAGTTACGTCCATTTTTCGGGGAATGGAGCAAAAAACCATCAGATATCCCCTG
503825_CS14     AGAGGGGAAGTTACGTCCATTTTTCGGGGAATGGAGCAAAAAACCATCAGATATCCCCTG
200023_CS14     AGAGGGGAAGTTACGTCCATTTTTCGGGGAATGGAGCAAAAAACCATCAGATATCCCCTG
300316_CS14     AGAGGGGAAGTTACGTCCATTTTTCGGGGAATGGAGCAAAAAACCATCAGATATCCCCTG
700434_CS14     AGAGGGGAAGTTACGTCCATTTTTCGGGGAATGGAGCAAAAAACCATCAGATATCCCCTG
602762_CS14     AGAGGGGAAGTTACGTCCATTTTTCGGGGAATGGAGCAAAAAACCATCAGATATCCCCTG
400599_CS14     AGAGGGGAAGTTACGTCCATTTTTCGGGGAATGGAGCAAAAAACCATCAGATATCCCCTG
                ************************************************************

AY283611.1      CATCAATCGATGAGCACCATTTAATGGTCGTGGTCGTGCACATGGCGCACAGAGAGTGTT
100576_CS14     CATCAATCGATGAGCACCATTTAATGGTCGTGGTCGTGCACATGGCGCACAGAGAGTGTT
503825_CS14     CATCAATCGATGAGCACCATTTAATGGTCGTGGTCGTGCACATGGCGCACAGAGAGTGTT
200023_CS14     CATCAATCGATGAGCACCATTTAATGGTCGTGGTCGTGCACATGGCGCACAGAGAGTGTT
300316_CS14     CATCAATCGATGAGCACCATTTAATGGTCGTGGTCGTGCACATGGCGCACAGAGAGTGTT
700434_CS14     CATCAATCGATGAGCACCATTTAATGGTCGTGGTCGTGCACATGGCGCACAGAGAGTGTT
602762_CS14     CATCAATCGATGAGCACCATTTAATGGTCGTGGTCGTGCACATGGCGCACAGAGAGTGTT
400599_CS14     CATCAATCGATGAGCACCATTTAATGGTCGTGGTCGTGCACATGGCGCACAGAGAGTGTT
                ************************************************************

AY283611.1      GAATAAATATCCTTTTTCAGGTTTTTTTAATCTTTTTGATATTTAAATCCCTATCAATGA
```

```
100576_CS14     GAATAAATATCCTTTTTCAGGTTTTTTTAATCTTTTTGATATTTAAATCCCTATCAATGA
503825_CS14     GAATAAATATCCTTTTTCAGGTTTTTTTAATCTTTTTGATATTTAAATCCCTATCAATGA
200023_CS14     GAATAAATATCCTTTTTCAGGTTTTTTTAATCTTTTTGATATTTAAATCCCTATCAATGA
300316_CS14     GAATAAATATCCTTTTTCAGGTTTTTTTAATCTTTTTGATATTTAAATCCCTATCAATGA
700434_CS14     GAATAAATATCCTTTTTCAGGTTTTTTTAATCTTTTTGATATTTAAATCCCTATCAATGA
602762_CS14     GAATAAATATCCTTTTTCAGGTTTTTTTAATCTTTTTGATATTTAAATCCCTATCAATGA
400599_CS14     GAATAAATATCCTTTTTCAGGTTTTTTTAATCTTTTTGATATTTAAATCCCTATCAATGA
                ************************************************************

AY283611.1      ACATCGCAGTATTAAATACAAAAATAGCACAGATGACACAGATGATTTATTGCATTGTCT
100576_CS14     ACATCGCAGTATTAAATACAAAAATAGCACAGATGACACA-ATGATTTATTGCATTGTCT
503825_CS14     ACATCGCAGTATTAAATACAAAAATAGCACAGATGACACA-ATGATTTATTGCATTGTCT
200023_CS14     ACATCGCAGTATTAAATACAAAAATAGCACAGATGACACA-ATGATTTATTGCATTGTCT
300316_CS14     ACATCGCAGTATTAAATACAAAAATAGCACAGATGACACA-ATGATTTATTGCATTGTCT
700434_CS14     ACATCGCAGTATTAAATACAAAAATAGCACAGATGACACA-ATGATTTATTGCATTGTCT
602762_CS14     ACATCGCAGTATTAAATACAAAAATAGCACAGATGACACA-ATGATTTATTGCATTGTCT
400599_CS14     ACATCGCAGTATTAAATACAAAAATAGCACAGATGACACA-ATGATTTATTGCATTGTCT
                **************************************** *******************

AY283611.1      TGTTTTTTTATCTCA-TTTTTTTTTGCTTTGATTTATCGTTGATGAAAGCTCAGGAGGGA
100576_CS14     TGTTTTTTTATCTCA-TTTTTTTTTGCTTTGATTTATCGTTGATGAAAGCTCAGGAGGGA
503825_CS14     TGTTTTTTTATCTCA-TTTTTTTTTGCTTTGATTTATCGTTGATGAAAGCTCAGGAGGGA
200023_CS14     TGTTTTTTTATCTCA-TTTTTTTTTGCTTTGATTTATCGTTGATGAAAGCTCAGGAGGGA
300316_CS14     TGTTTTTTTATCTCA-TTTTTTTTTGCTTTGATTTATCGTTGATGAAAGCTCAGGAGGGA
700434_CS14     TGTTTTTTTATCTCA-TTTTTTTTTGCTTTGATTTATCGTTGATGAAAGCTCAGGAGGGA
602762_CS14     TGTTTTTTTATCTCATTTTTTTTTTGCTTTGATTTATCGTTGATGAAAGCTCAGGAGGGA
400599_CS14     TGTTTTTTTATCTCA-TTTTTTTTTGCTTTGATTTATCGTTGATGAAAGCTCAGGAGGGA
                *************** ********************************************

AY283611.1      ATATGCATAAATTATTTTATTTACTAAGTTTACTCATGGCTCCATTTGTTGCAAATGCAA
100576_CS14     ATATGCATAAATTATTTTATTTACTAAGTTTACTCATGGCTCCATTTGTTGCAAATGCAA
503825_CS14     ATATGCATAAATTATTTTATTTACTAAGTTTACTCATGGCTCCATTTGTTGCAAATGCAA
200023_CS14     ATATGCATAAATTATTTTATTTACTAAGTTTACTCATGGCTCCATTTGTTGCAAATGCAA
300316_CS14     ATATGCATAAATTATTTTATTTACTAAGTTTACTCATGGCTCCATTTGTTGCAAATGCAA
700434_CS14     ATATGCATAAATTATTTTATTTACTAAGTTTACTCATGGCTCCATTTGTTGCAAATGCAA
602762_CS14     ATATGCATAAATTATTTTATTTACTAAGTTTACTCATGGCTCCATTTGTTGCAAATGCAA
400599_CS14     ATATGCATAAATTATTTTATTTACTAAGTTTACTCATGGCTCCATTTGTTGCAAATGCAA
                ************************************************************

AY283611.1      ACTTTATGATATATCCAATATCAAAAGATTTAAAGAATGGAAATAGTGAGTTAGTTCGTA
100576_CS14     ACTTTATGATATATCCAATATCAAAAGATTTAAAGAATGGAAATAGTGAGTTAGTTCGTA
503825_CS14     ACTTTATGATATATCCAATATCAAAAGATTTAAAGAATGGAAATAGTGAGTTAGTTCGTA
200023_CS14     ACTTTATGATATATCCAATATCAAAAGATTTAAAGAATGGAAATAGTGAGTTAGTTCGTA
300316_CS14     ACTTTATGATATATCCAATATCAAAAGATTTAAAGAATGGAAATAGTGAGTTAGTTCGTA
700434_CS14     ACTTTATGATATATCCAATATCAAAAGATTTAAAGAATGGAAATAGTGAGTTAGTTCGTA
602762_CS14     ACTTTATGATATATCCAATATCAAAAGATTTAAAGAATGGAAATAGTGAGTTAGTTCGTA
400599_CS14     ACTTTATGATATATCCAATATCAAAAGATTTAAAGAATGGAAATAGTGAGTTAGTTCGTA
                ************************************************************

AY283611.1      TTTATTCAAAATCAAAAGAGATACAATATATAAAAATATATACAAAAAAGATTATTAATC
100576_CS14     TTTATTCAAAATCAAAAGAGATACAATATATAAAAATATATACAAAAAAGATTATTAATC
503825_CS14     TTTATTCAAAATCAAAAGAGATACAATATATAAAAATATATACAAAAAAGATTATTAATC
200023_CS14     TTTATTCAAAATCAAAAGAGATACAATATATAAAAATATATACAAAAAAGATTATTAATC
300316_CS14     TTTATTCAAAATCAAAAGAGATACAATATATAAAAATATATACAAAAAAGATTATTAATC
700434_CS14     TTTATTCAAAATCAAAAGAGATACAATATATAAAAATATATACAAAAAAGATTATTAATC
602762_CS14     TTTATTCAAAATCAAAAGAGATACAATATATAAAAATATATACAAAAAAGATTATTAATC
400599_CS14     TTTATTCAAAATCAAAAGAGATACAATATATAAAAATATATACAAAAAAGATTATTAATC
                ************************************************************

AY283611.1      CCGGTACAACTGAAGAACATGAAGTTGATATGCCCAATTGGGATGGTGGGTTTGTGGTCA
100576_CS14     CCGGTACAACTGAAGAACATGAAGTTGATATGCCCAATTGGGATGGTGGGTTTGTGGTCA
503825_CS14     CCGGTACAACTGAAGAACATGAAGTTGATATGCCCAATTGGGATGGTGGGTTTGTGGTCA
200023_CS14     CCGGTACAACTGAAGAACATGAAGTTGATATGCCCAATTGGGATGGTGGGTTTGTGGTCA
300316_CS14     CCGGTACAACTGAAGAACATGAAGTTGATATGCCCAATTGGGATGGTGGGTTTGTGGTCA
700434_CS14     CCGGTACAACTGAAGAACATGAAGTTGATATGCCCAATTGGGATGGTGGGTTTGTGGTCA
```

```
602762_CS14     CCGGTACAACTGAAGAACATGAAGTTGATATGCCCAATTGGGATGGTGGGTTTGTGGTCA
400599_CS14     CCGGTACAACTGAAGAACATGAAGTTGATATGCCCAATTGGGATGGTGGGTTTGTGGTCA
                ************************************************************

AY283611.1      CTCCTCAGAAAGTTATCCTTCCTGCAGGAGCGAGTAAATCAATACGTTTAACTCAATTTA
100576_CS14     CTCCTCAGAAAGTTATCCTTCCTGCAGGAGCGAGTAAATCAATACGTTTAACTCAATTTA
503825_CS14     CTCCTCAGAAAGTTATCCTTCCTGCAGGAGCGAGTAAATCAATACGTTTAACTCAATTTA
200023_CS14     CTCCTCAGAAAGTTATCCTTCCTGCAGGAGCGAGTAAATCAATACGTTTAACTCAATTTA
300316_CS14     CTCCTCAGAAAGTTATCCTTCCTGCAGGAGCGAGTAAATCAATACGTTTAACTCAATTTA
700434_CS14     CTCCTCAGAAAGTTATCCTTCCTGCAGGAGCGAGTAAATCAATACGTTTAACTCAATTTA
602762_CS14     CTCCTCAGAAAGTTATCCTTCCTGCAGGAGCGAGTAAATCAATACGTTTAACTCAATTTA
400599_CS14     CTCCTCAGAAAGTTATCCTTCCTGCAGGAGCGAGTAAATCAATACGTTTAACTCAATTTA
                ************************************************************

AY283611.1      AAATACCAAAAAAAGAGGAAGTTTATAGAGTATATTTTGAGGCGGTAAAACCAGATAGCA
100576_CS14     AAATACCAAAAAAAGAGGAAGTTTATAGAGTATATTTTGAGGCGGTAAAACCAGATAGCA
503825_CS14     AAATACCAAAAAAAGAGGAAGTTTATAGAGTATATTTTGAGGCGGTAAAACCAGATAGCA
200023_CS14     AAATACCAAAAAAAGAGGAAGTTTATAGAGTATATTTTGAGGCGGTAAAACCAGATAGCA
300316_CS14     AAATACCAAAAAAAGAGGAAGTTTATAGAGTATATTTTGAGGCGGTAAAACCAGATAGCA
700434_CS14     AAATACCAAAAAAAGAGGAAGTTTATAGAGTATATTTTGAGGCGGTAAAACCAGATAGCA
602762_CS14     AAATACCAAAAAAAGAGGAAGTTTATAGAGTATATTTTGAGGCGGTAAAACCAGATAGCA
400599_CS14     AAATACCAAAAAAAGAGGAAGTTTATAGAGTATATTTTGAGGCGGTAAAACCAGATAGCA
                ************************************************************

AY283611.1      AAGAAAATGTAATTGATAATAAAAAACTAACAACAGAACTATCTGTTAATATAATCTATG
100576_CS14     AAGAAAATGTAATTGATAATAAAAAACTAACAACAGAACTATCTGTTAATATAATCTATG
503825_CS14     AAGAAAATGTAATTGATAATAAAAAACTAACAACAGAACTATCTGTTAATATAATCTATG
200023_CS14     AAGAAAATGTAATTGATAATAAAAAACTAACAACAGAACTATCTGTTAATATAATCTATG
300316_CS14     AAGAAAATGTAATTGATAATAAAAAACTAACAACAGAACTATCTGTTAATATAATCTATG
700434_CS14     AAGAAAATGTAATTGATAATAAAAAACTAACAACAGAACTATCTGTTAATATAATCTATG
602762_CS14     AAGAAAATGTAATTGATAATAAAAAACTAACAACAGAACTATCTGTTAATATAATCTATG
400599_CS14     AAGAAAATGTAATTGATAATAAAAAACTAACAACAGAACTATCTGTTAATATAATCTATG
                ************************************************************

AY283611.1      CGGCTCTAATCAGATCTTTACCAAGTGAACAAAATATATCACTAAATATTTCTAGAAATG
100576_CS14     CGGCTCTAATCAGATCTTTACCAAGTGAACAAAATATATCACTAAATATTTCTAGAAATG
503825_CS14     CGGCTCTAATCAGATCTTTACCAAGTGAACAAAATATATCACTAAATATTTCTAGAAATG
200023_CS14     CGGCTCTAATCAGATCTTTACCAAGTGAACAAAATATATCACTAAATATTTCTAGAAATG
300316_CS14     CGGCTCTAATCAGATCTTTACCAAGTGAACAAAATATATCACTAAATATTTCTAGAAATG
700434_CS14     CGGCTCTAATCAGATCTTTACCAAGTGAACAAAATATATCACTAAATATTTCTAGAAATG
602762_CS14     CGGCTCTAATCAGATCTTTACCAAGTGAACAAAATATATCACTAAATATTTCTAGAAATG
400599_CS14     CGGCTCTAATCAGATCTTTACCAAGTGAACAAAATATATCACTAAATATTTCTAGAAATG
                ************************************************************

AY283611.1      CAAAAAAAAATATAATTATTTATAATAACGGGAATGTTAGAGCAGGTGTTAAAGATATTT
100576_CS14     CAAAAAAAAATATAATTATTTATAATAACGGGAATGTTAGAGCAGGTGTTAAAGATATTT
503825_CS14     CAAAAAAAAATATAATTATTTATAATAACGGGAATGTTAGAGCAGGTGTTAAAGATATTT
200023_CS14     CAAAAAAAAATATAATTATTTATAATAACGGGAATGTTAGAGCAGGTGTTAAAGATATTT
300316_CS14     CAAAAAAAAATATAATTATTTATAATAACGGGAATGTTAGAGCAGGTGTTAAAGATATTT
700434_CS14     CAAAAAAAAATATAATTATTTATAATAACGGGAATGTTAGAGCAGGTGTTAAAGATATTT
602762_CS14     CAAAAAAAAATATAATTATTTATAATAACGGGAATGTTAGAGCAGGTGTTAAAGATATTT
400599_CS14     CAAAAAAAAATATAATTATTTATAATAACGGGAATGTTAGAGCAGGTGTTAAAGATATTT
                ************************************************************

AY283611.1      ATTTTTGTAAGTCATCTAATATCGATGATAACTGTGTAAAAAAAGCGCATAACAAGAATA
100576_CS14     ATTTTTGTAAGTCATCTAATATCGATGATAACTGTGTAAAAAAAGCGCATAACAAGAATA
503825_CS14     ATTTTTGTAAGTCATCTAATATCGATGATAACTGTGTAAAAAAAGCGCATAACAAGAATA
200023_CS14     ATTTTTGTAAGTCATCTAATATCGATGATAACTGTGTAAAAAAAGCGCATAACAAGAATA
300316_CS14     ATTTTTGTAAGTCATCTAATATCGATGATAACTGTGTAAAAAAAGCGCATAACAAGAATA
700434_CS14     ATTTTTGTAAGTCATCTAATATCGATGATAACTGTGTAAAAAAAGCGCATAACAAGAATA
602762_CS14     ATTTTTGTAAGTCATCTAATATCGATGATAACTGTGTAAAAAAAGCGCATAACAAGAATA
400599_CS14     ATTTTTGTAAGTCATCTAATATCGATGATAACTGTGTAAAAAAAGCGCATAACAAGAATA
                ************************************************************

AY283611.1      TATATCCAGAAAAGTCATTTGATACGCTGGTTAATAACAATTTTTCTTATGTTTTCATTA
```

```
100576_CS14     TATATCCAGAAAAGTCATTTGATACGCTGGTTAATAACAATTTTTCTTATGTTTTCATTA
503825_CS14     TATATCCAGAAAAGTCATTTGATACGCTGGTTAATAACAATTTTTCTTATGTTTTCATTA
200023_CS14     TATATCCAGAAAAGTCATTTGATACGCTGGTTAATAACAATTTTTCTTATGTTTTCATTA
300316_CS14     TATATCCAGAAAAGTCATTTGATACGCTGGTTAATAACAATTTTTCTTATGTTTTCATTA
700434_CS14     TATATCCAGAAAAGTCATTTGATACGCTGGTTAATAACAATTTTTCTTATGTTTTCATTA
602762_CS14     TATATCCAGAAAAGTCATTTGATACGCTGGTTAATAACAATTTTTCTTATGTTTTCATTA
400599_CS14     TATATCCAGAAAAGTCATTTGATACGCTGGTTAATAACAATTTTTCTTATGTTTTCATTA
                ************************************************************

AY283611.1      AATTAAACCATGAAGGCATAGAAAAAGAACAAGGGCTAATACAATTAAAAGTTCCTTGAT
100576_CS14     AATTAAACCATGAAGGCATAGAAAAAGAACAAGGGCTAATACAATTAAAAGTTCCTTGAT
503825_CS14     AATTAAACCATGAAGGCATAGAAAAAGAACAAGGGCTAATACAATTAAAAGTTCCTTGAT
200023_CS14     AATTAAACCATGAAGGCATAGAAAAAGAACAAGGGCTAATACAATTAAAAGTTCCTTGAT
300316_CS14     AATTAAACCATGAAGGCATAGAAAAAGAACAAGGGCTAATACAATTAAAAGTTCCTTGAT
700434_CS14     AATTAAACCATGAAGGCATAGAAAAAGAACAAGGGCTAATACAATTAAAAGTTCCTTGAT
602762_CS14     AATTAAACCATGAAGGCATAGAAAAAGAACAAGGGCTAATACAATTAAAAGTTCCTTGAT
400599_CS14     AATTAAACCATGAAGGCATAGAAAAAGAACAAGGGCTAATACAATTAAAAGTTCCTTGAT
                ************************************************************

AY283611.1      TACTCATCTATATACTAAGGAGTTCTAATGAAATTAAAAAAAACTATTGGCGCAATGGCT
100576_CS14     TACTCATCTATATACTAAGGAGTTCTAATGAAATTAAAAAAAACTATTGGCGCAATGGCT
503825_CS14     TACTCATCTATATACTAAGGAGTTCTAATGAAATTAAAAAAAACTATTGGCGCAATGGCT
200023_CS14     TACTCATCTATATACTAAGGAGTTCTAATGAAATTAAAAAAAACTATTGGCGCAATGGCT
300316_CS14     TACTCATCTATATACTAAGGAGTTCTAATGAAATTAAAAAAAACTATTGGCGCAATGGCT
700434_CS14     TACTCATCTATATACTAAGGAGTTCTAATGAAATTAAAAAAAACTATTGGCGCAATGGCT
602762_CS14     TACTCATCTATATACTAAGGAGTTCTAATGAAATTAAAAAAAACTATTGGCGCAATGGCT
400599_CS14     TACTCATCTATATACTAAGGAGTTCTAATGAAATTAAAAAAAACTATTGGCGCAATGGCT
                ************************************************************

AY283611.1      CTGAGCACAATATTTGTAGCGGTGAGTGCTTCAGCAGTAGAGAAAAATATTACTGTGACA
100576_CS14     CTGAGCACAATATTTGTAGCGGTGAGTGCTTCAGCAGTAGAGAAAAATATTACTGTGACA
503825_CS14     CTGAGCACAATATTTGTAGCGGTGAGTGCTTCAGCAGTAGAGAAAAATATTACTGTGACA
200023_CS14     CTGAGCACAATATTTGTAGCGGTGAGTGCTTCAGCAGTAGAGAAAAATATTACTGTGACA
300316_CS14     CTGAGCACAATATTTGTAGCGGTGAGTGCTTCAGCAGTAGAGAAAAATATTACTGTGACA
700434_CS14     CTGAGCACAATATTTGTAGCGGTGAGTGCTTCAGCAGTAGAGAAAAATATTACTGTGACA
602762_CS14     CTGAGCACAATATTTGTAGCGGTGAGTGCTTCAGCAGTAGAGAAAAATATTACTGTGACA
400599_CS14     CTGAGCACAATATTTGTAGCGGTGAGTGCTTCAGCAGTAGAGAAAAATATTACTGTGACA
                ************************************************************

AY283611.1      GCCAGTGTTGATCCTACTATTGATATTCTTCAAGCAAATGGTTCTGCGCTACCGACAGCT
100576_CS14     GCCAGTGTTGATCCTACTATTGATATTCTTCAAGCAAATGGTTCTGCGCTACCGACAGCT
503825_CS14     GCCAGTGTTGATCCTACTATTGATATTCTTCAAGCAAATGGTTCTGCGCTACCGACAGCT
200023_CS14     GCCAGTGTTGATCCTACTATTGATATTCTTCAAGCAAATGGTTCTGCGCTACCGACAGCT
300316_CS14     GCCAGTGTTGATCCTACTATTGATATTCTTCAAGCAAATGGTTCTGCGCTACCGACAGCT
700434_CS14     GCCAGTGTTGATCCTACTATTGATATTCTTCAAGCAAATGGTTCTGCGCTACCGACAGCT
602762_CS14     GCCAGTGTTGATCCTACTATTGATATTCTTCAAGCAAATGGTTCTGCGCTACCGACAGCT
400599_CS14     GCCAGTGTTGATCCTACTATTGATATTCTTCAAGCAAATGGTTCTGCGCTACCGACAGCT
                ************************************************************

AY283611.1      GTAGATTTAACTTATCTACCTGGTGCAAAAACTTTTGAAAATTACAGTGTTCTAACCCAG
100576_CS14     GTAGATTTAACTTATCTACCTGGTGCAAAAACTTTTGAAAATTACAGTGTTCTAACCCAG
503825_CS14     GTAGATTTAACTTATCTACCTGGTGCAAAAACTTTTGAAAATTACAGTGTTCTAACCCAG
200023_CS14     GTAGATTTAACTTATCTACCTGGTGCAAAAACTTTTGAAAATTACAGTGTTCTAACCCAG
300316_CS14     GTAGATTTAACTTATCTACCTGGTGCAAAAACTTTTGAAAATTACAGTGTTCTAACCCAG
700434_CS14     GTAGATTTAACTTATCTACCTGGTGCAAAAACTTTTGAAAATTACAGTGTTCTAACCCAG
602762_CS14     GTAGATTTAACTTATCTACCTGGTGCAAAAACTTTTGAAAATTACAGTGTTCTAACCCAG
400599_CS14     GTAGATTTAACTTATCTACCTGGTGCAAAAACTTTTGAAAATTACAGTGTTCTAACCCAG
                ************************************************************

AY283611.1      ATTTACACAAATGACCCTTCAAAAGGTTTAGATGTTCGACTGGTTGATACACCGAAACTT
100576_CS14     ATTTACACAAATGACCCTTCAAAAGGTTTAGATGTTCGACTGGTTGATACACCGAAACTT
503825_CS14     ATTTACACAAATGACCCTTCAAAAGGTTTAGATGTTCGACTGGTTGATACACCGAAACTT
200023_CS14     ATTTACACAAATGACCCTTCAAAAGGTTTAGATGTTCGACTGGTTGATACACCGAAACTT
300316_CS14     ATTTACACAAATGACCCTTCAAAAGGTTTAGATGTTCGACTGGTTGATACACCGAAACTT
700434_CS14     ATTTACACAAATGACCCTTCAAAAGGTTTAGATGTTCGACTGGTTGATACACCGAAACTT
```

```
602762_CS14      ATTTACACAAATGACCCTTCAAAAGGTTTAGATGTTCGACTGGTTGATACACCGAAACTT
400599_CS14      ATTTACACAAATGACCCTTCAAAAGGTTTAGATGTTCGACTGGTTGATACACCGAAACTT
                 ************************************************************

AY283611.1       ACAAATATTTTGCAACCGACATCTACCATTCCTCTTACTGTCTCATGGGCAGGGAGGACA
100576_CS14      ACAAATATTTTGCAACCGACATCTACCATTCCTCTTACTGTCTCATGGGCAGGGAGGACA
503825_CS14      ACAAATATTTTGCAACCGACATCTACCATTCCTCTTACTGTCTCATGGGCAGGGAGGACA
200023_CS14      ACAAATATTTTGCAACCGACATCTACCATTCCTCTTACTGTCTCATGGGCAGGGAGGACA
300316_CS14      ACAAATATTTTGCAACCGACATCTACCATTCCTCTTACTGTCTCATGGGCAGGGAGGACA
700434_CS14      ACAAATATTTTGCAACCGACATCTACCATTCCTCTTACTGTCTCATGGGCAGGGAGGACA
602762_CS14      ACAAATATTTTGCAACCGACATCTACCATTCCTCTTACTGTCTCATGGGCAGGGAGGACA
400599_CS14      ACAAATATTTTGCAACCGACATCTACCATTCCTCTTACTGTCTCATGGGCAGGGAGGACA
                 ************************************************************

AY283611.1       TTAAGTACAAGTGCTCAGAAGATCGCAGTTGGCGATCTGGGTTTTGGTTCCACCGGAACG
100576_CS14      TTAAGTACAAGTGCTCAGAAGATCGCAGTTGGCGATCTGGGTTTTGGTTCCACCGGAACG
503825_CS14      TTAAGTACAAGTGCTCAGAAGATCGCAGTTGGCGATCTGGGTTTTGGTTCCACCGGAACG
200023_CS14      TTAAGTACAAGTGCTCAGAAGATCGCAGTTGGCGATCTGGGTTTTGGTTCCACCGGAACG
300316_CS14      TTAAGTACAAGTGCTCAGAAGATCGCAGTTGGCGATCTGGGTTTTGGTTCCACCGGAACG
700434_CS14      TTAAGTACAAGTGCTCAGAAGATCGCAGTTGGCGATCTGGGTTTTGGTTCCACCGGAACG
602762_CS14      TTAAGTACAAGTGCTCAGAAGATCGCAGTTGGCGATCTGGGTTTTGGTTCCACCGGAACG
400599_CS14      TTAAGTACAAGTGCTCAGAAGATCGCAGTTGGCGATCTGGGTTTTGGTTCCACCGGAACG
                 ************************************************************

AY283611.1       GCAGGTGTTTCGAATAGTAAAGAATTAGTAATTGGAGCAACTACATCCGGAACTGCACCA
100576_CS14      GCAGGTGTTTCGAATAGTAAAGAATTAGTAATTGGAGCAACTACATCCGGAACTGCACCA
503825_CS14      GCAGGTGTTTCGAATAGTAAAGAATTAGTAATTGGAGCAACTACATCCGGAACTGCACCA
200023_CS14      GCAGGTGTTTCGAATAGTAAAGAATTAGTAATTGGAGCAACTACATCCGGAACTGCACCA
300316_CS14      GCAGGTGTTTCGAATAGTAAAGAATTAGTAATTGGAGCAACTACATCCGGAACTGCACCA
700434_CS14      GCAGGTGTTTCGAATAGTAAAGAATTAGTAATTGGAGCAACTACATCCGGAACTGCACCA
602762_CS14      GCAGGTGTTTCGAATAGTAAAGAATTAGTAATTGGAGCAACTACATCCGGAACTGCACCA
400599_CS14      GCAGGTGTTTCGAATAGTAAAGAATTAGTAATTGGAGCAACTACATCCGGAACTGCACCA
                 ************************************************************

AY283611.1       AGTGCAGGTAAGTATCAAGGCGTCGTTTCCATTGTAATGACTCAATCGACAAACTAAGGA
100576_CS14      AGTGCAGGTAAGTATCAAGGCGTCGTTTCCATTGTAATGACTCAATCGACAAACTAAGGA
503825_CS14      AGTGCAGGTAAGTATCAAGGCGTCGTTTCCATTGTAATGACTCAATCGACAAACTAAGGA
200023_CS14      AGTGCAGGTAAGTATCAAGGCGTCGTTTCCATTGTAATGACTCAATCGACAAACTAAGGA
300316_CS14      AGTGCAGGTAAGTATCAAGGCGTCGTTTCCATTGTAATGACTCAATCGACAAACTAAGGA
700434_CS14      AGTGCAGGTAAGTATCAAGGCGTCGTTTCCATTGTAATGACTCAATCGACAAACTAAGGA
602762_CS14      AGTGCAGGTAAGTATCAAGGCGTCGTTTCCATTGTAATGACTCAATCGACAAACTAAGGA
400599_CS14      AGTGCAGGTAAGTATCAAGGCGTCGTTTCCATTGTAATGACTCAATCGACAAACTAAGGA
                 ************************************************************

AY283611.1       GTTCTAATGAAATTAAAAAAAACTATTGGCGCAATGGCTCTGAGCACAATGTTTGTAGCG
100576_CS14      GTTCTAATGAAATTAAAAAAAACTATTGGCGCAATGGCTCTGAGCACAATGTTTGTAGCG
503825_CS14      GTTCTAATGAAATTAAAAAAAACTATTGGCGCAATGGCTCTGAGCACAATGTTTGTAGCG
200023_CS14      GTTCTAATGAAATTAAAAAAAACTATTGGCGCAATGGCTCTGAGCACAATGTTTGTAGCG
300316_CS14      GTTCTAATGAAATTAAAAAAAACTATTGGCGCAATGGCTCTGAGCACAATGTTTGTAGCG
700434_CS14      GTTCTAATGAAATTAAAAAAAACTATTGGCGCAATGGCTCTGAGCACAATGTTTGTAGCG
602762_CS14      GTTCTAATGAAATTAAAAAAAACTATTGGCGCAATGGCTCTGAGCACAATGTTTGTAGCG
400599_CS14      GTTCTAATGAAATTAAAAAAAACTATTGGCGCAATGGCTCTGAGCACAATGTTTGTAGCG
                 ************************************************************

AY283611.1       GTGAGTGCTTCAGCAGTAGAGAAAAATATTACTGTGACAGCCAGTGTTGATCCTACTATT
100576_CS14      GTGAGTGCTTCAGCAGTAGAGAAAAATATTACTGTGACAGCCAGTGTTGATCCTACTATT
503825_CS14      GTGAGTGCTTCAGCAGTAGAGAAAAATATTACTGTGACAGCCAGTGTTGATCCTACTATT
200023_CS14      GTGAGTGCTTCAGCAGTAGAGAAAAATATTACTGTGACAGCCAGTGTTGATCCTACTATT
300316_CS14      GTGAGTGCTTCAGCAGTAGAGAAAAATATTACTGTGACAGCCAGTGTTGATCCTACTATT
700434_CS14      GTGAGTGCTTCAGCAGTAGAGAAAAATATTACTGTGACAGCCAGTGTTGATCCTACTATT
602762_CS14      GTGAGTGCTTCAGCAGTAGAGAAAAATATTACTGTGACAGCCAGTGTTGATCCTACTATT
400599_CS14      GTGAGTGCTTCAGCAGTAGAGAAAAATATTACTGTGACAGCCAGTGTTGATCCTACTATT
                 ************************************************************

AY283611.1       GATATTCTTCAAGCAAATGGTTCTGCGCTACCGACAGCTGTAGATTTAACTTATCTACCT
```

```
100576_CS14     GATATTCTTCAAGCAAATGGTTCTGCGCTACCGACAGCTGTAGATTTAACTTATCTACCT
503825_CS14     GATATTCTTCAAGCAAATGGTTCTGCGCTACCGACAGCTGTAGATTTAACTTATCTACCT
200023_CS14     GATATTCTTCAAGCAAATGGTTCTGCGCTACCGACAGCTGTAGATTTAACTTATCTACCT
300316_CS14     GATATTCTTCAAGCAAATGGTTCTGCGCTACCGACAGCTGTAGATTTAACTTATCTACCT
700434_CS14     GATATTCTTCAAGCAAATGGTTCTGCGCTACCGACAGCTGTAGATTTAACTTATCTACCT
602762_CS14     GATATTCTTCAAGCAAATGGTTCTGCGCTACCGACAGCTGTAGATTTAACTTATCTACCT
400599_CS14     GATATTCTTCAAGCAAATGGTTCTGCGCTACCGACAGCTGTAGATTTAACTTATCTACCT
                ************************************************************

AY283611.1      GGTGCAAAAACTTTTGAAAATTACAGTGTTCTAACCCAGATTTACACAAATGACCCTTCA
100576_CS14     GGTGCAAAAACTTTTGAAAATTACAGTGTTCTAACCCAGATTTACACAAATGACCCTTCA
503825_CS14     GGTGCAAAAACTTTTGAAAATTACAGTGTTCTAACCCAGATTTACACAAATGACCCTTCA
200023_CS14     GGTGCAAAAACTTTTGAAAATTACAGTGTTCTAACCCAGATTTACACAAATGACCCTTCA
300316_CS14     GGTGCAAAAACTTTTGAAAATTACAGTGTTCTAACCCAGATTTACACAAATGACCCTTCA
700434_CS14     GGTGCAAAAACTTTTGAAAATTACAGTGTTCTAACCCAGATTTACACAAATGACCCTTCA
602762_CS14     GGTGCAAAAACTTTTGAAAATTACAGTGTTCTAACCCAGATTTACACAAATGACCCTTCA
400599_CS14     GGTGCAAAAACTTTTGAAAATTACAGTGTTCTAACCCAGATTTACACAAATGACCCTTCA
                ************************************************************

AY283611.1      AAAGGTTTAGATGTTCGACTGGTTGATACACCGAAACTTACAAATATTTTGCAACCGACA
100576_CS14     AAAGGTTTAGATGTTCGACTGGTTGATACACCGAAACTTACAAATATTTTGCAACCGACA
503825_CS14     AAAGGTTTAGATGTTCGACTGGTTGATACACCGAAACTTACAAATATTTTGCAACCGACA
200023_CS14     AAAGGTTTAGATGTTCGACTGGTTGATACACCGAAACTTACAAATATTTTGCAACCGACA
300316_CS14     AAAGGTTTAGATGTTCGACTGGTTGATACACCGAAACTTACAAATATTTTGCAACCGACA
700434_CS14     AAAGGTTTAGATGTTCGACTGGTTGATACACCGAAACTTACAAATATTTTGCAACCGACA
602762_CS14     AAAGGTTTAGATGTTCGACTGGTTGATACACCGAAACTTACAAATATTTTGCAACCGACA
400599_CS14     AAAGGTTTAGATGTTCGACTGGTTGATACACCGAAACTTACAAATATTTTGCAACCGACA
                ************************************************************

AY283611.1      TCTACCATTCCTCTTACTGTCTCATGGGCAGGGAAGACATTAAGTACAAGTGCTCAGAAG
100576_CS14     TCTACCATTCCTCTTACTGTCTCATGGGCAGGGAAGACATTAAGTACAAGTGCTCAGAAG
503825_CS14     TCTACCATTCCTCTTACTGTCTCATGGGCAGGGAAGACATTAAGTACAAGTGCTCAGAAG
200023_CS14     TCTACCATTCCTCTTACTGTCTCATGGGCAGGGAAGACATTAAGTACAAGTGCTCAGAAG
300316_CS14     TCTACCATTCCTCTTACTGTCTCATGGGCAGGGAAGACATTAAGTACAAGTGCTCAGAAG
700434_CS14     TCTACCATTCCTCTTACTGTCTCATGGGCAGGGAAGACATTAAGTACAAGTGCTCAGAAG
602762_CS14     TCTACCATTCCTCTTACTGTCTCATGGGCAGGGAAGACATTAAGTACAAGTGCTCAGAAG
400599_CS14     TCTACCATTCCTCTTACTGTCTCATGGGCAGGGAAGACATTAAGTACAAGTGCTCAGAAG
                ************************************************************

AY283611.1      ATTGCAGTTGGCGATCTGGGTTTTGGTTCCACCGGAACGGCAGGTGTTTCGAATAGTAAA
100576_CS14     ATTGCAGTTGGCGATCTGGGTTTTGGTTCCACCGGAACGGCAGGTGTTTCGAATAGTAAA
503825_CS14     ATTGCAGTTGGCGATCTGGGTTTTGGTTCCACCGGAACGGCAGGTGTTTCGAATAGTAAA
200023_CS14     ATTGCAGTTGGCGATCTGGGTTTTGGTTCCACCGGAACGGCAGGTGTTTCGAATAGTAAA
300316_CS14     ATTGCAGTTGGCGATCTGGGTTTTGGTTCCACCGGAACGGCAGGTGTTTCGAATAGTAAA
700434_CS14     ATTGCAGTTGGCGATCTGGGTTTTGGTTCCACCGGAACGGCAGGTGTTTCGAATAGTAAA
602762_CS14     ATTGCAGTTGGCGATCTGGGTTTTGGTTCCACCGGAACGGCAGGTGTTTCGAATAGTAAA
400599_CS14     ATTGCAGTTGGCGATCTGGGTTTTGGTTCCACCGGAACGGCAGGTGTTTCGAATAGTAAA
                ************************************************************

AY283611.1      GAATTAGTAATTGGAGCAACTACATCCGGAACTGCACCAAGTGCAGGTAAGTATCAAGGC
100576_CS14     GAATTAGTAATTGGAGCAACTACATCCGGAACTGCACCAAGTGCAGGTAAGTATCAAGGC
503825_CS14     GAATTAGTAATTGGAGCAACTACATCCGGAACTGCACCAAGTGCAGGTAAGTATCAAGGC
200023_CS14     GAATTAGTAATTGGAGCAACTACATCCGGAACTGCACCAAGTGCAGGTAAGTATCAAGGC
300316_CS14     GAATTAGTAATTGGAGCAACTACATCCGGAACTGCACCAAGTGCAGGTAAGTATCAAGGC
700434_CS14     GAATTAGTAATTGGAGCAACTACATCCGGAACTGCACCAAGTGCAGGTAAGTATCAAGGC
602762_CS14     GAATTAGTAATTGGAGCAACTACATCCGGAACTGCACCAAGTGCAGGTAAGTATCAAGGC
400599_CS14     GAATTAGTAATTGGAGCAACTACATCCGGAACTGCACCAAGTGCAGGTAAGTATCAAGGC
                ************************************************************

AY283611.1      GTCGTTTCCATTGTAATGACTCAATCGACAGACACAGCCGCGCCTGTTCCTTAATAAAAT
100576_CS14     GTCGTTTCCATTGTAATGACTCAATCGACAGACACAGCCGCGCCTGTTCCTTAATAAAAT
503825_CS14     GTCGTTTCCATTGTAATGACTCAATCGACAGACACAGCCGCGCCTGTTCCTTAATAAAAT
200023_CS14     GTCGTTTCCATTGTAATGACTCAATCGACAGACACAGCCGCGCCTGTTCCTTAATAAAAT
300316_CS14     GTCGTTTCCATTGTAATGACTCAATCGACAGACACAGCCGCGCCTGTTCCTTAATAAAAT
700434_CS14     GTCGTTTCCATTGTAATGACTCAATCGACAGACACAGCCGCGCCTGTTCCTTAATAAAAT
```

```
602762_CS14     GTCGTTTCCATTGTAATGACTCAATCGACAGACACAGCCGCGCCTGTTCCTTAATAAAAT
400599_CS14     GTCGTTTCCATTGTAATGACTCAATCGACAGACACAGCCGCGCCTGTTCCTTAATAAAAT
                ************************************************************

AY283611.1      GTAATAACAACAAGAGCAGCTATCATTCGTAGCTGCTCATTCCTTAATGATGAAAACCAT
100576_CS14     GTAATAACAACAAGAGCAGCTATCATTCGTAGCTGCTCATTCCTTAATGATGAAAACCAT
503825_CS14     GTAATAACAACAAGAGCAGCTATCATTCGTAGCTGCTCATTCCTTAATGATGAAAACCAT
200023_CS14     GTAATAACAACAAGAGCAGCTATCATTCGTAGCTGCTCATTCCTTAATGATGAAAACCAT
300316_CS14     GTAATAACAACAAGAGCAGCTATCATTCGTAGCTGCTCATTCCTTAATGATGAAAACCAT
700434_CS14     GTAATAACAACAAGAGCAGCTATCATTCGTAGCTGCTCATTCCTTAATGATGAAAACCAT
602762_CS14     GTAATAACAACAAGAGCAGCTATCATTCGTAGCTGCTCATTCCTTAATGATGAAAACCAT
400599_CS14     GTAATAACAACAAGAGCAGCTATCATTCGTAGCTGCTCATTCCTTAATGATGAAAACCAT
                ************************************************************

AY283611.1      TATGATTTATAAAAAATTCAGAAAAAGAAAAATATCTTTATTTATTTCTATGTTCTTTAT
100576_CS14     TATGATTTATAAAAAATTCAGAAAAAGAAAAATATCTTTATTTATTTCTATGTTCTTTAT
503825_CS14     TATGATTTATAAAAAATTCAGAAAAAGAAAAATATCTTTATTTATTTCTATGTTCTTTAT
200023_CS14     TATGATTTATAAAAAATTCAGAAAAAGAAAAATATCTTTATTTATTTCTATGTTCTTTAT
300316_CS14     TATGATTTATAAAAAATTCAGAAAAAGAAAAATATCTTTATTTATTTCTATGTTCTTTAT
700434_CS14     TATGATTTATAAAAAATTCAGAAAAAGAAAAATATCTTTATTTATTTCTATGTTCTTTAT
602762_CS14     TATGATTTATAAAAAATTCAGAAAAAGAAAAATATCTTTATTTATTTCTATGTTCTTTAT
400599_CS14     TATGATTTATAAAAAATTCAGAAAAAGAAAAATATCTTTATTTATTTCTATGTTCTTTAT
                ************************************************************

AY283611.1      ATCATGCTCAGTTTTTGCAGATGATATACCCGACTCTTTCCGTGATTTATGGGGAGAACA
100576_CS14     ATCATGCTCAGTTTTTGCAGATGATATACCCGACTCTTTCCGTGATTTATGGGGAGAACA
503825_CS14     ATCATGCTCAGTTTTTGCAGATGATATACCCGACTCTTTCCGTGATTTATGGGGAGAACA
200023_CS14     ATCATGCTCAGTTTTTGCAGATGATATACCCGACTCTTTCCGTGATTTATGGGGAGAACA
300316_CS14     ATCATGCTCAGTTTTTGCAGATGATATACCCGACTCTTTCCGTGATTTATGGGGAGAACA
700434_CS14     ATCATGCTCAGTTTTTGCAGATGATATACCCGACTCTTTCCGTGATTTATGGGGAGAACA
602762_CS14     ATCATGCTCAGTTTTTGCAGATGATATACCCGACTCTTTCCGTGATTTATGGGGAGAACA
400599_CS14     ATCATGCTCAGTTTTTGCAGATGATATACCCGACTCTTTCCGTGATTTATGGGGAGAACA
                ************************************************************

AY283611.1      AGATGAATTTTATGAAGTAAAACTATATGGGCAAACTCTAGGAATACATCGAATTAAAAC
100576_CS14     AGATGAATTTTATGAAGTAAAACTATATGGGCAAACTCTAGGAATACATCGAATTAAAAC
503825_CS14     AGATGAATTTTATGAAGTAAAACTATATGGGCAAACTCTAGGAATACATCGAATTAAAAC
200023_CS14     AGATGAATTTTATGAAGTAAAACTATATGGGCAAACTCTAGGAATACATCGAATTAAAAC
300316_CS14     AGATGAATTTTATGAAGTAAAACTATATGGGCAAACTCTAGGAATACATCGAATTAAAAC
700434_CS14     AGATGAATTTTATGAAGTAAAACTATATGGGCAAACTCTAGGAATACATCGAATTAAAAC
602762_CS14     AGATGAATTTTATGAAGTAAAACTATATGGGCAAACTCTAGGAATACATCGAATTAAAAC
400599_CS14     AGATGAATTTTATGAAGTAAAACTATATGGGCAAACTCTAGGAATACATCGAATTAAAAC
                ************************************************************

AY283611.1      AACCCCAACACATATTAAGTTTTATTCACCCGAAAGCATTTTAGATAAAATAAATTTAAA
100576_CS14     AACCCCAACACATATTAAGTTTTATTCACCCGAAAGCATTTTAGATAAAATAAATTTAAA
503825_CS14     AACCCCAACACATATTAAGTTTTATTCACCCGAAAGCATTTTAGATAAAATAAATTTAAA
200023_CS14     AACCCCAACACATATTAAGTTTTATTCACCCGAAAGCATTTTAGATAAAATAAATTTAAA
300316_CS14     AACCCCAACACATATTAAGTTTTATTCACCCGAAAGCATTTTAGATAAAATAAATTTAAA
700434_CS14     AACCCCAACACATATTAAGTTTTATTCACCCGAAAGCATTTTAGATAAAATAAATTTAAA
602762_CS14     AACCCCAACACATATTAAGTTTTATTCACCCGAAAGCATTTTAGATAAAATAAATTTAAA
400599_CS14     AACCCCAACACATATTAAGTTTTATTCACCCGAAAGCATTTTAGATAAAATAAATTTAAA
                ************************************************************

AY283611.1      AAAAGAAAAGGAAAAGGAATTGAGTGTTCTTTTTACTAATTCTTTTTCAAGAAATGGCAA
100576_CS14     AAAAGAAAAGGAAAAGGAATTGAGTGTTCTTTTTACTAATTCTTTTTCAAGAAATGGCAA
503825_CS14     AAAAGAAAAGGAAAAGGAATTGAGTGTTCTTTTTACTAATTCTTTTTCAAGAAATGGCAA
200023_CS14     AAAAGAAAAGGAAAAGGAATTGAGTGTTCTTTTTACTAATTCTTTTTCAAGAAATGGCAA
300316_CS14     AAAAGAAAAGGAAAAGGAATTGAGTGTTCTTTTTACTAATTCTTTTTCAAGAAATGGCAA
700434_CS14     AAAAGAAAAGGAAAAGGAATTGAGTGTTCTTTTTACTAATTCTTTTTCAAGAAATGGCAA
602762_CS14     AAAAGAAAAGGAAAAGGAATTGAGTGTTCTTTTTACTAATTCTTTTTCAAGAAATGGCAA
400599_CS14     AAAAGAAAAGGAAAAGGAATTGAGTGTTCTTTTTACTAATTCTTTTTCAAGAAATGGCAA
                ************************************************************

AY283611.1      TATGAGTTGTCAGGGTAACGCTGCTATACAGTATAACTGCAATTACATTAAAACAAAATC
```

```
100576_CS14     TATGAGTTGTCAGGGTAACGCTGCTATACAGTATAACTGCAATTACATTAAAACAAAATC
503825_CS14     TATGAGTTGTCAGGGTAACGCTGCTATACAGTATAACTGCAATTACATTAAAACAAAATC
200023_CS14     TATGAGTTGTCAGGGTAACGCTGCTATACAGTATAACTGCAATTACATTAAAACAAAATC
300316_CS14     TATGAGTTGTCAGGGTAACGCTGCTATACAGTATAACTGCAATTACATTAAAACAAAATC
700434_CS14     TATGAGTTGTCAGGGTAACGCTGCTATACAGTATAACTGCAATTACATTAAAACAAAATC
602762_CS14     TATGAGTTGTCAGGGTAACGCTGCTATACAGTATAACTGCAATTACATTAAAACAAAATC
400599_CS14     TATGAGTTGTCAGGGTAACGCTGCTATACAGTATAACTGCAATTACATTAAAACAAAATC
                ************************************************************

AY283611.1      AGTAGATGTCATCGTTGATGATGTTGATAATGTTGTTAACCTTTTTATAGGTAATGAATT
100576_CS14     AGTAGATGTCATCGTTGATGATGTTGATAATGTTGTTAACCTTTTTATAGGTAATGAATT
503825_CS14     AGTAGATGTCATCGTTGATGATGTTGATAATGTTGTTAACCTTTTTATAGGTAATGAATT
200023_CS14     AGTAGATGTCATCGTTGATGATGTTGATAATGTTGTTAACCTTTTTATAGGTAATGAATT
300316_CS14     AGTAGATGTCATCGTTGATGATGTTGATAATGTTGTTAACCTTTTTATAGGTAATGAATT
700434_CS14     AGTAGATGTCATCGTTGATGATGTTGATAATGTTGTTAACCTTTTTATAGGTAATGAATT
602762_CS14     AGTAGATGTCATCGTTGATGATGTTGATAATGTTGTTAACCTTTTTATAGGTAATGAATT
400599_CS14     AGTAGATGTCATCGTTGATGATGTTGATAATGTTGTTAACCTTTTTATAGGTAATGAATT
                ************************************************************

AY283611.1      TCTGGATTCTGAAGCACACAGTGATGAATACCATCAATTATCACGAAATGTAAAAAAAGC
100576_CS14     TCTGGATTCTGAAGCACACAGTGATGAATACCATCAATTATCACGAAATGTAAAAAAAGC
503825_CS14     TCTGGATTCTGAAGCACACAGTGATGAATACCATCAATTATCACGAAATGTAAAAAAAGC
200023_CS14     TCTGGATTCTGAAGCACACAGTGATGAATACCATCAATTATCACGAAATGTAAAAAAAGC
300316_CS14     TCTGGATTCTGAAGCACACAGTGATGAATACCATCAATTATCACGAAATGTAAAAAAAGC
700434_CS14     TCTGGATTCTGAAGCACACAGTGATGAATACCATCAATTATCACGAAATGTAAAAAAAGC
602762_CS14     TCTGGATTCTGAAGCACACAGTGATGAATACCATCAATTATCACGAAATGTAAAAAAAGC
400599_CS14     TCTGGATTCTGAAGCACACAGTGATGAATACCATCAATTATCACGAAATGTAAAAAAAGC
                ************************************************************

AY283611.1      TTTTATACAAAGCCAGACAATTAATGTCTCAGATTCTGGGAAGTATAAAAATCTGTCTAT
100576_CS14     TTTTATACAAAGCCAGACAATTAATGTCTCAGATTCTGGGAAGTATAAAAATCTGTCTAT
503825_CS14     TTTTATACAAAGCCAGACAATTAATGTCTCAGATTCTGGGAAGTATAAAAATCTGTCTAT
200023_CS14     TTTTATACAAAGCCAGACAATTAATGTCTCAGATTCTGGGAAGTATAAAAATCTGTCTAT
300316_CS14     TTTTATACAAAGCCAGACAATTAATGTCTCAGATTCTGGGAAGTATAAAAATCTGTCTAT
700434_CS14     TTTTATACAAAGCCAGACAATTAATGTCTCAGATTCTGGGAAGTATAAAAATCTGTCTAT
602762_CS14     TTTTATACAAAGCCAGACAATTAATGTCTCAGATTCTGGGAAGTATAAAAATCTGTCTAT
400599_CS14     TTTTATACAAAGCCAGACAATTAATGTCTCAGATTCTGGGAAGTATAAAAATCTGTCTAT
                ************************************************************

AY283611.1      TTCAGGGAATAGTGCACTGGGTATTATAGGTACAAGTTATGCTGTCTTAAATTGGTGGAT
100576_CS14     TTCAGGGAATAGTGCACTGGGTATTATAGGTACAAGTTATGCTGTCTTAAATTGGTGGAT
503825_CS14     TTCAGGGAATAGTGCACTGGGTATTATAGGTACAAGTTATGCTGTCTTAAATTGGTGGAT
200023_CS14     TTCAGGGAATAGTGCACTGGGTATTATAGGTACAAGTTATGCTGTCTTAAATTGGTGGAT
300316_CS14     TTCAGGGAATAGTGCACTGGGTATTATAGGTACAAGTTATGCTGTCTTAAATTGGTGGAT
700434_CS14     TTCAGGGAATAGTGCACTGGGTATTATAGGTACAAGTTATGCTGTCTTAAATTGGTGGAT
602762_CS14     TTCAGGGAATAGTGCACTGGGTATTATAGGTACAAGTTATGCTGTCTTAAATTGGTGGAT
400599_CS14     TTCAGGGAATAGTGCACTGGGTATTATAGGTACAAGTTATGCTGTCTTAAATTGGTGGAT
                ************************************************************

AY283611.1      GAATTACAATAAATCTAATGGTTACAGCAACAACGAAAAAACAATCAATAGTTTATACTT
100576_CS14     GAATTACAATAAATCTAATGGTTACAGCAACAACGAAAAAACAATCAATAGTTTATACTT
503825_CS14     GAATTACAATAAATCTAATGGTTACAGCAACAACGAAAAAACAATCAATAGTTTATACTT
200023_CS14     GAATTACAATAAATCTAATGGTTACAGCAACAACGAAAAAACAATCAATAGTTTATACTT
300316_CS14     GAATTACAATAAATCTAATGGTTACAGCAACAACGAAAAAACAATCAATAGTTTATACTT
700434_CS14     GAATTACAATAAATCTAATGGTTACAGCAACAACGAAAAAACAATCAATAGTTTATACTT
602762_CS14     GAATTACAATAAATCTAATGGTTACAGCAACAACGAAAAAACAATCAATAGTTTATACTT
400599_CS14     GAATTACAATAAATCTAATGGTTACAGCAACAACGAAAAAACAATCAATAGTTTATACTT
                ************************************************************

AY283611.1      TAGACATGATTTAGATAAGAGATATTATTATCAATTTGGACGAATGGATCGTACAGATTT
100576_CS14     TAGACATGATTTAGATAAGAGATATTATTATCAATTTGGACGAATGGATCGTACAGATTT
503825_CS14     TAGACATGATTTAGATAAGAGATATTATTATCAATTTGGACGAATGGATCGTACAGATTT
200023_CS14     TAGACATGATTTAGATAAGAGATATTATTATCAATTTGGACGAATGGATCGTACAGATTT
300316_CS14     TAGACATGATTTAGATAAGAGATATTATTATCAATTTGGACGAATGGATCGTACAGATTT
700434_CS14     TAGACATGATTTAGATAAGAGATATTATTATCAATTTGGACGAATGGATCGTACAGATTT
```

```
602762_CS14     TAGACATGATTTAGATAAGAGATATTATTATCAATTTGGACGAATGGATCGTACAGATTT
400599_CS14     TAGACATGATTTAGATAAGAGATATTATTATCAATTTGGACGAATGGATCGTACAGATTT
                ************************************************************

AY283611.1      GTCACAAAGTATTAGCGGGAGCTTTAATTTTAACTTACTTCCTTTACCCGATATTGATGG
100576_CS14     GTCACAAAGTATTAGCGGGAGCTTTAATTTTAACTTACTTCCTTTACCCGATATTGATGG
503825_CS14     GTCACAAAGTATTAGCGGGAGCTTTAATTTTAACTTACTTCCTTTACCCGATATTGATGG
200023_CS14     GTCACAAAGTATTAGCGGGAGCTTTAATTTTAACTTACTTCCTTTACCCGATATTGATGG
300316_CS14     GTCACAAAGTATTAGCGGGAGCTTTAATTTTAACTTACTTCCTTTACCCGATATTGATGG
700434_CS14     GTCACAAAGTATTAGCGGGAGCTTTAATTTTAACTTACTTCCTTTACCCGATATTGATGG
602762_CS14     GTCACAAAGTATTAGCGGGAGCTTTAATTTTAACTTACTTCCTTTACCCGATATTGATGG
400599_CS14     GTCACAAAGTATTAGCGGGAGCTTTAATTTTAACTTACTTCCTTTACCCGATATTGATGG
                ************************************************************

AY283611.1      CATACGGACAGGAACCACACAATCTTATATCAAAAATACAGATAAGTTTATCGCATCCCC
100576_CS14     CATACGGACAGGAACCACACAATCTTATATCAAAAATACAGATAAGTTTATCGCATCCCC
503825_CS14     CATACGGACAGGAACCACACAATCTTATATCAAAAATACAGATAAGTTTATCGCATCCCC
200023_CS14     CATACGGACAGGAACCACACAATCTTATATCAAAAATACAGATAAGTTTATCGCATCCCC
300316_CS14     CATACGGACAGGAACCACACAATCTTATATCAAAAATACAGATAAGTTTATCGCATCCCC
700434_CS14     CATACGGACAGGAACCACACAATCTTATATCAAAAATACAGATAAGTTTATCGCATCCCC
602762_CS14     CATACGGACAGGAACCACACAATCTTATATCAAAAATACAGATAAGTTTATCGCATCCCC
400599_CS14     CATACGGACAGGAACCACACAATCTTATATCAAAAATACAGATAAGTTTATCGCATCCCC
                ************************************************************

AY283611.1      TGTAACTGTTATGTTAACTAATTTTTCCAGAGTGGAAGCTTTTCGCAATGATCAATTATT
100576_CS14     TGTAACTGTTATGTTAACTAATTTTTCCAGAGTGGAAGCTTTTCGCAATGATCAATTATT
503825_CS14     TGTAACTGTTATGTTAACTAATTTTTCCAGAGTGGAAGCTTTTCGCAATGATCAATTATT
200023_CS14     TGTAACTGTTATGTTAACTAATTTTTCCAGAGTGGAAGCTTTTCGCAATGATCAATTATT
300316_CS14     TGTAACTGTTATGTTAACTAATTTTTCCAGAGTGGAAGCTTTTCGCAATGATCAATTATT
700434_CS14     TGTAACTGTTATGTTAACTAATTTTTCCAGAGTGGAAGCTTTTCGCAATGATCAATTATT
602762_CS14     TGTAACTGTTATGTTAACTAATTTTTCCAGAGTGGAAGCTTTTCGCAATGATCAATTATT
400599_CS14     TGTAACTGTTATGTTAACTAATTTTTCCAGAGTGGAAGCTTTTCGCAATGATCAATTATT
                ************************************************************

AY283611.1      GGGCGTATGGTATTTAGATTCTGGAGTAAATGAATTAGATACAGCTCGTTTACCTTATGG
100576_CS14     GGGCGTATGGTATTTAGATTCTGGAGTAAATGAATTAGATACAGCTCGTTTACCTTATGG
503825_CS14     GGGCGTATGGTATTTAGATTCTGGAGTAAATGAATTAGATACAGCTCGTTTACCTTATGG
200023_CS14     GGGCGTATGGTATTTAGATTCTGGAGTAAATGAATTAGATACAGCTCGTTTACCTTATGG
300316_CS14     GGGCGTATGGTATTTAGATTCTGGAGTAAATGAATTAGATACAGCTCGTTTACCTTATGG
700434_CS14     GGGCGTATGGTATTTAGATTCTGGAGTAAATGAATTAGATACAGCTCGTTTACCTTATGG
602762_CS14     GGGCGTATGGTATTTAGATTCTGGAGTAAATGAATTAGATACAGCTCGTTTACCTTATGG
400599_CS14     GGGCGTATGGTATTTAGATTCTGGAGTAAATGAATTAGATACAGCTCGTTTACCTTATGG
                ************************************************************

AY283611.1      TAGTTACGATCTTAAATTGAAGATTTTTGAAAAAACTCAGTTAGTTCGTGAAGAAATAAT
100576_CS14     TAGTTACGATCTTAAATTGAAGATTTTTGAAAAAACTCAGTTAGTTCGTGAAGAAATAAT
503825_CS14     TAGTTACGATCTTAAATTGAAGATTTTTGAAAAAACTCAGTTAGTTCGTGAAGAAATAAT
200023_CS14     TAGTTACGATCTTAAATTGAAGATTTTTGAAAAAACTCAGTTAGTTCGTGAAGAAATAAT
300316_CS14     TAGTTACGATCTTAAATTGAAGATTTTTGAAAAAACTCAGTTAGTTCGTGAAGAAATAAT
700434_CS14     TAGTTACGATCTTAAATTGAAGATTTTTGAAAAAACTCAGTTAGTTCGTGAAGAAATAAT
602762_CS14     TAGTTACGATCTTAAATTGAAGATTTTTGAAAAAACTCAGTTAGTTCGTGAAGAAATAAT
400599_CS14     TAGTTACGATCTTAAATTGAAGATTTTTGAAAAAACTCAGTTAGTTCGTGAAGAAATAAT
                ************************************************************

AY283611.1      TCCTTTTAATAAAGGGAGAAGTTCTATTGGTGATATGCAATGGGACGTTTTCGTTCAGGG
100576_CS14     TCCTTTTAATAAAGGGAGAAGTTCTATTGGTGATATGCAATGGGACGTTTTCGTTCAGGG
503825_CS14     TCCTTTTAATAAAGGGAGAAGTTCTATTGGTGATATGCAATGGGACGTTTTCGTTCAGGG
200023_CS14     TCCTTTTAATAAAGGGAGAAGTTCTATTGGTGATATGCAATGGGACGTTTTCGTTCAGGG
300316_CS14     TCCTTTTAATAAAGGGAGAAGTTCTATTGGTGATATGCAATGGGACGTTTTCGTTCAGGG
700434_CS14     TCCTTTTAATAAAGGGAGAAGTTCTATTGGTGATATGCAATGGGACGTTTTCGTTCAGGG
602762_CS14     TCCTTTTAATAAAGGGAGAAGTTCTATTGGTGATATGCAATGGGACGTTTTCGTTCAGGG
400599_CS14     TCCTTTTAATAAAGGGAGAAGTTCTATTGGTGATATGCAATGGGACGTTTTCGTTCAGGG
                ************************************************************

AY283611.1      AGGGAATATTGTTAATGACAAGGGTCGTTACATAGAAAAACAATATAATCATAAGTCATC
```

```
100576_CS14     AGGGAATATTGTTAATGACAAGGGTCGTTACATAGAAAAACAATATAATCATAAGTCATC
503825_CS14     AGGGAATATTGTTAATGACAAGGGTCGTTACATAGAAAAACAATATAATCATAAGTCATC
200023_CS14     AGGGAATATTGTTAATGACAAGGGTCGTTACATAGAAAAACAATATAATCATAAGTCATC
300316_CS14     AGGGAATATTGTTAATGACAAGGGTCGTTACATAGAAAAACAATATAATCATAAGTCATC
700434_CS14     AGGGAATATTGTTAATGACAAGGGTCGTTACATAGAAAAACAATATAATCATAAGTCATC
602762_CS14     AGGGAATATTGTTAATGACAAGGGTCGTTACATAGAAAAACAATATAATCATAAGTCATC
400599_CS14     AGGGAATATTGTTAATGACAAGGGTCGTTACATAGAAAAACAATATAATCATAAGTCATC
                ************************************************************

AY283611.1      AGTTAATGCTGGGCTACGTTTACCAATTACGAAAAATATCTCTGTTCAACAAGGAGTATC
100576_CS14     AGTTAATGCTGGGCTACGTTTACCAATTACGAAAAATATCTCTGTTCAACAAGGAGTATC
503825_CS14     AGTTAATGCTGGGCTACGTTTACCAATTACGAAAAATATCTCTGTTCAACAAGGAGTATC
200023_CS14     AGTTAATGCTGGGCTACGTTTACCAATTACGAAAAATATCTCTGTTCAACAAGGAGTATC
300316_CS14     AGTTAATGCTGGGCTACGTTTACCAATTACGAAAAATATCTCTGTTCAACAAGGAGTATC
700434_CS14     AGTTAATGCTGGGCTACGTTTACCAATTACGAAAAATATCTCTGTTCAACAAGGAGTATC
602762_CS14     AGTTAATGCTGGGCTACGTTTACCAATTACGAAAAATATCTCTGTTCAACAAGGAGTATC
400599_CS14     AGTTAATGCTGGGCTACGTTTACCAATTACGAAAAATATCTCTGTTCAACAAGGAGTATC
                ************************************************************

AY283611.1      TGTTATAGATAATAAAAATTATTATGAAGGGAGTCTGAAATGGAATTCCGGCATTCTGTC
100576_CS14     TGTTATAGATAATAAAAATTATTATGAAGGGAGTCTGAAATGGAATTCCGGCATTCTGTC
503825_CS14     TGTTATAGATAATAAAAATTATTATGAAGGGAGTCTGAAATGGAATTCCGGCATTCTGTC
200023_CS14     TGTTATAGATAATAAAAATTATTATGAAGGGAGTCTGAAATGGAATTCCGGCATTCTGTC
300316_CS14     TGTTATAGATAATAAAAATTATTATGAAGGGAGTCTGAAATGGAATTCCGGCATTCTGTC
700434_CS14     TGTTATAGATAATAAAAATTATTATGAAGGGAGTCTGAAATGGAATTCCGGCATTCTGTC
602762_CS14     TGTTATAGATAATAAAAATTATTATGAAGGGAGTCTGAAATGGAATTCCGGCATTCTGTC
400599_CS14     TGTTATAGATAATAAAAATTATTATGAAGGGAGTCTGAAATGGAATTCCGGCATTCTGTC
                ************************************************************

AY283611.1      TGGTTCACTAAATAGTGAGTTCAGTTTTCTTTGGGGAGATAATGCAAAAGGTAATTATCA
100576_CS14     TGGTTCACTAAATAGTGAGTTCAGTTTTCTTTGGGGAGATAATGCAAAAGGTAATTATCA
503825_CS14     TGGTTCACTAAATAGTGAGTTCAGTTTTCTTTGGGGAGATAATGCAAAAGGTAATTATCA
200023_CS14     TGGTTCACTAAATAGTGAGTTCAGTTTTCTTTGGGGAGATAATGCAAAAGGTAATTATCA
300316_CS14     TGGTTCACTAAATAGTGAGTTCAGTTTTCTTTGGGGAGATAATGCAAAAGGTAATTATCA
700434_CS14     TGGTTCACTAAATAGTGAGTTCAGTTTTCTTTGGGGAGATAATGCAAAAGGTAATTATCA
602762_CS14     TGGTTCACTAAATAGTGAGTTCAGTTTTCTTTGGGGAGATAATGCAAAAGGTAATTATCA
400599_CS14     TGGTTCACTAAATAGTGAGTTCAGTTTTCTTTGGGGAGATAATGCAAAAGGTAATTATCA
                ************************************************************

AY283611.1      AAGTATCTCGTATACCGATGGATTTAGCTTATCATTTTATCATAATGATAAGCGGGTCGA
100576_CS14     AAGTATCTCGTATACCGATGGATTTAGCTTATCATTTTATCATAATGATAAGCGGGTCGA
503825_CS14     AAGTATCTCGTATACCGATGGATTTAGCTTATCATTTTATCATAATGATAAGCGGGTCGA
200023_CS14     AAGTATCTCGTATACCGATGGATTTAGCTTATCATTTTATCATAATGATAAGCGGGTCGA
300316_CS14     AAGTATCTCGTATACCGATGGATTTAGCTTATCATTTTATCATAATGATAAGCGGGTCGA
700434_CS14     AAGTATCTCGTATACCGATGGATTTAGCTTATCATTTTATCATAATGATAAGCGGGTCGA
602762_CS14     AAGTATCTCGTATACCGATGGATTTAGCTTATCATTTTATCATAATGATAAGCGGGTCGA
400599_CS14     AAGTATCTCGTATACCGATGGATTTAGCTTATCATTTTATCATAATGATAAGCGGGTCGA
                ************************************************************

AY283611.1      TAATTGTGGAAGAAATTACAATGCTGGTTGGAGTGGATGCTACGAATCATATTCGGCATC
100576_CS14     TAATTGTGGAAGAAATTACAATGCTGGTTGGAGTGGATGCTACGAATCATATTCGGCATC
503825_CS14     TAATTGTGGAAGAAATTACAATGCTGGTTGGAGTGGATGCTACGAATCATATTCGGCATC
200023_CS14     TAATTGTGGAAGAAATTACAATGCTGGTTGGAGTGGATGCTACGAATCATATTCGGCATC
300316_CS14     TAATTGTGGAAGAAATTACAATGCTGGTTGGAGTGGATGCTACGAATCATATTCGGCATC
700434_CS14     TAATTGTGGAAGAAATTACAATGCTGGTTGGAGTGGATGCTACGAATCATATTCGGCATC
602762_CS14     TAATTGTGGAAGAAATTACAATGCTGGTTGGAGTGGATGCTACGAATCATATTCGGCATC
400599_CS14     TAATTGTGGAAGAAATTACAATGCTGGTTGGAGTGGATGCTACGAATCATATTCGGCATC
                ************************************************************

AY283611.1      TTTAAGCATTCCTTTATTGGGATGGACAAGCACTCTGGGATATAGTGACACTTATAGTGA
100576_CS14     TTTAAGCATTCCTTTATTGGGATGGACAAGCACTCTGGGATATAGTGACACTTATAGTGA
503825_CS14     TTTAAGCATTCCTTTATTGGGATGGACAAGCACTCTGGGATATAGTGACACTTATAGTGA
200023_CS14     TTTAAGCATTCCTTTATTGGGATGGACAAGCACTCTGGGATATAGTGACACTTATAGTGA
300316_CS14     TTTAAGCATTCCTTTATTGGGATGGACAAGCACTCTGGGATATAGTGACACTTATAGTGA
700434_CS14     TTTAAGCATTCCTTTATTGGGATGGACAAGCACTCTGGGATATAGTGACACTTATAGTGA
```

```
602762_CS14      TTTAAGCATTCCTTTATTGGGATGGACAAGCACTCTGGGATATAGTGACACTTATAGTGA
400599_CS14      TTTAAGCATTCCTTTATTGGGATGGACAAGCACTCTGGGATATAGTGACACTTATAGTGA
                 ************************************************************

AY283611.1       ATCAGTTTATAAAAGTCATATTCTTTCTGAATATGGTTTTTATAATCAAAATATATATAA
100576_CS14      ATCAGTTTATAAAAGTCATATTCTTTCTGAATATGGTTTTTATAATCAAAATATATATAA
503825_CS14      ATCAGTTTATAAAAGTCATATTCTTTCTGAATATGGTTTTTATAATCAAAATATATATAA
200023_CS14      ATCAGTTTATAAAAGTCATATTCTTTCTGAATATGGTTTTTATAATCAAAATATATATAA
300316_CS14      ATCAGTTTATAAAAGTCATATTCTTTCTGAATATGGTTTTTATAATCAAAATATATATAA
700434_CS14      ATCAGTTTATAAAAGTCATATTCTTTCTGAATATGGTTTTTATAATCAAAATATATATAA
602762_CS14      ATCAGTTTATAAAAGTCATATTCTTTCTGAATATGGTTTTTATAATCAAAATATATATAA
400599_CS14      ATCAGTTTATAAAAGTCATATTCTTTCTGAATATGGTTTTTATAATCAAAATATATATAA
                 ************************************************************

AY283611.1       AGGGAGAACCCAAAAATGGCAACTGGCTTCATCCACATCTTTAAAATGGATGGATTATAA
100576_CS14      AGGGAGAACCCAAAAATGGCAACTGGCTTCATCCACATCTTTAAAATGGATGGATTATAA
503825_CS14      AGGGAGAACCCAAAAATGGCAACTGGCTTCATCCACATCTTTAAAATGGATGGATTATAA
200023_CS14      AGGGAGAACCCAAAAATGGCAACTGGCTTCATCCACATCTTTAAAATGGATGGATTATAA
300316_CS14      AGGGAGAACCCAAAAATGGCAACTGGCTTCATCCACATCTTTAAAATGGATGGATTATAA
700434_CS14      AGGGAGAACCCAAAAATGGCAACTGGCTTCATCCACATCTTTAAAATGGATGGATTATAA
602762_CS14      AGGGAGAACCCAAAAATGGCAACTGGCTTCATCCACATCTTTAAAATGGATGGATTATAA
400599_CS14      AGGGAGAACCCAAAAATGGCAACTGGCTTCATCCACATCTTTAAAATGGATGGATTATAA
                 ************************************************************

AY283611.1       TTTTATGCCAGCAATTGGAATATATAACAGTGAGCAAAGACAACTGACTGATAAAGGCGG
100576_CS14      TTTTATGCCAGCAATTGGAATATATAACAGTGAGCAAAGACAACTGACTGATAAAGGCGG
503825_CS14      TTTTATGCCAGCAATTGGAATATATAACAGTGAGCAAAGACAACTGACTGATAAAGGCGG
200023_CS14      TTTTATGCCAGCAATTGGAATATATAACAGTGAGCAAAGACAACTGACTGATAAAGGCGG
300316_CS14      TTTTATGCCAGCAATTGGAATATATAACAGTGAGCAAAGACAACTGACTGATAAAGGCGG
700434_CS14      TTTTATGCCAGCAATTGGAATATATAACAGTGAGCAAAGACAACTGACTGATAAAGGCGG
602762_CS14      TTTTATGCCAGCAATTGGAATATATAACAGTGAGCAAAGACAACTGACTGATAAAGGCGG
400599_CS14      TTTTATGCCAGCAATTGGAATATATAACAGTGAGCAAAGACAACTGACTGATAAAGGCGG
                 ************************************************************

AY283611.1       ATATATATCTGTAACTCTCACCCGAGCCAGCAGAGAAAATTCATTAAACGCAGGGTATTC
100576_CS14      ATATATATCTGTAACTCTCACCCGAGCCAGCAGAGAAAATTCATTAAACGCAGGGTATTC
503825_CS14      ATATATATCTGTAACTCTCACCCGAGCCAGCAGAGAAAATTCATTAAACGCAGGGTATTC
200023_CS14      ATATATATCTGTAACTCTCACCCGAGCCAGCAGAGAAAATTCATTAAACGCAGGGTATTC
300316_CS14      ATATATATCTGTAACTCTCACCCGAGCCAGCAGAGAAAATTCATTAAACGCAGGGTATTC
700434_CS14      ATATATATCTGTAACTCTCACCCGAGCCAGCAGAGAAAATTCATTAAACGCAGGGTATTC
602762_CS14      ATATATATCTGTAACTCTCACCCGAGCCAGCAGAGAAAATTCATTAAACGCAGGGTATTC
400599_CS14      ATATATATCTGTAACTCTCACCCGAGCCAGCAGAGAAAATTCATTAAACGCAGGGTATTC
                 ************************************************************

AY283611.1       TTACAACTATTCCAGAGGAAACTATTCTTCTGACGAATTATTTGTTGGTGGATATATGAC
100576_CS14      TTACAACTATTCCAGAGGAAACTATTCTTCTGACGAATTATTTGTTGGTGGATATATGAC
503825_CS14      TTACAACTATTCCAGAGGAAACTATTCTTCTGACGAATTATTTGTTGGTGGATATATGAC
200023_CS14      TTACAACTATTCCAGAGGAAACTATTCTTCTGACGAATTATTTGTTGGTGGATATATGAC
300316_CS14      TTACAACTATTCCAGAGGAAACTATTCTTCTGACGAATTATTTGTTGGTGGATATATGAC
700434_CS14      TTACAACTATTCCAGAGGAAACTATTCTTCTGACGAATTATTTGTTGGTGGATATATGAC
602762_CS14      TTACAACTATTCCAGAGGAAACTATTCTTCTGACGAATTATTTGTTGGTGGATATATGAC
400599_CS14      TTACAACTATTCCAGAGGAAACTATTCTTCTGACGAATTATTTGTTGGTGGATATATGAC
                 ************************************************************

AY283611.1       ATCAACAAATAATGGTGACTATCATGAGGTAGGAATGCGTTTTAATAAAAATAGACATAA
100576_CS14      ATCAACAAATAATGGTGACTATCATGAGGTAGGAATGCGTTTTAATAAAAATAGACATAA
503825_CS14      ATCAACAAATAATGGTGACTATCATGAGGTAGGAATGCGTTTTAATAAAAATAGACATAA
200023_CS14      ATCAACAAATAATGGTGACTATCATGAGGTAGGAATGCGTTTTAATAAAAATAGACATAA
300316_CS14      ATCAACAAATAATGGTGACTATCATGAGGTAGGAATGCGTTTTAATAAAAATAGACATAA
700434_CS14      ATCAACAAATAATGGTGACTATCATGAGGTAGGAATGCGTTTTAATAAAAATAGACATAA
602762_CS14      ATCAACAAATAATGGTGACTATCATGAGGTAGGAATGCGTTTTAATAAAAATAGACATAA
400599_CS14      ATCAACAAATAATGGTGACTATCATGAGGTAGGAATGCGTTTTAATAAAAATAGACATAA
                 ************************************************************

AY283611.1       TGCAGAAGGTAGACTTTCAGGTCGTATAAACAATCGATTTGGAGATTTAAATGGTTCATT
```

```
100576_CS14     TGCAGAAGGTAGACTTTCAGGTCGTATAAACAATCGATTTGGAGATTTAAATGGTTCATT
503825_CS14     TGCAGAAGGTAGACTTTCAGGTCGTATAAACAATCGATTTGGAGATTTAAATGGTTCATT
200023_CS14     TGCAGAAGGTAGACTTTCAGGTCGTATAAACAATCGATTTGGAGATTTAAATGGTTCATT
300316_CS14     TGCAGAAGGTAGACTTTCAGGTCGTATAAACAATCGATTTGGAGATTTAAATGGTTCATT
700434_CS14     TGCAGAAGGTAGACTTTCAGGTCGTATAAACAATCGATTTGGAGATTTAAATGGTTCATT
602762_CS14     TGCAGAAGGTAGACTTTCAGGTCGTATAAACAATCGATTTGGAGATTTAAATGGTTCATT
400599_CS14     TGCAGAAGGTAGACTTTCAGGTCGTATAAACAATCGATTTGGAGATTTAAATGGTTCATT
                ************************************************************

AY283611.1      CAGCATGAATAAAAACAAAAACACCAACAGCACCAATCATTCTCTCACTGGTGGTTATAA
100576_CS14     CAGCATGAATAAAAACAAAAACACCAACAGCACCAATCATTCTCTCACTGGTGGTTATAA
503825_CS14     CAGCATGAATAAAAACAAAAACACCAACAGCACCAATCATTCTCTCACTGGTGGTTATAA
200023_CS14     CAGCATGAATAAAAACAAAAACACCAACAGCACCAATCATTCTCTCACTGGTGGTTATAA
300316_CS14     CAGCATGAATAAAAACAAAAACACCAACAGCACCAATCATTCTCTCACTGGTGGTTATAA
700434_CS14     CAGCATGAATAAAAACAAAAACACCAACAGCACCAATCATTCTCTCACTGGTGGTTATAA
602762_CS14     CAGCATGAATAAAAACAAAAACACCAACAGCACCAATCATTCTCTCACTGGTGGTTATAA
400599_CS14     CAGCATGAATAAAAACAAAAACACCAACAGCACCAATCATTCTCTCACTGGTGGTTATAA
                ************************************************************

AY283611.1      TTCCTCATTTGCTCTTACAAGTGATGGATTTTACTGGGGAGGAAGTGCATCTGGTTTGAC
100576_CS14     TTCCTCATTTGCTCTTACAAGTGATGGATTTTACTGGGGAGGAAGTGCATCTGGTTTGAC
503825_CS14     TTCCTCATTTGCTCTTACAAGTGATGGATTTTACTGGGGAGGAAGTGCATCTGGTTTGAC
200023_CS14     TTCCTCATTTGCTCTTACAAGTGATGGATTTTACTGGGGAGGAAGTGCATCTGGTTTGAC
300316_CS14     TTCCTCATTTGCTCTTACAAGTGATGGATTTTACTGGGGAGGAAGTGCATCTGGTTTGAC
700434_CS14     TTCCTCATTTGCTCTTACAAGTGATGGATTTTACTGGGGAGGAAGTGCATCTGGTTTGAC
602762_CS14     TTCCTCATTTGCTCTTACAAGTGATGGATTTTACTGGGGAGGAAGTGCATCTGGTTTGAC
400599_CS14     TTCCTCATTTGCTCTTACAAGTGATGGATTTTACTGGGGAGGAAGTGCATCTGGTTTGAC
                ************************************************************

AY283611.1      AAAACTGGCTGGCGGTATTATCAAGGTTAAATCAAACGATACTAAAAAAAATCTGGTAAA
100576_CS14     AAAACTGGCTGGCGGTATTATCAAGGTTAAATCAAACGATACTAAAAAAAATCTGGTAAA
503825_CS14     AAAACTGGCTGGCGGTATTATCAAGGTTAAATCAAACGATACTAAAAAAAATCTGGTAAA
200023_CS14     AAAACTGGCTGGCGGTATTATCAAGGTTAAATCAAACGATACTAAAAAAAATCTGGTAAA
300316_CS14     AAAACTGGCTGGCGGTATTATCAAGGTTAAATCAAACGATACTAAAAAAAATCTGGTAAA
700434_CS14     AAAACTGGCTGGCGGTATTATCAAGGTTAAATCAAACGATACTAAAAAAAATCTGGTAAA
602762_CS14     AAAACTGGCTGGCGGTATTATCAAGGTTAAATCAAACGATACTAAAAAAAATCTGGTAAA
400599_CS14     AAAACTGGCTGGCGGTATTATCAAGGTTAAATCAAACGATACTAAAAAAAATCTGGTAAA
                ************************************************************

AY283611.1      AGTGACTGGGGCATTGTACGGTGATTATTCGCTAGGGAGCAACGATAATGCTTTTATTCC
100576_CS14     AGTGACTGGGGCATTGTACGGTGATTATTCGCTAGGGAGCAACGATAATGCTTTTATTCC
503825_CS14     AGTGACTGGGGCATTGTACGGTGATTATTCGCTAGGGAGCAACGATAATGCTTTTATTCC
200023_CS14     AGTGACTGGGGCATTGTACGGTGATTATTCGCTAGGGAGCAACGATAATGCTTTTATTCC
300316_CS14     AGTGACTGGGGCATTGTACGGTGATTATTCGCTAGGGAGCAACGATAATGCTTTTATTCC
700434_CS14     AGTGACTGGGGCATTGTACGGTGATTATTCGCTAGGGAGCAACGATAATGCTTTTATTCC
602762_CS14     AGTGACTGGGGCATTGTACGGTGATTATTCGCTAGGGAGCAACGATAATGCTTTTATTCC
400599_CS14     AGTGACTGGGGCATTGTACGGTGATTATTCGCTAGGGAGCAACGATAATGCTTTTATTCC
                ************************************************************

AY283611.1      TGTACCAGCATTAACTCCAGCCAGTTTAATTATTGAAGATAATAATTATGGTGACAAGAA
100576_CS14     TGTACCAGCATTAACTCCAGCCAGTTTAATTATTGAAGATAATAATTATGGTGACAAGAA
503825_CS14     TGTACCAGCATTAACTCCAGCCAGTTTAATTATTGAAGATAATAATTATGGTGACAAGAA
200023_CS14     TGTACCAGCATTAACTCCAGCCAGTTTAATTATTGAAGATAATAATTATGGTGACAAGAA
300316_CS14     TGTACCAGCATTAACTCCAGCCAGTTTAATTATTGAAGATAATAATTATGGTGACAAGAA
700434_CS14     TGTACCAGCATTAACTCCAGCCAGTTTAATTATTGAAGATAATAATTATGGTGACAAGAA
602762_CS14     TGTACCAGCATTAACTCCAGCCAGTTTAATTATTGAAGATAATAATTATGGTGACAAGAA
400599_CS14     TGTACCAGCATTAACTCCAGCCAGTTTAATTATTGAAGATAATAATTATGGTGACAAGAA
                ************************************************************

AY283611.1      TATTTCTGTACTTGCACCAACGAACAACGATATGTTTTTATTGCCGGGTAATGTTTATCC
100576_CS14     TATTTCTGTACTTGCACCAACGAACAACGATATGTTTTTATTGCCGGGTAATGTTTATCC
503825_CS14     TATTTCTGTACTTGCACCAACGAACAACGATATGTTTTTATTGCCGGGTAATGTTTATCC
200023_CS14     TATTTCTGTACTTGCACCAACGAACAACGATATGTTTTTATTGCCGGGTAATGTTTATCC
300316_CS14     TATTTCTGTACTTGCACCAACGAACAACGATATGTTTTTATTGCCGGGTAATGTTTATCC
700434_CS14     TATTTCTGTACTTGCACCAACGAACAACGATATGTTTTTATTGCCGGGTAATGTTTATCC
```

```
602762_CS14     TATTTCTGTACTTGCACCAACGAACAACGATATGTTTTTATTGCCGGGTAATGTTTATCC
400599_CS14     TATTTCTGTACTTGCACCAACGAACAACGATATGTTTTTATTGCCGGGTAATGTTTATCC
                ************************************************************

AY283611.1      TGTTGAAATTGAAACCAAAGTAAGTGTTTCTTATATTGGTAGAGGTTTTGACAAAAACGG
100576_CS14     TGTTGAAATTGAAACCAAAGTAAGTGTTTCTTATATTGGTAGAGGTTTTGACAAAAACGG
503825_CS14     TGTTGAAATTGAAACCAAAGTAAGTGTTTCTTATATTGGTAGAGGTTTTGACAAAAACGG
200023_CS14     TGTTGAAATTGAAACCAAAGTAAGTGTTTCTTATATTGGTAGAGGTTTTGACAAAAACGG
300316_CS14     TGTTGAAATTGAAACCAAAGTAAGTGTTTCTTATATTGGTAGAGGTTTTGACAAAAACGG
700434_CS14     TGTTGAAATTGAAACCAAAGTAAGTGTTTCTTATATTGGTAGAGGTTTTGACAAAAACGG
602762_CS14     TGTTGAAATTGAAACCAAAGTAAGTGTTTCTTATATTGGTAGAGGTTTTGACAAAAACGG
400599_CS14     TGTTGAAATTGAAACCAAAGTAAGTGTTTCTTATATTGGTAGAGGTTTTGACAAAAACGG
                ************************************************************

AY283611.1      CACGCCACTTTCTGGCGCACATGTTTTGAATGAACCACATGTTATCCTGGATGAGGACGG
100576_CS14     CACGCCACTTTCTGGCGCACATGTTTTGAATGAACCACATGTTATCCTGGATGAGGACGG
503825_CS14     CACGCCACTTTCTGGCGCACATGTTTTGAATGAACCACATGTTATCCTGGATGAGGACGG
200023_CS14     CACGCCACTTTCTGGCGCACATGTTTTGAATGAACCACATGTTATCCTGGATGAGGACGG
300316_CS14     CACGCCACTTTCTGGCGCACATGTTTTGAATGAACCACATGTTATCCTGGATGAGGACGG
700434_CS14     CACGCCACTTTCTGGCGCACATGTTTTGAATGAACCACATGTTATCCTGGATGAGGACGG
602762_CS14     CACGCCACTTTCTGGCGCACATGTTTTGAATGAACCACATGTTATCCTGGATGAGGACGG
400599_CS14     CACGCCACTTTCTGGCGCACATGTTTTGAATGAACCACATGTTATCCTGGATGAGGACGG
                ************************************************************

AY283611.1      AGGATTTTCGTTTGAATATACAGGTAATGAGAAAACACTTTTTTTATTAAAGGGCAGAAC
100576_CS14     AGGATTTTCGTTTGAATATACAGGTAATGAGAAAACACTTTTTTTATTAAAGGGCAGAAC
503825_CS14     AGGATTTTCGTTTGAATATACAGGTAATGAGAAAACACTTTTTTTATTAAAGGGCAGAAC
200023_CS14     AGGATTTTCGTTTGAATATACAGGTAATGAGAAAACACTTTTTTTATTAAAGGGCAGAAC
300316_CS14     AGGATTTTCGTTTGAATATACAGGTAATGAGAAAACACTTTTTTTATTAAAGGGCAGAAC
700434_CS14     AGGATTTTCGTTTGAATATACAGGTAATGAGAAAACACTTTTTTTATTAAAGGGCAGAAC
602762_CS14     AGGATTTTCGTTTGAATATACAGGTAATGAGAAAACACTTTTTTTATTAAAGGGCAGAAC
400599_CS14     AGGATTTTCGTTTGAATATACAGGTAATGAGAAAACACTTTTTTTATTAAAGGGCAGAAC
                ************************************************************

AY283611.1      TATTTATACATGTCAACTGGGGAAAAATAAAGTTCATAAAGGCATTATTTTCGTCGGAGA
100576_CS14     TATTTATACATGTCAACTGGGGAAAAATAAAGTTCATAAAGGCATTATTTTCGTCGGAGA
503825_CS14     TATTTATACATGTCAACTGGGGAAAAATAAAGTTCATAAAGGCATTATTTTCGTCGGAGA
200023_CS14     TATTTATACATGTCAACTGGGGAAAAATAAAGTTCATAAAGGCATTATTTTCGTCGGAGA
300316_CS14     TATTTATACATGTCAACTGGGGAAAAATAAAGTTCATAAAGGCATTATTTTCGTCGGAGA
700434_CS14     TATTTATACATGTCAACTGGGGAAAAATAAAGTTCATAAAGGCATTATTTTCGTCGGAGA
602762_CS14     TATTTATACATGTCAACTGGGGAAAAATAAAGTTCATAAAGGCATTATTTTCGTCGGAGA
400599_CS14     TATTTATACATGTCAACTGGGGAAAAATAAAGTTCATAAAGGCATTATTTTCGTCGGAGA
                ************************************************************

AY283611.1      TGTTATATGTGATGTTAATAGCACACGTTCCTTACCAGATGAATTTGTAAAGAACCCACG
100576_CS14     TGTTATATGTGATGTTAATAGCACACGTTCCTTACCAGATGAATTTGTAAAGAACCCACG
503825_CS14     TGTTATATGTGATGTTAATAGCACACGTTCCTTACCAGATGAATTTGTAAAGAACCCACG
200023_CS14     TGTTATATGTGATGTTAATAGCACACGTTCCTTACCAGATGAATTTGTAAAGAACCCACG
300316_CS14     TGTTATATGTGATGTTAATAGCACACGTTCCTTACCAGATGAATTTGTAAAGAACCCACG
700434_CS14     TGTTATATGTGATGTTAATAGCACACGTTCCTTACCAGATGAATTTGTAAAGAACCCACG
602762_CS14     TGTTATATGTGATGTTAATAGCACACGTTCCTTACCAGATGAATTTGTAAAGAACCCACG
400599_CS14     TGTTATATGTGATGTTAATAGCACACGTTCCTTACCAGATGAATTTGTAAAGAACCCACG
                ************************************************************

AY283611.1      TGTGCAGGATTTGCTGGCAAAGAATGATAAAGGATAAACGATGAATAAGATTTTATTTAT
100576_CS14     TGTGCAGGATTTGCTGGCAAAGAATGATAAAGGATAAACGATGAATAAGATTTTATTTAT
503825_CS14     TGTGCAGGATTTGCTGGCAAAGAATGATAAAGGATAAACGATGAATAAGATTTTATTTAT
200023_CS14     TGTGCAGGATTTGCTGGCAAAGAATGATAAAGGATAAACGATGAATAAGATTTTATTTAT
300316_CS14     TGTGCAGGATTTGCTGGCAAAGAATGATAAAGGATAAACGATGAATAAGATTTTATTTAT
700434_CS14     TGTGCAGGATTTGCTGGCAAAGAATGATAAAGGATAAACGATGAATAAGATTTTATTTAT
602762_CS14     TGTGCAGGATTTGCTGGCAAAGAATGATAAAGGATAAACGATGAATAAGATTTTATTTAT
400599_CS14     TGTGCAGGATTTGCTGGCAAAGAATGATAAAGGATAAACGATGAATAAGATTTTATTTAT
                ************************************************************

AY283611.1      TTTTACATTGTTTTTCTCTTCAGTACTTTTTACATTTGCTGTATCGGCAGATAAAATTCC
```

```
100576_CS14     TTTTACATTGTTTTTCTCTTCAGTACTTTTTACATTTGCTGTATCGGCAGATAAAATTCC
503825_CS14     TTTTACATTGTTTTTCTCTTCAGTACTTTTTACATTTGCTGTATCGGCAGATAAAATTCC
200023_CS14     TTTTACATTGTTTTTCTCTTCAGTACTTTTTACATTTGCTGTATCGGCAGATAAAATTCC
300316_CS14     TTTTACATTGTTTTTCTCTTCAGTACTTTTTACATTTGCTGTATCGGCAGATAAAATTCC
700434_CS14     TTTTACATTGTTTTTCTCTTCAGTACTTTTTACATTTGCTGTATCGGCAGATAAAATTCC
602762_CS14     TTTTACATTGTTTTTCTCTTCAGTACTTTTTACATTTGCTGTATCGGCAGATAAAATTCC
400599_CS14     TTTTACATTGTTTTTCTCTTCAGTACTTTTTACATTTGCTGTATCGGCAGATAAAATTCC
                ************************************************************

AY283611.1      CGGAGATGAGAATATAACTAATATTTTTGGCCCGCGTGACAGGAACGAATCTTCCCCCAA
100576_CS14     CGGAGATGAGAATATAACTAATATTTTTGGCCCGCGTGACAGGAACGAATCTTCCCCCAA
503825_CS14     CGGAGATGAGAATATAACTAATATTTTTGGCCCGCGTGACAGGAACGAATCTTCCCCCAA
200023_CS14     CGGAGATGAGAATATAACTAATATTTTTGGCCCGCGTGACAGGAACGAATCTTCCCCCAA
300316_CS14     CGGAGATGAGAATATAACTAATATTTTTGGCCCGCGTGACAGGAACGAATCTTCCCCCAA
700434_CS14     CGGAGATGAGAATATAACTAATATTTTTGGCCCGCGTGACAGGAACGAATCTTCCCCCAA
602762_CS14     CGGAGATGAGAATATAACTAATATTTTTGGCCCGCGTGACAGGAACGAATCTTCCCCCAA
400599_CS14     CGGAGATGAGAATATAACTAATATTTTTGGCCCGCGTGACAGGAACGAATCTTCCCCCAA
                ************************************************************

AY283611.1      ACATAATATATTAAATGACTATATTACAGCATACAGTGAAAGTCATACTCTGTATGATAG
100576_CS14     ACATAATATATTAAATGACTATATTACAGCATACAGTGAAAGTCATACTCTGTATGGTAG
503825_CS14     ACATAATATATTAAATGACTATATTACAGCATACAGTGAAAGTCATACTCTGTATGGTAG
200023_CS14     ACATAATATATTAAATGACTATATTACAGCATACAGTGAAAGTCATACTCTGTATGATAG
300316_CS14     ACATAATATATTAAATGACTATATTACAGCATACAGTGAAAGTCATACTCTGTATGATAG
700434_CS14     ACATAATATATTAAATGACTATATTACAGCATACAGTGAAAGTCATACTCTGTATGATAG
602762_CS14     ACATAATATATTAAATGACTATATTACAGCATACAGTGAAAGTCATACTCTGTATGGTAG
400599_CS14     ACATAATATATTAAATGACTATATTACAGCATACAGTGAAAGTCATACTCTGTATGATAG
                ********************************************************.***

AY283611.1      GATGATTTTTTTATGTTTGTCTTCTCAAAATACACTTAATGGAGCATGTCCAACCAGTGA
100576_CS14     GATGACTTTTTTATGTTTGTCTTCTCAAAATACACTTAATGGAGCATGTCCAACCAGTGA
503825_CS14     GATGACTTTTTTATGTTTGTCTTCTCAAAATACACTTAATGGAGCATGTCCAACCAGTGA
200023_CS14     GATGACTTTTTTATGTTTGTCTTCTCAAAATACACTTAATGGAGCATGTCCAACCAGTGA
300316_CS14     GATGATTTTTTTATGTTTGTCTTCTCAAAATACACTTAATGGAGCATGTCCAACCAGTGA
700434_CS14     GATGGTTTTTTTATGTTTGTCTTCTCAAAATACACTTAATGGAGCATGTCCAACCAGTGA
602762_CS14     GATGACTTTTTTATGTTTGTCTTCTCAAAATACACTTAATGGAGCATGTCCAACCAGTGA
400599_CS14     GATGGTTTTTTTATGTTTGTCTTCTCAAAATACACTTAATGGAGCATGTCCAACCAGTGA
                ****..******************************************************

AY283611.1      GAATCCTAGCAGTTCATCGGTCAGTGGCGAAACAAATATAACATTACAATTTACGGAAAA
100576_CS14     GAATCCTAGCAGTTCATCGGTCAGTGGCGAAACAAATATAACATTACAATTTACGGAAAA
503825_CS14     GAATCCTAGCAGTTCATCGGTCAGTGGCGAAACAAATATAACATTACAATTTACGGAAAA
200023_CS14     GAATCCTAGCAGTTCATCGGTCAGTGGCGAAACAAATATAACATTACAATTTACGGAAAA
300316_CS14     GAATCCTAGCAGTTCATCGGTCAGTGGCGAAACAAATATAACATTACAATTTACGGAAAA
700434_CS14     GAATCCTAGCAGTTCATCGGTCAGTGGCGAAACAAATATAACATTACAATTTACGGAAAA
602762_CS14     GAATCCTAGCAGTTCATCGGTCAGTGGCGAAACAAATATAACATTACAATTTACGGAAAA
400599_CS14     GAATCCTAGCAGTTCATCGGTCAGTGGCGAAACAAATATAACATTACAATTTACGGAAAA
                ************************************************************

AY283611.1      AAGAAGTTTAATTAAAAGAGAGCTACAAATTAAAGGCTATAAACGATTATTGTTCAAAGG
100576_CS14     AAGAAGTTTAATTAAAAGAGAGCTACAAATTAAAGGCTATAAACGATTATTGTTCAAAGG
503825_CS14     AAGAAGTTTAATTAAAAGAGAGCTACAAATTAAAGGCTATAAACGATTATTGTTCAAAGG
200023_CS14     AAGAAGTTTAATTAAAAGAGAGCTACAAATTAAAGGCTATAAACGATTATTGTTCAAAGG
300316_CS14     AAGAAGTTTAATTAAAAGAGAGCTACAAATTAAAGGCTATAAACGATTATTGTTCAAAGG
700434_CS14     AAGAAGTTTAATTAAAAGAGAGCTACAAATTAAAGGCTATAAACGATTATTGTTCAAAGG
602762_CS14     AAGAAGTTTAATTAAAAGAGAGCTACAAATTAAAGGCTATAAACGATTATTGTTCAAAGG
400599_CS14     AAGAAGTTTAATTAAAAGAGAGCTACAAATTAAAGGCTATAAACGATTATTGTTCAAAGG
                ************************************************************

AY283611.1      TGCTAACTGCCCATCCTACCTAACACTTAACTCAGCTCATTATACCTGCAATAGAAACTC
100576_CS14     TGTTAACTGCCCATCCTACCTAACACTTAACTCAGCTCATTATACCTGCAATAGAAACTC
503825_CS14     TGTTAACTGCCCATCCTACCTAACACTTAACTCAGCTCATTATACCTGCAATAGAAACTC
200023_CS14     TGCTAACTGCCCATCCTACCTAACACTTAACTCAGCTCATTATACCTGCAATAGAAACTC
300316_CS14     TGCTAACTGCCCATCCTACCTAACACTTAACTCAGCTCATTATACCTGCAATAGAAACTC
700434_CS14     TGATAACTGCCCATCCTACCTAACACTTAACTCAGCTCATTATACCTGCAATAGAAACTC
```

```
602762_CS14     TGTTAACTGCCCATCCTACCTAACACTTAACTCAGCTCATTATACCTGCAATAGAAACTC
400599_CS14     TGCTAACTGCCCATCCTACCTAACACTTAACTCAGCTCATTATACCTGCAATAGAAACTC
                ** *********************************************************

AY283611.1      GGCTTCAGGTGCAAGTTTATATTTATATATTCCTGCTGGCGAACTAAAAAATTTACCTTT
100576_CS14     GGCTTCAGGTGCAAGTTTATATTTATATATTCCTGCTGGCGAACTAAAAAATTTACCTTT
503825_CS14     GGCTTCAGGTGCAAGTTTATATTTATATATTCCTGCTGGCGAACTAAAAAATTTACCTTT
200023_CS14     GGCTTCAGGTGCAAGTTTATATTTATATATTCCTGCTGGCGAACTAAAAAATTTACCTTT
300316_CS14     GGCTTCAGGTGCAAGTTTATATTTATATATTCCTGCTGGCGAACTAAAAAATTTACCTTT
700434_CS14     GGCTTCAGGTGCAAGTTTATATTTATATATTCCTGCTGGCGAACTAAAAAATTTACCTTT
602762_CS14     GGCTTCAGGTGCAAGTTTATATTTATATATTCCTGCTGGCGAACTAAAAAATTTACCTTT
400599_CS14     GGCTTCAGGTGCAAGTTTATATTTATATATTCCTGCTGGCGAACTAAAAAATTTACCTTT
                ************************************************************

AY283611.1      TGGTGGTATCTGGGATGCTACTCTGAAGTTAAGAGTAAAAAGACGATATGATCAGACCTA
100576_CS14     TGGTGGTATCTGGGATGCTACTCTGAAGTTAAGAGTAAAAAGACGATATGATCAGACCTA
503825_CS14     TGGTGGTATCTGGGATGCTACTCTGAAGTTAAGAGTAAAAAGACGATATGATCAGACCTA
200023_CS14     TGGTGGTATCTGGGATGCTACTCTGAAGTTAAGAGTAAAAAGACGATATGATCAGACCTA
300316_CS14     TGGTGGTATCTGGGATGCTACTCTGAAGTTAAGAGTAAAAAGACGATATGATCAGACCTA
700434_CS14     TGGTGGTATCTGGGATGCTACTCTGAAGTTAAGAGTAAAAAGACGATATGATCAGACCTA
602762_CS14     TGGTGGTATCTGGGATGCTACTCTGAAGTTAAGAGTAAAAAGACGATATGATCAGACCTA
400599_CS14     TGGTGGTATCTGGGATGCTACTCTGAAGTTAAGAGTAAAAAGACGATATGATCAGACCTA
                ************************************************************

AY283611.1      TGGAACTTACACTATAAATATCACTGTTAAATTAACTGATAAGGGAAATATTCAGATATG
100576_CS14     TGGAACTTACACTATAAATATCACTGTTAAATTAACTGATAAGGGAAATATTCAGATATG
503825_CS14     TGGAACTTACACTATAAATATCACTGTTAAATTAACTGATAAGGGAAATATTCAGATATG
200023_CS14     TGGAACTTACACTATAAATATCACTGTTAAATTAACTGATAAGGGAAATATTCAGATATG
300316_CS14     TGGAACTTACACTATAAATATCACTGTTAAATTAACTGATAAGGGAAATATTCAGATATG
700434_CS14     TGGAACTTACACTATAAATATCACTGTTAAATTAACTGATAAGGGAAATATTCAGATATG
602762_CS14     TGGAACTTACACTATAAATATCACTGTTAAATTAACTGATAAGGGAAATATTCAGATATG
400599_CS14     TGGAACTTACACTATAAATATCACTGTTAAATTAACTGATAAGGGAAATATTCAGATATG
                ************************************************************

AY283611.1      GTTACCTCAGTTCAAAAGTGACGCTCGCGTCGATCTTAACTTGCGTCCAACTGGTGGGGG
100576_CS14     GTTACCTCAGTTCAAAAGTGACGCTCGCGTCGATCTTAACTTGCGTCCAACTGGTGGGGG
503825_CS14     GTTACCTCAGTTCAAAAGTGACGCTCGCGTCGATCTTAACTTGCGTCCAACTGGTGGGGG
200023_CS14     GTTACCTCAGTTCAAAAGTGACGCTCGCGTCGATCTTAACTTGCGTCCAACTGGTGGGGG
300316_CS14     GTTACCTCAGTTCAAAAGTGACGCTCGCGTCGATCTTAACTTGCGTCCAACTGGTGGGGG
700434_CS14     GTTACCTCAGTTCAAAAGTGACGCTCGCGTCGATCTTAACTTGCGTCCAACTGGTGGGGG
602762_CS14     GTTACCTCAGTTCAAAAGTGACGCTCGCGTCGATCTTAACTTGCGTCCAACTGGTGGGGG
400599_CS14     GTTACCTCAGTTCAAAAGTGACGCTCGCGTCGATCTTAACTTGCGTCCAACTGGTGGGGG
                ************************************************************

AY283611.1      CACATATATTGGAAGAAATTCTGTTGATATGTGCTTTTATGATGGATATAGTACTAACAG
100576_CS14     CACATATATTGGAAGAAATTCTGTTGATATGTGCTTTTATGATGGATATAGTACTAACAG
503825_CS14     CACATATATTGGAAGAAATTCTGTTGATATGTGCTTTTATGATGGATATAGTACTAACAG
200023_CS14     CACATATATTGGAAGAAATTCTGTTGATATGTGCTTTTATGATGGATATAGTACTAACAG
300316_CS14     CACATATATTGGAAGAAATTCTGTTGATATGTGCTTTTATGATGGATATAGTACTAACAG
700434_CS14     CACATATATTGGAAGAAATTCTGTTGATATGTGCTTTTATGATGGATATAGTACTAACAG
602762_CS14     CACATATATTGGAAGAAATTCTGTTGATATGTGCTTTTATGATGGATATAGTACTAACAG
400599_CS14     CACATATATTGGAAGAAATTCTGTTGATATGTGCTTTTATGATGGATATAGTACTAACAG
                ************************************************************

AY283611.1      CAGCTCTTTGGAGCTAAGATTTCAGGATAACAATCCTAAATCTGATGGGAAATTTTATCT
100576_CS14     CAGCTCTTTGGAGCTAAGATTTCAGGATAACAATCCTAAATCTGATGGGAAATTTTATCT
503825_CS14     CAGCTCTTTGGAGCTAAGATTTCAGGATAACAATCCTAAATCTGATGGGAAATTTTATCT
200023_CS14     CAGCTCTTTGGAGCTAAGATTTCAGGATAACAATCCTAAATCTGATGGGAAATTTTATCT
300316_CS14     CAGCTCTTTGGAGCTAAGATTTCAGGATAACAATCCTAAATCTGATGGGAAATTTTATCT
700434_CS14     CAGCTCTTTGGAGCTAAGATTTCAGGATAACAATCCTAAATCTGATGGGAAATTTTATCT
602762_CS14     CAGCTCTTTGGAGCTAAGATTTCAGGATAACAATCCTAAATCTGATGGGAAATTTTATCT
400599_CS14     CAGCTCTTTGGAGCTAAGATTTCAGGATAACAATCCTAAATCTGATGGGAAATTTTATCT
                ************************************************************

AY283611.1      AAGGAAAATAAATGATGACACCAAAGAAATTGCATATACTTTGTCACTTCTCTTGGCGGG
```

```
100576_CS14     AAGGAAAATAAATGATGACACCAAAGAAATTGCATATACTTTGTCACTTCTCTTGGCGGG
503825_CS14     AAGGAAAATAAATGATGACACCAAAGAAATTGCATATACTTTGTCACTTCTCTTGGCGGG
200023_CS14     AAGGAAAATAAATGATGACACCAAAGAAATTGCATATACTTTGTCACTTCTCTTGGCGGG
300316_CS14     AAGGAAAATAAATGATGACACCAAAGAAATTGCATATACTTTGTCACTTCTCTTGGCGGG
700434_CS14     AAGGAAAATAAATGATGACACCAAAGAAATTGCATATACTTTGTCACTTCTCTTGGCGGG
602762_CS14     AAGGAAAATAAATGATGACACCAAAGAAATTGCATATACTTTGTCACTTCTCTTGGCGGG
400599_CS14     AAGGAAAATAAATGATGACACCAAAGAAATTGCATATACTTTGTCACTTCTCTTGGCGGG
                ************************************************************

AY283611.1      TAAAAGTTTAACTCCAACAAATGGAACGTCATTAAATATTGCTGACGCAGCTTCTCTGGA
100576_CS14     TAAAAGTTTAACTCCAACAAATGGAACGTCATTAAATATTGCTGACGCAGCTTCTCTGGA
503825_CS14     TAAAAGTTTAACTCCAACAAATGGAACGTCATTAAATATTGCTGACGCAGCTTCTCTGGA
200023_CS14     TAAAAGTTTAACTCCAACAAATGGAACGTCATTAAATATTGCTGACGCAGCTTCTCTGGA
300316_CS14     TAAAAGTTTAACTCCAACAAATGGAACGTCATTAAATATTGCTGACGCAGCTTCTCTGGA
700434_CS14     TAAAAGTTTAACTCCAACAAATGGAACGTCATTAAATATTGCTGACGCAGCTTCTCTGGA
602762_CS14     TAAAAGTTTAACTCCAACAAATGGAACGTCATTAAATATTGCTGACGCAGCTTCTCTGGA
400599_CS14     TAAAAGTTTAACTCCAACAAATGGAACGTCATTAAATATTGCTGACGCAGCTTCTCTGGA
                ************************************************************

AY283611.1      AATAAACTGGAATAGAATTACAGCTGTCACCATGCCAGAAATCAGTGTTCCGGTGTTGTG
100576_CS14     AATAAACTGGAATAGAATTACAGCTGTCACCATGCCAGAAATCAGTGTTCCGGTGTTGTG
503825_CS14     AATAAACTGGAATAGAATTACAGCTGTCACCATGCCAGAAATCAGTGTTCCGGTGTTGTG
200023_CS14     AATAAACTGGAATAGAATTACAGCTGTCACCATGCCAGAAATCAGTGTTCCGGTGTTGTG
300316_CS14     AATAAACTGGAATAGAATTACAGCTGTCACCATGCCAGAAATCAGTGTTCCGGTGTTGTG
700434_CS14     AATAAACTGGAATAGAATTACAGCTGTCACCATGCCAGAAATCAGTGTTCCGGTGTTGTG
602762_CS14     AATAAACTGGAATAGAATTACAGCTGTCACCATGCCAGAAATCAGTGTTCCGGTGTTGTG
400599_CS14     AATAAACTGGAATAGAATTACAGCTGTCACCATGCCAGAAATCAGTGTTCCGGTGTTGTG
                ************************************************************

AY283611.1      TTGGCCTGGACGTTTGCAATTGGATGCAAAAGTGGAAAATCCCGAGGCCGGACAATATAT
100576_CS14     TTGGCCTGGACGTTTGCAATTGGATGCAAAAGTGGAAAATCCCGAGGCCGGACAATATAT
503825_CS14     TTGGCCTGGACGTTTGCAATTGGATGCAAAAGTGGAAAATCCCGAGGCCGGACAATATAT
200023_CS14     TTGGCCTGGACGTTTGCAATTGGATGCAAAAGTGGAAAATCCCGAGGCCGGACAATATAT
300316_CS14     TTGGCCTGGACGTTTGCAATTGGATGCAAAAGTGGAAAATCCCGAGGCCGGACAATATAT
700434_CS14     TTGGCCTGGACGTTTGCAATTGGATGCAAAAGTGGAAAATCCCGAGGCCGGACAATATAT
602762_CS14     TTGGCCTGGACGTTTGCAATTGGATGCAAAAGTGGAAAATCCCGAGGCCGGACAATATAT
400599_CS14     TTGGCCTGGACGTTTGCAATTGGATGCAAAAGTGGAAAATCCCGAGGCCGGACAATATAT
                ************************************************************

AY283611.1      GGGTAATATTAATATTACTTTCACACCAAGTAGTCAAACACTCTAGATAACAACAATATT
100576_CS14     GGGTAATATTAATATTACTTTCACACCAAGTAGTCAAACACTCTAGA-AACAACAATA-T
503825_CS14     GGGTAATATTAATATTACTTTCACACCAAGTAGTCAAACACTCTAGA-AACAACAATA-T
200023_CS14     GGGTAATATTAATATTACTTTCACACCAAGTAGTCAAACACTCTAGATA-----------
300316_CS14     GGGTAATATTAATATTACTTTCACACCAAGTAGTCAAACACTCTAGATAACAACAATA-T
700434_CS14     GGGTAATATTAATATTACTTTCACACCAAGTAGTCAAACACTCTAGATAACAACAATA-T
602762_CS14     GGGTAATATTAATATTACTTTCACACCAAGTAGTCAAACACTCTAGATAACAACAATA-T
400599_CS14     GGGTAATATTAATATTACTTTCACACCAAGTAGTCAAACACTCTAG-TAACAACAATA-T
                **********************************************  *

AY283611.1      GGCGCTATTGCGCGCCAATATTGTAAAGGGGTAATCTGTTTGTTAACAAAACATTTTATT
100576_CS14     GGCGCTAT----------------------------------------------------
503825_CS14     GGCGCT------------------------------------------------------
200023_CS14     ------------------------------------------------------------
300316_CS14     GGCGCT------------------------------------------------------
700434_CS14     GGCGC-------------------------------------------------------
602762_CS14     GGCGCT------------------------------------------------------
400599_CS14     GGCGCTATGC--------------------------------------------------


AY283611.1      TCAATTCAGTTTGCATCGCAATAAATCTCTACTAGAGACATTTTATACAGCATAGTATTA
100576_CS14     ------------------------------------------------------------
503825_CS14     ------------------------------------------------------------
200023_CS14     ------------------------------------------------------------
300316_CS14     ------------------------------------------------------------
700434_CS14     ------------------------------------------------------------
```

```
602762_CS14     ------------------------------------------------------------
400599_CS14     ------------------------------------------------------------


AY283611.1      TACAACACATTCAAAATAAGGTATTTT
100576_CS14     ---------------------------
503825_CS14     ---------------------------
200023_CS14     ---------------------------
300316_CS14     ---------------------------
700434_CS14     ---------------------------
602762_CS14     ---------------------------
400599_CS14     ---------------------------
```
