# Supplementary figures and images for: The role of the minor colonization factor CS14 in adherence to intestinal cell models by geographically diverse ETEC isolates
Source: mSphere. 2023 Oct 3;8(5):e00302-23. doi: 10.1128/msphere.00302-23 (PMC10597352; doi:10.1128/msphere.00302-23)

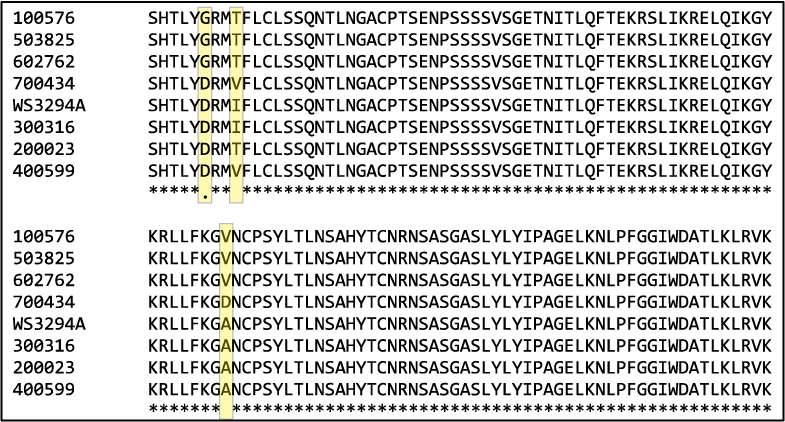

Supplement: Fig. S2 — Amino acid alignment of CS14 operons analyzed in GEMS clinical ETEC isolates. [file msphere.00302-23-s0002.tif]

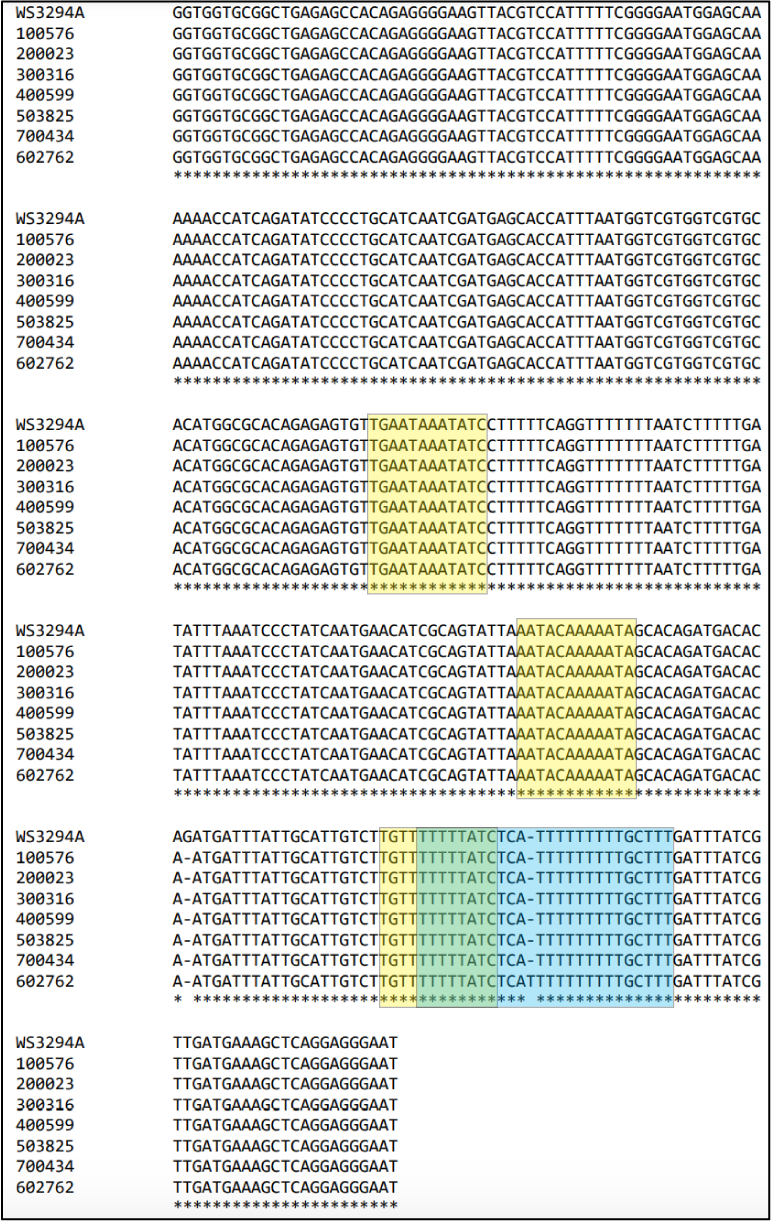

Supplement: Fig. S4 — Nucleotide alignment of CS14 promoter and upstream region from GEMS clinical ETEC isolates. [file msphere.00302-23-s0004.tif]

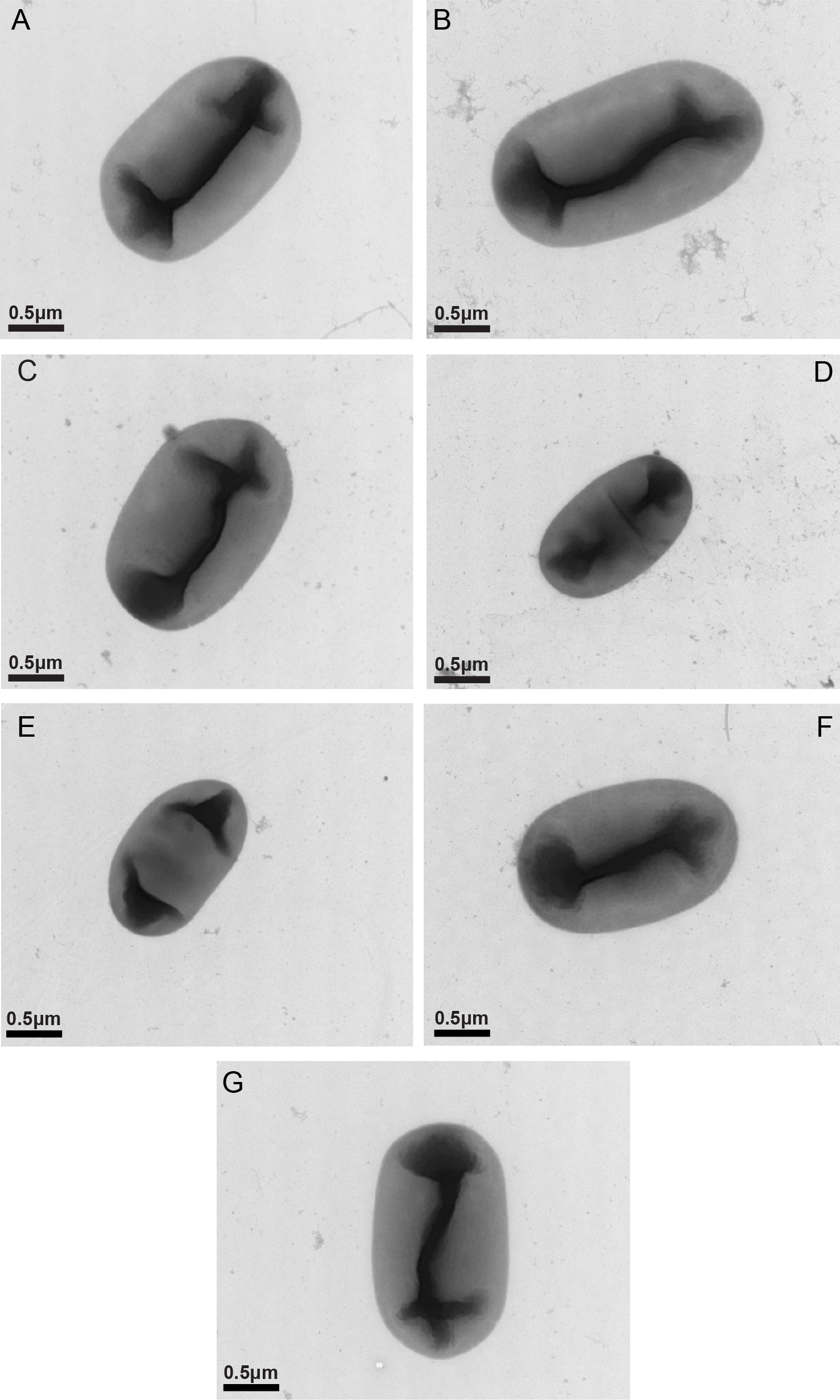

Supplement: Fig. S5 — CS14 surface expression in strains grown without iron chelation. [file msphere.00302-23-s0005.png]

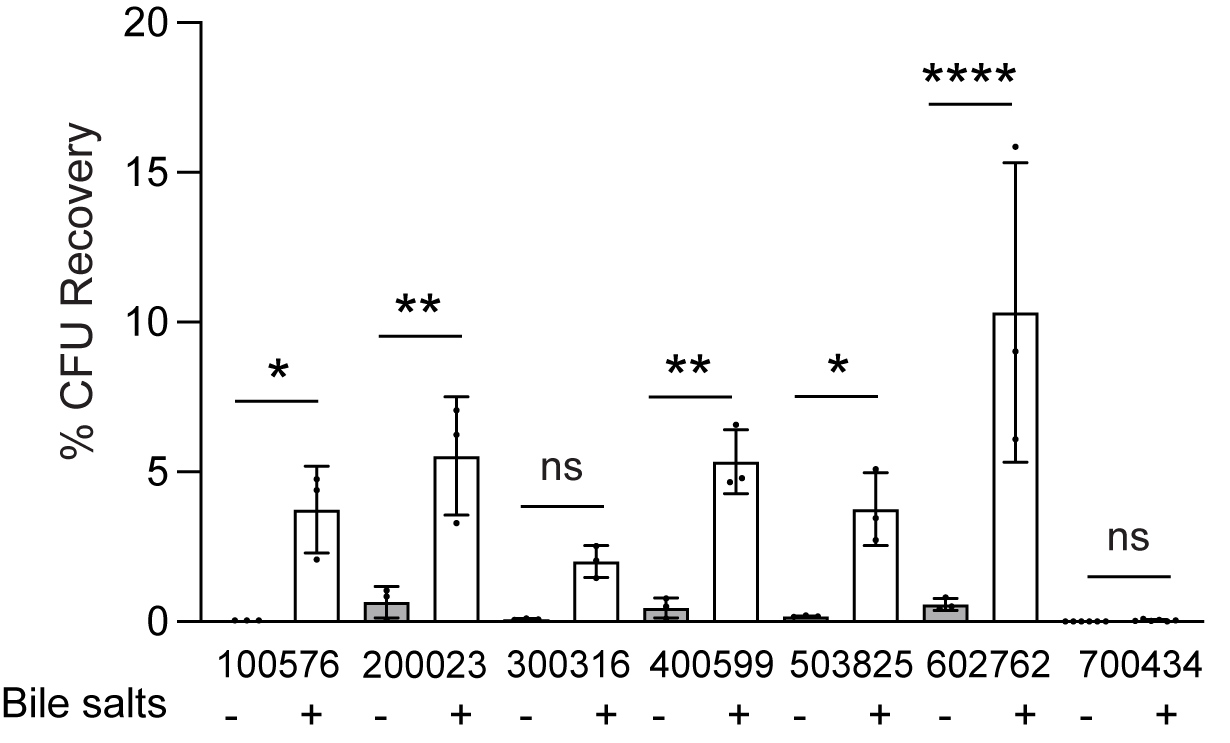

Supplement: Fig. S6 — CS14 mediates adherence of ETEC grown in bile salts to human intestinal cell monolayers. [file msphere.00302-23-s0006.tif]

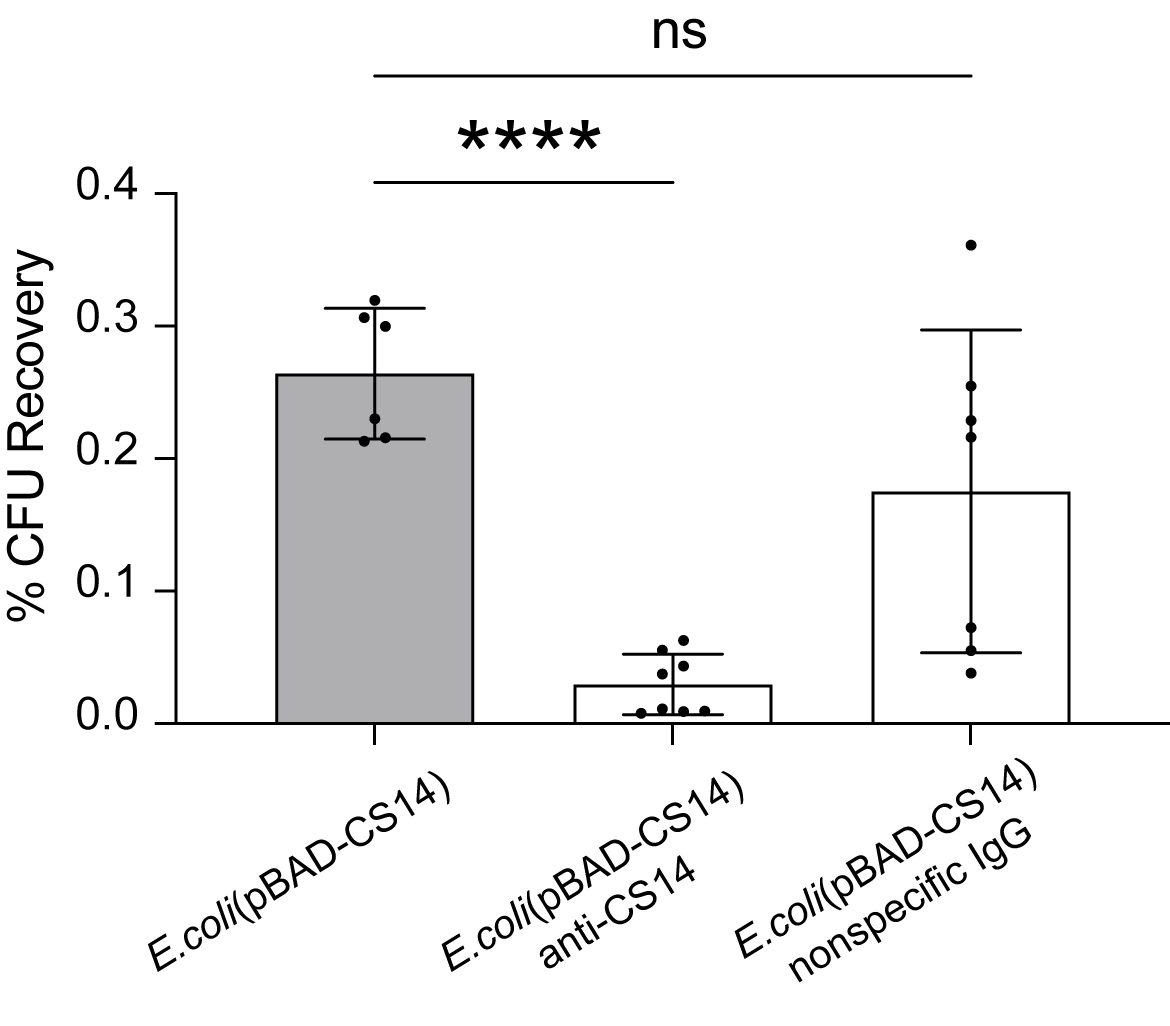

Supplement: Fig. S7 — Antibody inhibition of adherence by E. coli(pBAD-CS14) to human intestinal cell monolayers. [file msphere.00302-23-s0007.tif]
