# Supplementary material for: The role of the minor colonization factor CS14 in adherence to intestinal cell models by geographically diverse ETEC isolates
Source: mSphere. 2023 Oct 3;8(5):e00302-23. doi: 10.1128/msphere.00302-23 (PMC10597352; doi:10.1128/msphere.00302-23)
Supplement: Fig. S3 — Alignment of csuD gene in 91 ETEC strains. [file msphere.00302-23-s0003.pdf]

BLASTN 2.2.26 [Sep-21-2011]

Reference: Altschul, Stephen F., Thomas L. Madden, Alejandro A. Schaffer,
Jinghui Zhang, Zheng Zhang, Webb Miller, and David J. Lipman (1997),
"Gapped BLAST and PSI-BLAST: a new generation of protein database search
programs", Nucleic Acids Res. 25:3389-3402.

Query= WS3294A csuD
(1086 letters)

Database: all_GEMS_chile.fasta
166,761 sequences; 4,024,030,644 total letters

Searching..done

Sequences producing significant alignments:	Score (bits)	E Value
703282_98	1998	0.0
702260_124	1998	0.0
700545_121	1998	0.0
700360_123	1998	0.0
ctg7180000024743	1998	0.0
ctg7180000024742	1998	0.0
ctg7180000024709	1998	0.0
ctg7180000009184	1998	0.0
ctg7180000013219	1998	0.0
402407_85	1998	0.0
401986_90	1998	0.0
401952_76	1998	0.0
401925_134	1998	0.0
401567_75	1998	0.0
401564_73	1998	0.0
401502_69	1998	0.0
401258_95	1998	0.0
400807_73	1998	0.0
400649_89	1998	0.0
400642_95	1998	0.0
400605_67	1998	0.0
302989_108	1998	0.0
302988_106	1998	0.0
302910_76	1998	0.0
302720_108	1998	0.0
302667_93	1998	0.0
300526_109	1998	0.0
300316_87	1998	0.0
300241_78	1998	0.0

204620_144	1998	0.0
204561_103	1998	0.0
204548_67	1998	0.0
204462_81	1998	0.0
204292_113	1998	0.0
204238_75	1998	0.0
ctg7180000051711	1998	0.0
203753_113	1998	0.0
203506_134	1998	0.0
203406_87	1998	0.0
203308_119	1998	0.0
202421_168	1998	0.0
202326_123	1998	0.0
200642_142	1998	0.0
200607_110	1998	0.0
200023_101	1998	0.0
103591_111	1998	0.0
103589_144	1998	0.0
102970_98	1998	0.0
102669_99	1998	0.0
102498_95	1998	0.0
ctg7180000017726	1998	0.0
102184_111	1998	0.0
100841_101	1998	0.0
702278_124	1990	0.0
401936_124	1990	0.0
400643_106	1990	0.0
400599_77	1990	0.0
103163_145	1990	0.0
102310_126	1990	0.0
ctg7180000010531	1982	0.0
702034_97	1982	0.0
700696_97	1982	0.0
700434_99	1982	0.0
700295_82	1982	0.0
504490_77	1982	0.0
503825_89	1982	0.0
503469_123	1982	0.0
503460_112	1982	0.0
401629_61	1982	0.0
ctg7180000024819	1982	0.0
401023_79	1982	0.0
400658_88	1982	0.0
ctg7180000013184	1982	0.0
203520_77	1982	0.0
100576_98	1982	0.0
300020_190	1966	0.0
204352_232	1891	0.0
401080_129	1848	0.0
703301_181	1840	0.0

```
703163_125                                                          1820   0.0
702582_138                                                          1776   0.0
520873_p520873_84                                                   1776   0.0
510016_p510016_84                                                   1776   0.0
503458_p503458_84                                                   1776   0.0
p503046_85                                                          1776   0.0
203194_109                                                          1776   0.0
201446_95                                                           1776   0.0
602174_102                                                          1768   0.0
403776_262                                                          1556   0.0
204311_267                                                          1322   0.0
402159_112                                                           779   0.0

1_0    58    gctgtatcggcagataaaattcccggagatgagaatataactaatatttttggcccgcgt 117
147897 4574  ............................................................ 4633
141778 4868  ............................................................ 4809
136901 3439  ............................................................ 3380
134625 5443  ............................................................ 5384
99111  5916  ............................................................ 5857
99110  5971  ............................................................ 6030
99082  6317  ............................................................ 6258
97428  5379  ............................................................ 5438
94060  5247  ............................................................ 5306
91629  5971  ............................................................ 5912
87717  5969  ............................................................ 5910
87013  5970  ............................................................ 5911
86336  3342  ............................................................ 3401
82545  5971  ............................................................ 5912
82385  4876  ............................................................ 4935
82138  5970  ............................................................ 5911
80032  4485  ............................................................ 4544
75534  4876  ............................................................ 4935
73870  4889  ............................................................ 4830
73336  4627  ............................................................ 4686
72879  4511  ............................................................ 4570
65039  5970  ............................................................ 5911
64719  4627  ............................................................ 4686
64113  5146  ............................................................ 5205
62713  5969  ............................................................ 5910
62261  2541  ............................................................ 2482
58888  4627  ............................................................ 4686
58547  4624  ............................................................ 4683
57830  5169  ............................................................ 5228
51975  1820  ............................................................ 1879
51355  3342  ............................................................ 3401
51006  4898  ............................................................ 4957
50733  4511  ............................................................ 4570
48780  4439  ............................................................ 4380
48398  4511  ............................................................ 4570
44644  5509  ............................................................ 5568
```

```
43916  506   ............................................................ 565
42449  3620  ............................................................ 3561
42098  4955  ............................................................ 4896
41265  4511  ............................................................ 4570
37764  2114  ............................................................ 2055
37367  3342  ............................................................ 3401
33571  5691  ............................................................ 5632
33105  3637  ............................................................ 3578
29457  5968  ............................................................ 5909
20183  5630  ............................................................ 5571
19838  5970  ............................................................ 5911
17776  5969  ............................................................ 5910
15344  4627  ............................................................ 4686
13932  5146  ............................................................ 5205
12326  6363  ............................................................ 6304
11192  5717  ............................................................ 5658
10115  5146  ............................................................ 5205
142159 2823  ............................................................ 2882
86648  2540  ............................................................ 2481
73639  5030  ............................................................ 5089
72705  5969  ............................................................ 5910
18250  3665  ............................................................ 3606
12749  3185  ............................................................ 3126
150359 11990 ............................................................ 12049
139710 5883  ............................................................ 5942
137390 4511  ............................................................ 4570
135790 5970  ............................................................ 5911
133006 3665  ............................................................ 3606
117695 11174 ............................................................ 11233
115065 11174 ............................................................ 11233
112959 3802  ............................................................ 3861
111518 2683  ............................................................ 2742
83594  6522  ............................................................ 6581
83466  4126  ............................................................ 4067
77990  3518  ............................................................ 3577
74070  5030  ............................................................ 5089
46907  3356  ............................................................ 3415
43005  3710  ............................................................ 3651
7147   3665  ............................................................ 3606
55321  2539  ............................................................ 2480
50030  975   ............................................................ 916
78599  380   ............................c....................a.......... 439
148551 2915  ............................................................ 2974
146892 105   ............................................................ 164
144636 1921  ................................a.gc........................ 1862
120423 75260 ................................a.gc........................ 75319
119466 28454 ................................a.gc........................ 28513
111402 23529 ................................a.gc........................ 23470
110111 52982 ................................a.gc........................ 53041
40734  6386  ................................a.gc........................ 6445
```

```
35922  1919   ................................a.gc........................ 1860
124211 5681   ................................a.gc........................ 5740
102786 1                                     ............................. 29
49589  688    ............................................................ 629

1_0    118    gacaggaacgaatcttcccccaaacataatatattaaatgactatattacagcatacagt 177
147897 4634   ............................................................ 4693
141778 4808   ............................................................ 4749
136901 3379   ............................................................ 3320
134625 5383   ............................................................ 5324
99111  5856   ............................................................ 5797
99110  6031   ............................................................ 6090
99082  6257   ............................................................ 6198
97428  5439   ............................................................ 5498
94060  5307   ............................................................ 5366
91629  5911   ............................................................ 5852
87717  5909   ............................................................ 5850
87013  5910   ............................................................ 5851
86336  3402   ............................................................ 3461
82545  5911   ............................................................ 5852
82385  4936   ............................................................ 4995
82138  5910   ............................................................ 5851
80032  4545   ............................................................ 4604
75534  4936   ............................................................ 4995
73870  4829   ............................................................ 4770
73336  4687   ............................................................ 4746
72879  4571   ............................................................ 4630
65039  5910   ............................................................ 5851
64719  4687   ............................................................ 4746
64113  5206   ............................................................ 5265
62713  5909   ............................................................ 5850
62261  2481   ............................................................ 2422
58888  4687   ............................................................ 4746
58547  4684   ............................................................ 4743
57830  5229   ............................................................ 5288
51975  1880   ............................................................ 1939
51355  3402   ............................................................ 3461
51006  4958   ............................................................ 5017
50733  4571   ............................................................ 4630
48780  4379   ............................................................ 4320
48398  4571   ............................................................ 4630
44644  5569   ............................................................ 5628
43916  566    ............................................................ 625
42449  3560   ............................................................ 3501
42098  4895   ............................................................ 4836
41265  4571   ............................................................ 4630
37764  2054   ............................................................ 1995
37367  3402   ............................................................ 3461
33571  5631   ............................................................ 5572
33105  3577   ............................................................ 3518
```

```
29457  5908  ............................................................ 5849
20183  5570  ............................................................ 5511
19838  5910  ............................................................ 5851
17776  5909  ............................................................ 5850
15344  4687  ............................................................ 4746
13932  5206  ............................................................ 5265
12326  6303  ............................................................ 6244
11192  5657  ............................................................ 5598
10115  5206  ............................................................ 5265
142159 2883  ............................................................ 2942
86648  2480  ............................................................ 2421
73639  5090  ............................................................ 5149
72705  5909  ............................................................ 5850
18250  3605  ............................................................ 3546
12749  3125  ............................................................ 3066
150359 12050 ............................................................ 12109
139710 5943  ............................................................ 6002
137390 4571  ............................................................ 4630
135790 5910  ............................................................ 5851
133006 3605  ............................................................ 3546
117695 11234 ............................................................ 11293
115065 11234 ............................................................ 11293
112959 3862  ............................................................ 3921
111518 2743  ............................................................ 2802
83594  6582  ............................................................ 6641
83466  4066  ............................................................ 4007
77990  3578  ............................................................ 3637
74070  5090  ............................................................ 5149
46907  3416  ............................................................ 3475
43005  3650  ............................................................ 3591
7147   3605  ............................................................ 3546
55321  2479  ............................................................ 2420
50030  915   ............................................................ 856
78599  440   ............................................................ 499
148551 2975  ............................................................ 3034
146892 165   ............................................................ 224
144636 1861  .......................................a..c................. 1802
120423 75320 .......................................a..c................. 75379
119466 28514 .......................................a..c................. 28573
111402 23469 .......................................a..c................. 23410
110111 53042 .......................................a..c................. 53101
40734  6446  .......................................a..c................. 6505
35922  1859  .......................................a..c................. 1800
124211 5741  .......................................a..c................. 5800
102786 30    ............................................................ 89
49589  628   ............................................................ 569

1_0    178   gaaagtcatactctgtatgataggatgannnnnnnatgtttgtcttctcaaaatacactt 237
147897 4694  ............................ttttttt......................... 4753
141778 4748  ............................ttttttt......................... 4689
```

```
136901 3319  ............................ttttttt......................... 3260
134625 5323  ............................ttttttt......................... 5264
99111  5796  ............................ttttttt......................... 5737
99110  6091  ............................ttttttt......................... 6150
99082  6197  ............................ttttttt......................... 6138
97428  5499  ............................ttttttt......................... 5558
94060  5367  ............................ttttttt......................... 5426
91629  5851  ............................ttttttt......................... 5792
87717  5849  ............................ttttttt......................... 5790
87013  5850  ............................ttttttt......................... 5791
86336  3462  ............................ttttttt......................... 3521
82545  5851  ............................cttttttt........................ 5792
82385  4996  ............................ttttttt......................... 5055
82138  5850  ............................ttttttt......................... 5791
80032  4605  ............................ttttttt......................... 4664
75534  4996  ............................ttttttt......................... 5055
73870  4769  ............................ttttttt......................... 4710
73336  4747  ............................ttttttt......................... 4806
72879  4631  ............................ttttttt......................... 4690
65039  5850  ............................ttttttt......................... 5791
64719  4747  ............................ttttttt......................... 4806
64113  5266  ............................ttttttt......................... 5325
62713  5849  ............................ttttttt......................... 5790
62261  2421  ............................cttttttt........................ 2362
58888  4747  ............................ttttttt......................... 4806
58547  4744  ............................ttttttt......................... 4803
57830  5289  ............................ttttttt......................... 5348
51975  1940  ............................ttttttt......................... 1999
51355  3462  ............................ttttttt......................... 3521
51006  5018  ............................ttttttt......................... 5077
50733  4631  ............................ttttttt......................... 4690
48780  4319  ............................ttttttt......................... 4260
48398  4631  ............................ttttttt......................... 4690
44644  5629  ............................ttttttt......................... 5688
43916  626   ............................ttttttt......................... 685
42449  3500  ............................ttttttt......................... 3441
42098  4835  ............................ttttttt......................... 4776
41265  4631  ............................ttttttt......................... 4690
37764  1994  ............................ttttttt......................... 1935
37367  3462  ............................ttttttt......................... 3521
33571  5571  ............................ttttttt......................... 5512
33105  3517  ............................ttttttt......................... 3458
29457  5848  ............................cttttttt........................ 5789
20183  5510  ............................ttttttt......................... 5451
19838  5850  ............................ttttttt......................... 5791
17776  5849  ............................ttttttt......................... 5790
15344  4747  ............................ttttttt......................... 4806
13932  5266  ............................ttttttt......................... 5325
12326  6243  ............................cttttttt........................ 6184
11192  5597  ............................ttttttt......................... 5538
```

```
10115  5266   ............................ttttttt......................... 5325
142159 2943   ............................ttttttt......................... 3002
86648  2420   ...................g........ctttttt......................... 2361
73639  5150   ............................ctttttt......................... 5209
72705  5849   ...........................gttttttt......................... 5790
18250  3545   ...................g........ctttttt......................... 3486
12749  3065   ............................ttttttt......................... 3006
150359 12110  ...................g........ctttttt......................... 12169
139710 6003   ...................g........ctttttt......................... 6062
137390 4631   ...................g........ctttttt......................... 4690
135790 5850   ...........................gttttttt......................... 5791
133006 3545   ...................g........ctttttt......................... 3486
117695 11294  ...................g........ctttttt......................... 11353
115065 11294  ...................g........ctttttt......................... 11353
112959 3922   ...................g........ctttttt......................... 3981
111518 2803   ...................g........ctttttt......................... 2862
83594  6642   ...................g........ctttttt......................... 6701
83466  4006   ...................g........ctttttt......................... 3947
77990  3638   ...................g........ctttttt......................... 3697
74070  5150   ...................g........ctttttt......................... 5209
46907  3476   ...................g........ctttttt......................... 3535
43005  3590   ...................g........ctttttt......................... 3531
7147   3545   ...................g........ctttttt......................... 3486
55321  2419   ............................ttttttt......................... 2360
50030  855    ............................ttttttt......................... 796
78599  500    ............................gtttttt........................g 559
148551 3035   ...................g........ctttttt......................... 3094
146892 225    ...................g........ctttttt......................... 284
144636 1801   ............................ctttttt...............c......... 1742
120423 75380  ............................ctttttt...............c......... 75439
119466 28574  ............................ctttttt...............c......... 28633
111402 23409  ............................ctttttt...............c......... 23350
110111 53102  ............................ctttttt...............c......... 53161
40734  6506   ............................ctttttt...............c......... 6565
35922  1799   ............................ctttttt...............c......... 1740
124211 5801   ............................ctttttt...............c......... 5860
102786 90     ............................ttttttt......................... 149
49589  568    ............................ttttttt......................... 509

1_0    238    aatggagcatgtccaaccagtgagaatcctagcagttcatcggtcagtggcgaaacaaat 297
147897 4754   ............................................................ 4813
141778 4688   ............................................................ 4629
136901 3259   ............................................................ 3200
134625 5263   ............................................................ 5204
99111  5736   ............................................................ 5677
99110  6151   ............................................................ 6210
99082  6137   ............................................................ 6078
97428  5559   ............................................................ 5618
94060  5427   ............................................................ 5486
91629  5791   ............................................................ 5732
```

87717	5789	5730
87013	5790	5731
86336	3522	3581
82545	5791	5732
82385	5056	5115
82138	5790	5731
80032	4665	4724
75534	5056	5115
73870	4709	4650
73336	4807	4866
72879	4691	4750
65039	5790	5731
64719	4807	4866
64113	5326	5385
62713	5789	5730
62261	2361	2302
58888	4807	4866
58547	4804	4863
57830	5349	5408
51975	2000	2059
51355	3522	3581
51006	5078	5137
50733	4691	4750
48780	4259	4200
48398	4691	4750
44644	5689	5748
43916	686	745
42449	3440	3381
42098	4775	4716
41265	4691	4750
37764	1934	1875
37367	3522	3581
33571	5511	5452
33105	3457	3398
29457	5788	5729
20183	5450	5391
19838	5790	5731
17776	5789	5730
15344	4807	4866
13932	5326	5385
12326	6183	6124
11192	5537	5478
10115	5326	5385
142159	3003	3062
86648	2360	2301
73639	5210	5269
72705	5789	5730
18250	3485	3426
12749	3005	2946
150359	12170	12229

```
139710 6063  ............................................................ 6122
137390 4691  ............................................................ 4750
135790 5790  ............................................................ 5731
133006 3485  ............................................................ 3426
117695 11354 ............................................................ 11413
115065 11354 ............................................................ 11413
112959 3982  ............................................................ 4041
111518 2863  ............................................................ 2922
83594  6702  ............................................................ 6761
83466  3946  ............................................................ 3887
77990  3698  ............................................................ 3757
74070  5210  ............................................................ 5269
46907  3536  ............................................................ 3595
43005  3530  ............................................................ 3471
7147   3485  ............................................................ 3426
55321  2359  ............................................................ 2300
50030  795   ............................................................ 736
78599  560   ...........c....g......tgcc...g...c.g.ta.aa.tga............. 619
148551 3095  ............................................................ 3154
146892 285   ............................................................ 344
144636 1741  ...............................................c..t......... 1682
120423 75440 ...............................................c..t......... 75499
119466 28634 ...............................................c..t......... 28693
111402 23349 ...............................................c..t......... 23290
110111 53162 ...............................................c..t......... 53221
40734  6566  ...............................................c..t......... 6625
35922  1739  ...............................................c..t......... 1680
124211 5861  ...............................................c..t......... 5920
102786 150   ............................................................ 209
49589  508   ............................................................ 449

1_0    298   ataacattacaatttacggaaaaaagaagtttaattaaaagagagctacaaattaaaggc 357
147897 4814  ............................................................ 4873
141778 4628  ............................................................ 4569
136901 3199  ............................................................ 3140
134625 5203  ............................................................ 5144
99111  5676  ............................................................ 5617
99110  6211  ............................................................ 6270
99082  6077  ............................................................ 6018
97428  5619  ............................................................ 5678
94060  5487  ............................................................ 5546
91629  5731  ............................................................ 5672
87717  5729  ............................................................ 5670
87013  5730  ............................................................ 5671
86336  3582  ............................................................ 3641
82545  5731  ............................................................ 5672
82385  5116  ............................................................ 5175
82138  5730  ............................................................ 5671
80032  4725  ............................................................ 4784
75534  5116  ............................................................ 5175
```

73870	4649	4590
73336	4867	4926
72879	4751	4810
65039	5730	5671
64719	4867	4926
64113	5386	5445
62713	5729	5670
62261	2301	2242
58888	4867	4926
58547	4864	4923
57830	5409	5468
51975	2060	2119
51355	3582	3641
51006	5138	5197
50733	4751	4810
48780	4199	4140
48398	4751	4810
44644	5749	5808
43916	746	805
42449	3380	3321
42098	4715	4656
41265	4751	4810
37764	1874	1815
37367	3582	3641
33571	5451	5392
33105	3397	3338
29457	5728	5669
20183	5390	5331
19838	5730	5671
17776	5729	5670
15344	4867	4926
13932	5386	5445
12326	6123	6064
11192	5477	5418
10115	5386	5445
142159	3063	3122
86648	2300	2241
73639	5270	5329
72705	5729	5670
18250	3425	3366
12749	2945	2886
150359	12230	12289
139710	6123	6182
137390	4751	4810
135790	5730	5671
133006	3425	3366
117695	11414	11473
115065	11414	11473
112959	4042	4101
111518	2923	2982

```
83594  6762  ............................................................ 6821
83466  3886  ............................................................ 3827
77990  3758  ............................................................ 3817
74070  5270  ............................................................ 5329
46907  3596  ............................................................ 3655
43005  3470  ............................................................ 3411
7147   3425  ............................................................ 3366
55321  2299  ............................................................ 2240
50030  735   ............................................................ 676
78599  620   ............................................................ 679
148551 3155  ............................................................ 3214
146892 345   ............................................................ 404
144636 1681  ...................................a........................ 1622
120423 75500 ...................................a........................ 75559
119466 28694 ...................................a........................ 28753
111402 23289 ...................................a........................ 23230
110111 53222 ...................................a........................ 53281
40734  6626  ...................................a........................ 6685
35922  1679  ...................................a........................ 1620
124211 5921  ...................................a........................ 5980
102786 210   ............................................................ 269
49589  448   ............................................................ 389

1_0    358   tataaacgattattgttcaaaggtgctaactgcccatcctacctaacacttaactcagct 417
147897 4874  ............................................................ 4933
141778 4568  ............................................................ 4509
136901 3139  ............................................................ 3080
134625 5143  ............................................................ 5084
99111  5616  ............................................................ 5557
99110  6271  ............................................................ 6330
99082  6017  ............................................................ 5958
97428  5679  ............................................................ 5738
94060  5547  ............................................................ 5606
91629  5671  ............................................................ 5612
87717  5669  ............................................................ 5610
87013  5670  ............................................................ 5611
86336  3642  ............................................................ 3701
82545  5671  ............................................................ 5612
82385  5176  ............................................................ 5235
82138  5670  ............................................................ 5611
80032  4785  ............................................................ 4844
75534  5176  ............................................................ 5235
73870  4589  ............................................................ 4530
73336  4927  ............................................................ 4986
72879  4811  ............................................................ 4870
65039  5670  ............................................................ 5611
64719  4927  ............................................................ 4986
64113  5446  ............................................................ 5505
62713  5669  ............................................................ 5610
62261  2241  ............................................................ 2182
```

58888	4927	....	4986
58547	4924	....	4983
57830	5469	....	5528
51975	2120	....	2179
51355	3642	....	3701
51006	5198	....	5257
50733	4811	....	4870
48780	4139	....	4080
48398	4811	....	4870
44644	5809	....	5868
43916	806	....	865
42449	3320	....	3261
42098	4655	....	4596
41265	4811	....	4870
37764	1814	....	1755
37367	3642	....	3701
33571	5391	....	5332
33105	3337	....	3278
29457	5668	....	5609
20183	5330	....	5271
19838	5670	....	5611
17776	5669	....	5610
15344	4927	....	4986
13932	5446	....	5505
12326	6063	....	6004
11192	5417	....	5358
10115	5446	....	5505
142159	3123	.........................t..................................	3182
86648	2240	....	2181
73639	5330	.........................t..................................	5389
72705	5669	....	5610
18250	3365	....	3306
12749	2885	.........................t..................................	2826
150359	12290	.........................t..................................	12349
139710	6183	.........................t..................................	6242
137390	4811	.........................t..................................	4870
135790	5670	.........................a..................................	5611
133006	3365	.........................t..................................	3306
117695	11474	.........................t..................................	11533
115065	11474	.........................t..................................	11533
112959	4102	.........................t..................................	4161
111518	2983	.........................t..................................	3042
83594	6822	.........................t..................................	6881
83466	3826	.........................t..................................	3767
77990	3818	.........................t..................................	3877
74070	5330	.........................t..................................	5389
46907	3656	.........................t..................................	3715
43005	3410	.........................t..................................	3351
7147	3365	.........................t..................................	3306
55321	2239	....	2180

```
50030  675    ............................................................ 616
78599  680    ............................................................ 739
148551 3215   .........................t.................................. 3274
146892 405    .........................t.................................. 464
144636 1621   .......a.............a...t.............gg................... 1562
120423 75560  .......a.............a...t.............gg................... 75619
119466 28754  .......a.............a...t.............gg................... 28813
111402 23229  .......a.............a...t.............gg................... 23170
110111 53282  .......a.............a...t.............gg................... 53341
40734  6686   .......a.............a...t.............gg................... 6745
35922  1619   .......a.............a...t.............gg................... 1560
124211 5981   .......a.............a...t.............gg................... 6040
102786 270    ............................................................ 329
49589  388    ............................................................ 329

1_0    418    cattatacctgcaatagaaactcggcttcaggtgcaagtttatatttatatattcctgct 477
147897 4934   ............................................................ 4993
141778 4508   ............................................................ 4449
136901 3079   ............................................................ 3020
134625 5083   ............................................................ 5024
99111  5556   ............................................................ 5497
99110  6331   ............................................................ 6390
99082  5957   ............................................................ 5898
97428  5739   ............................................................ 5798
94060  5607   ............................................................ 5666
91629  5611   ............................................................ 5552
87717  5609   ............................................................ 5550
87013  5610   ............................................................ 5551
86336  3702   ............................................................ 3761
82545  5611   ............................................................ 5552
82385  5236   ............................................................ 5295
82138  5610   ............................................................ 5551
80032  4845   ............................................................ 4904
75534  5236   ............................................................ 5295
73870  4529   ............................................................ 4470
73336  4987   ............................................................ 5046
72879  4871   ............................................................ 4930
65039  5610   ............................................................ 5551
64719  4987   ............................................................ 5046
64113  5506   ............................................................ 5565
62713  5609   ............................................................ 5550
62261  2181   ............................................................ 2122
58888  4987   ............................................................ 5046
58547  4984   ............................................................ 5043
57830  5529   ............................................................ 5588
51975  2180   ............................................................ 2239
51355  3702   ............................................................ 3761
51006  5258   ............................................................ 5317
50733  4871   ............................................................ 4930
48780  4079   ............................................................ 4020
```

```
48398  4871  ............................................................ 4930
44644  5869  ............................................................ 5928
43916  866   ............................................................ 925
42449  3260  ............................................................ 3201
42098  4595  ............................................................ 4536
41265  4871  ............................................................ 4930
37764  1754  ............................................................ 1695
37367  3702  ............................................................ 3761
33571  5331  ............................................................ 5272
33105  3277  ............................................................ 3218
29457  5608  ............................................................ 5549
20183  5270  ............................................................ 5211
19838  5610  ............................................................ 5551
17776  5609  ............................................................ 5550
15344  4987  ............................................................ 5046
13932  5506  ............................................................ 5565
12326  6003  ............................................................ 5944
11192  5357  ............................................................ 5298
10115  5506  ............................................................ 5565
142159 3183  ............................................................ 3242
86648  2180  ............................................................ 2121
73639  5390  ............................................................ 5449
72705  5609  ............................................................ 5550
18250  3305  ............................................................ 3246
12749  2825  ............................................................ 2766
150359 12350 ............................................................ 12409
139710 6243  ............................................................ 6302
137390 4871  ............................................................ 4930
135790 5610  ............................................................ 5551
133006 3305  ............................................................ 3246
117695 11534 ............................................................ 11593
115065 11534 ............................................................ 11593
112959 4162  ............................................................ 4221
111518 3043  ............................................................ 3102
83594  6882  ............................................................ 6941
83466  3766  ............................................................ 3707
77990  3878  ............................................................ 3937
74070  5390  ............................................................ 5449
46907  3716  ............................................................ 3775
43005  3350  ............................................................ 3291
7147   3305  ............................................................ 3246
55321  2179  ............................................................ 2120
50030  615   ............................................................ 556
78599  740   ............................................................ 799
148551 3275  ............................................................ 3334
146892 465   ............................................................ 524
144636 1561  ....t..a...t....a....g...................................... 1502
120423 75620 ....t..a...t....a....g...................................... 75679
119466 28814 ....t..a...t....a....g...................................... 28873
111402 23169 ....t..a...t....a....g...................................... 23110
```

```
110111 53342  ....t..a...t....a....g...................................... 53401
40734  6746   ....t..a...t....a....g...................................... 6805
35922  1559   ....t..a...t....a....g...................................... 1500
124211 6041   ....t..a...t....a....g...................................... 6100
102786 330    ............................................................ 389
49589  328    ............................................................ 269

1_0    478    ggcgaactaaaaaatttaccttttggtggtatctgggatgctactctgaagttaagagta 537
147897 4994   ............................................................ 5053
141778 4448   ............................................................ 4389
136901 3019   ............................................................ 2960
134625 5023   ............................................................ 4964
99111  5496   ............................................................ 5437
99110  6391   ............................................................ 6450
99082  5897   ............................................................ 5838
97428  5799   ............................................................ 5858
94060  5667   ............................................................ 5726
91629  5551   ............................................................ 5492
87717  5549   ............................................................ 5490
87013  5550   ............................................................ 5491
86336  3762   ............................................................ 3821
82545  5551   ............................................................ 5492
82385  5296   ............................................................ 5355
82138  5550   ............................................................ 5491
80032  4905   ............................................................ 4964
75534  5296   ............................................................ 5355
73870  4469   ............................................................ 4410
73336  5047   ............................................................ 5106
72879  4931   ............................................................ 4990
65039  5550   ............................................................ 5491
64719  5047   ............................................................ 5106
64113  5566   ............................................................ 5625
62713  5549   ............................................................ 5490
62261  2121   ............................................................ 2062
58888  5047   ............................................................ 5106
58547  5044   ............................................................ 5103
57830  5589   ............................................................ 5648
51975  2240   ............................................................ 2299
51355  3762   ............................................................ 3821
51006  5318   ............................................................ 5377
50733  4931   ............................................................ 4990
48780  4019   ............................................................ 3960
48398  4931   ............................................................ 4990
44644  5929   ............................................................ 5988
43916  926    ............................................................ 985
42449  3200   ............................................................ 3141
42098  4535   ............................................................ 4476
41265  4931   ............................................................ 4990
37764  1694   ............................................................ 1635
37367  3762   ............................................................ 3821
```

```
33571  5271  ............................................................ 5212
33105  3217  ............................................................ 3158
29457  5548  ............................................................ 5489
20183  5210  ............................................................ 5151
19838  5550  ............................................................ 5491
17776  5549  ............................................................ 5490
15344  5047  ............................................................ 5106
13932  5566  ............................................................ 5625
12326  5943  ............................................................ 5884
11192  5297  ............................................................ 5238
10115  5566  ............................................................ 5625
142159 3243  ............................................................ 3302
86648  2120  ............................................................ 2061
73639  5450  ............................................................ 5509
72705  5549  ............................................................ 5490
18250  3245  ............................................................ 3186
12749  2765  ............................................................ 2706
150359 12410 ............................................................ 12469
139710 6303  ............................................................ 6362
137390 4931  ............................................................ 4990
135790 5550  ............................................................ 5491
133006 3245  ............................................................ 3186
117695 11594 ............................................................ 11653
115065 11594 ............................................................ 11653
112959 4222  ............................................................ 4281
111518 3103  ............................................................ 3162
83594  6942  ............................................................ 7001
83466  3706  ............................................................ 3647
77990  3938  ............................................................ 3997
74070  5450  ............................................................ 5509
46907  3776  ............................................................ 3835
43005  3290  ............................................................ 3231
7147   3245  ............................................................ 3186
55321  2119  ............................................................ 2060
50030  555   ............................................................ 496
78599  800   ............................................................ 859
148551 3335  ............................................................ 3394
146892 525   ............................................................ 584
144636 1501  .................g.......................................... 1442
120423 75680 .................g.......................................... 75739
119466 28874 .................g.......................................... 28933
111402 23109 .................g.......................................... 23050
110111 53402 .................g.......................................... 53461
40734  6806  .................g.......................................... 6865
35922  1499  .................g.......................................... 1440
124211 6101  .................g.......................................... 6160
102786 390   ............................................................ 449
49589  268   ............................................................ 209

1_0    538   aaaagacgatatgatcagacctatggaacttacactataaatatcactgttaaattaact 597
```

147897	5054	5113
141778	4388	4329
136901	2959	2900
134625	4963	4904
99111	5436	5377
99110	6451	6510
99082	5837	5778
97428	5859	5918
94060	5727	5786
91629	5491	5432
87717	5489	5430
87013	5490	5431
86336	3822	3881
82545	5491	5432
82385	5356	5415
82138	5490	5431
80032	4965	5024
75534	5356	5415
73870	4409	4350
73336	5107	5166
72879	4991	5050
65039	5490	5431
64719	5107	5166
64113	5626	5685
62713	5489	5430
62261	2061	2002
58888	5107	5166
58547	5104	5163
57830	5649	5708
51975	2300	2359
51355	3822	3881
51006	5378	5437
50733	4991	5050
48780	3959	3900
48398	4991	5050
44644	5989	6048
43916	986	1045
42449	3140	3081
42098	4475	4416
41265	4991	5050
37764	1634	1575
37367	3822	3881
33571	5211	5152
33105	3157	3098
29457	5488	5429
20183	5150	5091
19838	5490	5431
17776	5489	5430
15344	5107	5166
13932	5626	5685

```
12326  5883   ............................................................ 5824
11192  5237   ............................................................ 5178
10115  5626   ............................................................ 5685
142159 3303   ............................................................ 3362
86648  2060   ............................................................ 2001
73639  5510   ............................................................ 5569
72705  5489   ............................................................ 5430
18250  3185   ............................................................ 3126
12749  2705   ............................................................ 2646
150359 12470  ............................................................ 12529
139710 6363   ............................................................ 6422
137390 4991   ............................................................ 5050
135790 5490   ............................................................ 5431
133006 3185   ............................................................ 3126
117695 11654  ............................................................ 11713
115065 11654  ............................................................ 11713
112959 4282   ............................................................ 4341
111518 3163   ............................................................ 3222
83594  7002   ............................................................ 7061
83466  3646   ............................................................ 3587
77990  3998   ............................................................ 4057
74070  5510   ............................................................ 5569
46907  3836   ............................................................ 3895
43005  3230   ............................................................ 3171
7147   3185   ............................................................ 3126
55321  2059   ............................................................ 2000
50030  495    ............................................................ 436
78599  860    ............................................................ 919
148551 3395   ............................................................ 3454
146892 585    ............................................................ 644
144636 1441   ............ag.g................................a........... 1382
120423 75740  ............ag.g................................a........... 75799
119466 28934  ............ag.g................................a........... 28993
111402 23049  ............ag.g................................a........... 22990
110111 53462  ............ag.g................................a........... 53521
40734  6866   ............ag.g................................a........... 6925
35922  1439   ............ag.g................................a........... 1380
124211 6161   ............ag.g................................a........... 6220
102786 450    ............................................................ 509
49589  208    ............................................................ 149

1_0    598    gataagggaaatattcagatatggttacctcagttcaaaagtgacgctcgcgtcgatctt 657
147897 5114   ............................................................ 5173
141778 4328   ............................................................ 4269
136901 2899   ............................................................ 2840
134625 4903   ............................................................ 4844
99111  5376   ............................................................ 5317
99110  6511   ............................................................ 6570
99082  5777   ............................................................ 5718
97428  5919   ............................................................ 5978
```

94060	5787	5846
91629	5431	5372
87717	5429	5370
87013	5430	5371
86336	3882	3941
82545	5431	5372
82385	5416	5475
82138	5430	5371
80032	5025	5084
75534	5416	5475
73870	4349	4290
73336	5167	5226
72879	5051	5110
65039	5430	5371
64719	5167	5226
64113	5686	5745
62713	5429	5370
62261	2001	1942
58888	5167	5226
58547	5164	5223
57830	5709	5768
51975	2360	2419
51355	3882	3941
51006	5438	5497
50733	5051	5110
48780	3899	3840
48398	5051	5110
44644	6049	6108
43916	1046	1105
42449	3080	3021
42098	4415	4356
41265	5051	5110
37764	1574	1515
37367	3882	3941
33571	5151	5092
33105	3097	3038
29457	5428	5369
20183	5090	5031
19838	5430	5371
17776	5429	5370
15344	5167	5226
13932	5686	5745
12326	5823	5764
11192	5177	5118
10115	5686	5745
142159	3363	3422
86648	2000	1941
73639	5570	5629
72705	5429	5370
18250	3125	3066

```
12749  2645  ............................................................ 2586
150359 12530 ............................................................ 12589
139710 6423  ............................................................ 6482
137390 5051  ............................................................ 5110
135790 5430  ............................................................ 5371
133006 3125  ............................................................ 3066
117695 11714 ............................................................ 11773
115065 11714 ............................................................ 11773
112959 4342  ............................................................ 4401
111518 3223  ............................................................ 3282
83594  7062  ............................................................ 7121
83466  3586  ............................................................ 3527
77990  4058  ............................................................ 4117
74070  5570  ............................................................ 5629
46907  3896  ............................................................ 3955
43005  3170  ............................................................ 3111
7147   3125  ............................................................ 3066
55321  1999  ............................................................ 1940
50030  435   ............................................................ 376
78599  920   ............................................................ 979
148551 3455  ............................................................ 3514
146892 645   ............................................................ 704
144636 1381  ............................................................ 1322
120423 75800 ............................................................ 75859
119466 28994 ............................................................ 29053
111402 22989 ............................................................ 22930
110111 53522 ............................................................ 53581
40734  6926  ............................................................ 6985
35922  1379  ............................................................ 1320
124211 6221  ............................................................ 6280
102786 510   ............................................................ 569
49589  148   ............................................................ 89

1_0    658   aacttgcgtccaactggtgggggcacatatattggaagaaattctgttgatatgtgcttt 717
147897 5174  ............................................................ 5233
141778 4268  ............................................................ 4209
136901 2839  ............................................................ 2780
134625 4843  ............................................................ 4784
99111  5316  ............................................................ 5257
99110  6571  ............................................................ 6630
99082  5717  ............................................................ 5658
97428  5979  ............................................................ 6038
94060  5847  ............................................................ 5906
91629  5371  ............................................................ 5312
87717  5369  ............................................................ 5310
87013  5370  ............................................................ 5311
86336  3942  ............................................................ 4001
82545  5371  ............................................................ 5312
82385  5476  ............................................................ 5535
82138  5370  ............................................................ 5311
```

80032	5085	5144
75534	5476	5535
73870	4289	4230
73336	5227	5286
72879	5111	5170
65039	5370	5311
64719	5227	5286
64113	5746	5805
62713	5369	5310
62261	1941	1882
58888	5227	5286
58547	5224	5283
57830	5769	5828
51975	2420	2479
51355	3942	4001
51006	5498	5557
50733	5111	5170
48780	3839	3780
48398	5111	5170
44644	6109	6168
43916	1106	1165
42449	3020	2961
42098	4355	4296
41265	5111	5170
37764	1514	1455
37367	3942	4001
33571	5091	5032
33105	3037	2978
29457	5368	5309
20183	5030	4971
19838	5370	5311
17776	5369	5310
15344	5227	5286
13932	5746	5805
12326	5763	5704
11192	5117	5058
10115	5746	5805
142159	3423	3482
86648	1940	1881
73639	5630	5689
72705	5369	5310
18250	3065	3006
12749	2585	2526
150359	12590	12649
139710	6483	6542
137390	5111	5170
135790	5370	5311
133006	3065	3006
117695	11774	11833
115065	11774	11833

```
112959 4402  ............................................................ 4461
111518 3283  ............................................................ 3342
83594  7122  ............................................................ 7181
83466  3526  ............................................................ 3467
77990  4118  ............................................................ 4177
74070  5630  ............................................................ 5689
46907  3956  ............................................................ 4015
43005  3110  ............................................................ 3051
7147   3065  ............................................................ 3006
55321  1939  ....................t....................................... 1880
50030  375   ............................................................ 316
78599  980   ............................................................ 1039
148551 3515  ............................................................ 3574
146892 705   ............................................................ 764
144636 1321  ............................................................ 1262
120423 75860 ............................................................ 75919
119466 29054 ............................................................ 29113
111402 22929 ............................................................ 22870
110111 53582 ............................................................ 53641
40734  6986  ............................................................ 7045
35922  1319  ............................................................ 1260
124211 6281  ............................................................ 6340
102786 570   ............................................................ 629
49589  88    ............................................................ 29
89199  3206                          .................................... 3171

1_0    718   tatgatggatatagtactaacagcagctctttggagctaagatttcaggataacaatcct 777
147897 5234  ............................................................ 5293
141778 4208  ............................................................ 4149
136901 2779  ............................................................ 2720
134625 4783  ............................................................ 4724
99111  5256  ............................................................ 5197
99110  6631  ............................................................ 6690
99082  5657  ............................................................ 5598
97428  6039  ............................................................ 6098
94060  5907  ............................................................ 5966
91629  5311  ............................................................ 5252
87717  5309  ............................................................ 5250
87013  5310  ............................................................ 5251
86336  4002  ............................................................ 4061
82545  5311  ............................................................ 5252
82385  5536  ............................................................ 5595
82138  5310  ............................................................ 5251
80032  5145  ............................................................ 5204
75534  5536  ............................................................ 5595
73870  4229  ............................................................ 4170
73336  5287  ............................................................ 5346
72879  5171  ............................................................ 5230
65039  5310  ............................................................ 5251
64719  5287  ............................................................ 5346
```

64113	5806	5865
62713	5309	5250
62261	1881	1822
58888	5287	5346
58547	5284	5343
57830	5829	5888
51975	2480	2539
51355	4002	4061
51006	5558	5617
50733	5171	5230
48780	3779	3720
48398	5171	5230
44644	6169	6228
43916	1166	1225
42449	2960	2901
42098	4295	4236
41265	5171	5230
37764	1454	1395
37367	4002	4061
33571	5031	4972
33105	2977	2918
29457	5308	5249
20183	4970	4911
19838	5310	5251
17776	5309	5250
15344	5287	5346
13932	5806	5865
12326	5703	5644
11192	5057	4998
10115	5806	5865
142159	3483	3542
86648	1880	1821
73639	5690	5749
72705	5309	5250
18250	3005	2946
12749	2525	2466
150359	12650	12709
139710	6543	6602
137390	5171	5230
135790	5310	5251
133006	3005	2946
117695	11834	11893
115065	11834	11893
112959	4462	4521
111518	3343	3402
83594	7182	7241
83466	3466	3407
77990	4178	4237
74070	5690	5749
46907	4016	4075

```
43005  3050  ............................................................ 2991
7147   3005  ............................................................ 2946
55321  1879  ....................................a..............g.t...... 1820
50030  315   ............................................................ 256
78599  1040  ............................................................ 1099
148551 3575  ............................................................ 3634
146892 765   ............................................................ 824
144636 1261  ....................................a....................... 1202
120423 75920 ....................................a....................... 75979
119466 29114 ....................................a....................... 29173
111402 22869 ....................................a....................... 22810
110111 53642 ....................................a....................... 53701
40734  7046  ....................................a....................... 7105
35922  1259  ....................................a....................... 1200
124211 6341  ....................................a....................... 6400
102786 630   ............................................................ 689
49589  28    ............................                                 1
89199  3170  ............................................................ 3111

1_0    778   aaatctgatgggaaattttatctaaggaaaataaatgatgacaccaaagaaattgcatat 837
147897 5294  ............................................................ 5353
141778 4148  ............................................................ 4089
136901 2719  ............................................................ 2660
134625 4723  ............................................................ 4664
99111  5196  ............................................................ 5137
99110  6691  ............................................................ 6750
99082  5597  ............................................................ 5538
97428  6099  ............................................................ 6158
94060  5967  ............................................................ 6026
91629  5251  ............................................................ 5192
87717  5249  ............................................................ 5190
87013  5250  ............................................................ 5191
86336  4062  ............................................................ 4121
82545  5251  ............................................................ 5192
82385  5596  ............................................................ 5655
82138  5250  ............................................................ 5191
80032  5205  ............................................................ 5264
75534  5596  ............................................................ 5655
73870  4169  ............................................................ 4110
73336  5347  ............................................................ 5406
72879  5231  ............................................................ 5290
65039  5250  ............................................................ 5191
64719  5347  ............................................................ 5406
64113  5866  ............................................................ 5925
62713  5249  ............................................................ 5190
62261  1821  ............................................................ 1762
58888  5347  ............................................................ 5406
58547  5344  ............................................................ 5403
57830  5889  ............................................................ 5948
51975  2540  ............................................................ 2599
```

51355	4062	....	4121
51006	5618	....	5677
50733	5231	....	5290
48780	3719	....	3660
48398	5231	....	5290
44644	6229	....	6288
43916	1226	....	1285
42449	2900	....	2841
42098	4235	....	4176
41265	5231	....	5290
37764	1394	....	1335
37367	4062	....	4121
33571	4971	....	4912
33105	2917	....	2858
29457	5248	....	5189
20183	4910	....	4851
19838	5250	....	5191
17776	5249	....	5190
15344	5347	....	5406
13932	5866	....	5925
12326	5643	....	5584
11192	4997	....	4938
10115	5866	....	5925
142159	3543	....	3602
86648	1820	....	1761
73639	5750	....	5809
72705	5249	....	5190
18250	2945	....	2886
12749	2465	....	2406
150359	12710	....	12769
139710	6603	....	6662
137390	5231	....	5290
135790	5250	....	5191
133006	2945	....	2886
117695	11894	....	11953
115065	11894	....	11953
112959	4522	....	4581
111518	3403	....	3462
83594	7242	....	7301
83466	3406	....	3347
77990	4238	....	4297
74070	5750	....	5809
46907	4076	....	4135
43005	2990	....	2931
7147	2945	....	2886
55321	1819	....	1760
50030	255	....	196
78599	1100	....	1159
148551	3635	....	3694
146892	825	....t........c...t....	884

```
144636 1201  ............................................................ 1142
120423 75980 ............................................................ 76039
119466 29174 ............................................................ 29233
111402 22809 ............................................................ 22750
110111 53702 ............................................................ 53761
40734  7106  ............................................................ 7165
35922  1199  ............................................................ 1140
124211 6401  ....................................a....................... 6460
102786 690   ............................................................ 749
89199  3110  .........................a.....c............................ 3051

1_0    838   actttgtcacttctcttggcgggtaaaagtttaactccaacaaatggaacgtcattaaat 897
147897 5354  ............................................................ 5413
141778 4088  ............................................................ 4029
136901 2659  ............................................................ 2600
134625 4663  ............................................................ 4604
99111  5136  ............................................................ 5077
99110  6751  ............................................................ 6810
99082  5537  ............................................................ 5478
97428  6159  ............................................................ 6218
94060  6027  ............................................................ 6086
91629  5191  ............................................................ 5132
87717  5189  ............................................................ 5130
87013  5190  ............................................................ 5131
86336  4122  ............................................................ 4181
82545  5191  ............................................................ 5132
82385  5656  ............................................................ 5715
82138  5190  ............................................................ 5131
80032  5265  ............................................................ 5324
75534  5656  ............................................................ 5715
73870  4109  ............................................................ 4050
73336  5407  ............................................................ 5466
72879  5291  ............................................................ 5350
65039  5190  ............................................................ 5131
64719  5407  ............................................................ 5466
64113  5926  ............................................................ 5985
62713  5189  ............................................................ 5130
62261  1761  ............................................................ 1702
58888  5407  ............................................................ 5466
58547  5404  ............................................................ 5463
57830  5949  ............................................................ 6008
51975  2600  ............................................................ 2659
51355  4122  ............................................................ 4181
51006  5678  ............................................................ 5737
50733  5291  ............................................................ 5350
48780  3659  ............................................................ 3600
48398  5291  ............................................................ 5350
44644  6289  ............................................................ 6348
43916  1286  ............................................................ 1345
42449  2840  ............................................................ 2781
```

```
42098  4175  ............................................................ 4116
41265  5291  ............................................................ 5350
37764  1334  ............................................................ 1275
37367  4122  ............................................................ 4181
33571  4911  ............................................................ 4852
33105  2857  ............................................................ 2798
29457  5188  ............................................................ 5129
20183  4850  ............................................................ 4791
19838  5190  ............................................................ 5131
17776  5189  ............................................................ 5130
15344  5407  ............................................................ 5466
13932  5926  ............................................................ 5985
12326  5583  ............................................................ 5524
11192  4937  ............................................................ 4878
10115  5926  ............................................................ 5985
142159 3603  ............................................................ 3662
86648  1760  ............................................................ 1701
73639  5810  ............................................................ 5869
72705  5189  ............................................................ 5130
18250  2885  ............................................................ 2826
12749  2405  ............................................................ 2346
150359 12770 ............................................................ 12829
139710 6663  ............................................................ 6722
137390 5291  ............................................................ 5350
135790 5190  ............................................................ 5131
133006 2885  ............................................................ 2826
117695 11954 ............................................................ 12013
115065 11954 ............................................................ 12013
112959 4582  ............................................................ 4641
111518 3463  ............................................................ 3522
83594  7302  ............................................................ 7361
83466  3346  ............................................................ 3287
77990  4298  ............................................................ 4357
74070  5810  ............................................................ 5869
46907  4136  ............................................................ 4195
43005  2930  ............................................................ 2871
7147   2885  ............................................................ 2826
55321  1759  ............................................................ 1700
50030  195   ............................................................ 136
78599  1160  ............................................................ 1219
148551 3695  ............................................................ 3754
146892 885   ....................a.......a......a............ca.g........ 944
144636 1141  ............................................................ 1082
120423 76040 ............................................................ 76099
119466 29234 ............................................................ 29293
111402 22749 ............................................................ 22690
110111 53762 ............................................................ 53821
40734  7166  ............................................................ 7225
35922  1139  ............................................................ 1080
124211 6461  ............................................................ 6520
```

```
102786 750   .........................................................   806
89199  3050  ............................................................ 2991

1_0    898   attgctgacgcagcttctctggaaataaactggaatagaattacagctgtcaccatgcca 957
147897 5414  ............................................................ 5473
141778 4028  ............................................................ 3969
136901 2599  ............................................................ 2540
134625 4603  ............................................................ 4544
99111  5076  ............................................................ 5017
99110  6811  ............................................................ 6870
99082  5477  ............................................................ 5418
97428  6219  ............................................................ 6278
94060  6087  ............................................................ 6146
91629  5131  ............................................................ 5072
87717  5129  ............................................................ 5070
87013  5130  ............................................................ 5071
86336  4182  ............................................................ 4241
82545  5131  ............................................................ 5072
82385  5716  ............................................................ 5775
82138  5130  ............................................................ 5071
80032  5325  ............................................................ 5384
75534  5716  ............................................................ 5775
73870  4049  ............................................................ 3990
73336  5467  ............................................................ 5526
72879  5351  ............................................................ 5410
65039  5130  ............................................................ 5071
64719  5467  ............................................................ 5526
64113  5986  ............................................................ 6045
62713  5129  ............................................................ 5070
62261  1701  ............................................................ 1642
58888  5467  ............................................................ 5526
58547  5464  ............................................................ 5523
57830  6009  ............................................................ 6068
51975  2660  ............................................................ 2719
51355  4182  ............................................................ 4241
51006  5738  ............................................................ 5797
50733  5351  ............................................................ 5410
48780  3599  ............................................................ 3540
48398  5351  ............................................................ 5410
44644  6349  ............................................................ 6408
43916  1346  ............................................................ 1405
42449  2780  ............................................................ 2721
42098  4115  ............................................................ 4056
41265  5351  ............................................................ 5410
37764  1274  ............................................................ 1215
37367  4182  ............................................................ 4241
33571  4851  ............................................................ 4792
33105  2797  ............................................................ 2738
29457  5128  ............................................................ 5069
20183  4790  ............................................................ 4731
```

```
19838  5130   ............................................................ 5071
17776  5129   ............................................................ 5070
15344  5467   ............................................................ 5526
13932  5986   ............................................................ 6045
12326  5523   ............................................................ 5464
11192  4877   ............................................................ 4818
10115  5986   ............................................................ 6045
142159 3663   ............................................................ 3722
86648  1700   ............................................................ 1641
73639  5870   ............................................................ 5929
72705  5129   ............................................................ 5070
18250  2825   ............................................................ 2766
12749  2345   ............................................................ 2286
150359 12830  ............................................................ 12889
139710 6723   ............................................................ 6782
137390 5351   ............................................................ 5410
135790 5130   ............................................................ 5071
133006 2825   ............................................................ 2766
117695 12014  ............................................................ 12073
115065 12014  ............................................................ 12073
112959 4642   ............................................................ 4701
111518 3523   ............................................................ 3582
83594  7362   ............................................................ 7421
83466  3286   ............................................................ 3227
77990  4358   ............................................................ 4417
74070  5870   ............................................................ 5929
46907  4196   ............................................................ 4255
43005  2870   ............................................................ 2811
7147   2825   ............................................................ 2766
55321  1699   ............................................................ 1640
50030  135    ............................................................ 76
78599  1220   ............................................................ 1279
148551 3755   ............................................................ 3814
146892 945    ..---.a..a.t.............c.................................. 1001
144636 1081   .........................c.................................. 1022
120423 76100  .........................c.................................. 76159
119466 29294  .........................c.................................. 29353
111402 22689  .........................c.................................. 22630
110111 53822  .........................c.................................. 53881
40734  7226   .........................c.................................. 7285
35922  1079   .........................c.................................. 1020
124211 6521   .........................c.................................. 6580
89199  2990   ............................................................ 2931

1_0    958    gaaatcagtgttccggtgttgtgttggcctggacgtttgcaattggatgcaaaagtggaa 1017
147897 5474   ............................................................ 5533
141778 3968   ............................................................ 3909
136901 2539   ............................................................ 2480
134625 4543   ............................................................ 4484
99111  5016   ............................................................ 4957
```

99110	6871	....	6930
99082	5417	....	5358
97428	6279	....	6338
94060	6147	....	6206
91629	5071	....	5012
87717	5069	....	5010
87013	5070	....	5011
86336	4242	....	4301
82545	5071	....	5012
82385	5776	....	5835
82138	5070	....	5011
80032	5385	....	5444
75534	5776	....	5835
73870	3989	....	3930
73336	5527	....	5586
72879	5411	....	5470
65039	5070	....	5011
64719	5527	....	5586
64113	6046	....	6105
62713	5069	....	5010
62261	1641	....	1582
58888	5527	....	5586
58547	5524	....	5583
57830	6069	....	6128
51975	2720	....	2779
51355	4242	....	4301
51006	5798	....	5857
50733	5411	....	5470
48780	3539	....	3480
48398	5411	....	5470
44644	6409	....	6468
43916	1406	....	1465
42449	2720	....	2661
42098	4055	....	3996
41265	5411	....	5470
37764	1214	....	1155
37367	4242	....	4301
33571	4791	....	4732
33105	2737	....	2678
29457	5068	....	5009
20183	4730	....	4671
19838	5070	....	5011
17776	5069	....	5010
15344	5527	....	5586
13932	6046	....	6105
12326	5463	....	5404
11192	4817	....	4758
10115	6046	....	6105
142159	3723	....	3782
86648	1640	....	1581

```
73639  5930  ............................................................ 5989
72705  5069  ............................................................ 5010
18250  2765  ............................................................ 2706
12749  2285  ............................................................ 2226
150359 12890 ............................................................ 12949
139710 6783  ............................................................ 6842
137390 5411  ............................................................ 5470
135790 5070  ............................................................ 5011
133006 2765  ............................................................ 2706
117695 12074 ............................................................ 12133
115065 12074 ............................................................ 12133
112959 4702  ............................................................ 4761
111518 3583  ............................................................ 3642
83594  7422  ............................................................ 7481
83466  3226  ............................................................ 3167
77990  4418  ............................................................ 4477
74070  5930  ............................................................ 5989
46907  4256  ............................................................ 4315
43005  2810  ............................................................ 2751
7147   2765  ............................................................ 2706
55321  1639  ............................................................ 1580
50030  75    ............................................................ 16
78599  1280  ............................................................ 1339
148551 3815  .........................................................    3871
146892 1002  .........................................................a.. 1061
144636 1021  ............................................................ 962
120423 76160 ............................................................ 76219
119466 29354 ............................................................ 29413
111402 22629 ............................................................ 22570
110111 53882 ............................................................ 53941
40734  7286  ............................................................ 7345
35922  1019  ............................................................ 960
124211 6581  ............................................................ 6640
89199  2930  .........................................................a.. 2871

1_0    1018  aatcccgaggccggacaatatatgggtaatattaatattactttcacaccaagtagtcaa 1077
147897 5534  ............................................................ 5593
141778 3908  ............................................................ 3849
136901 2479  ............................................................ 2420
134625 4483  ............................................................ 4424
99111  4956  ............................................................ 4897
99110  6931  ............................................................ 6990
99082  5357  ............................................................ 5298
97428  6339  ............................................................ 6398
94060  6207  ............................................................ 6266
91629  5011  ............................................................ 4952
87717  5009  ............................................................ 4950
87013  5010  ............................................................ 4951
86336  4302  ............................................................ 4361
82545  5011  ............................................................ 4952
```

82385	5836	....	5895
82138	5010	....	4951
80032	5445	....	5504
75534	5836	....	5895
73870	3929	....	3870
73336	5587	....	5646
72879	5471	....	5530
65039	5010	....	4951
64719	5587	....	5646
64113	6106	....	6165
62713	5009	....	4950
62261	1581	....	1522
58888	5587	....	5646
58547	5584	....	5643
57830	6129	....	6188
51975	2780	....	2839
51355	4302	....	4361
51006	5858	....	5917
50733	5471	....	5530
48780	3479	....	3420
48398	5471	....	5530
44644	6469	....	6528
43916	1466	....	1525
42449	2660	....	2601
42098	3995	....	3936
41265	5471	....	5530
37764	1154	....	1095
37367	4302	....	4361
33571	4731	....	4672
33105	2677	....	2618
29457	5008	....	4949
20183	4670	....	4611
19838	5010	....	4951
17776	5009	....	4950
15344	5587	....	5646
13932	6106	....	6165
12326	5403	....	5344
11192	4757	....	4698
10115	6106	....	6165
142159	3783	....	3842
86648	1580	....	1521
73639	5990	....	6049
72705	5009	....	4950
18250	2705	....	2646
12749	2225	....	2166
150359	12950	....	13009
139710	6843	....	6902
137390	5471	....	5530
135790	5010	....	4951
133006	2705	....	2646

```
117695 12134 ............................................................ 12193
115065 12134 ............................................................ 12193
112959 4762  ............................................................ 4821
111518 3643  ............................................................ 3702
83594  7482  ............................................................ 7541
83466  3166  ............................................................ 3107
77990  4478  ............................................................ 4537
74070  5990  ............................................................ 6049
46907  4316  ............................................................ 4375
43005  2750  ............................................................ 2691
7147   2705  ............................................................ 2646
55321  1579  ............................................................ 1520
50030  15    ...............                                              1
78599  1340  ............................................................ 1399
146892 1062  ...........t..............g........a........................ 1121
144636 961   ...........t.......................g....................... 902
120423 76220 ...........t.......................g....................... 76279
119466 29414 ...........t.......................g....................... 29473
111402 22569 ...........t.......................g....................... 22510
110111 53942 ...........t.......................g....................... 54001
40734  7346  ...........t.......................g....................... 7405
35922  959   ...........t.......................g....................... 900
124211 6641  ...........t.......................g....................... 6700
89199  2870  ............................................................ 2811

1_0    1078  acactctag 1086
147897 5594  ......... 5602
141778 3848  ......... 3840
136901 2419  ......... 2411
134625 4423  ......... 4415
99111  4896  ......... 4888
99110  6991  ......... 6999
99082  5297  ......... 5289
97428  6399  ......... 6407
94060  6267  ......... 6275
91629  4951  ......... 4943
87717  4949  ......... 4941
87013  4950  ......... 4942
86336  4362  ......... 4370
82545  4951  ......... 4943
82385  5896  ......... 5904
82138  4950  ......... 4942
80032  5505  ......... 5513
75534  5896  ......... 5904
73870  3869  ......... 3861
73336  5647  ......... 5655
72879  5531  ......... 5539
65039  4950  ......... 4942
64719  5647  ......... 5655
64113  6166  ......... 6174
```

62713	4949	....	4941
62261	1521	....	1513
58888	5647	....	5655
58547	5644	....	5652
57830	6189	....	6197
51975	2840	....	2848
51355	4362	....	4370
51006	5918	....	5926
50733	5531	....	5539
48780	3419	....	3411
48398	5531	....	5539
44644	6529	....	6537
43916	1526	....	1534
42449	2600	....	2592
42098	3935	....	3927
41265	5531	....	5539
37764	1094	....	1086
37367	4362	....	4370
33571	4671	....	4663
33105	2617	....	2609
29457	4948	....	4940
20183	4610	....	4602
19838	4950	....	4942
17776	4949	....	4941
15344	5647	....	5655
13932	6166	....	6174
12326	5343	....	5335
11192	4697	....	4689
10115	6166	....	6174
142159	3843	....	3851
86648	1520	....	1512
73639	6050	....	6058
72705	4949	....	4941
18250	2645	....	2637
12749	2165	....	2157
150359	13010	....	13018
139710	6903	....	6911
137390	5531	....	5539
135790	4950	....	4942
133006	2645	....	2637
117695	12194	....	12202
115065	12194	....	12202
112959	4822	....	4830
111518	3703	....	3711
83594	7542	....	7550
83466	3106	....	3098
77990	4538	....	4546
74070	6050	....	6058
46907	4376	....	4384
43005	2690	....	2682

```
7147   2645  ......... 2637
55321  1519  ......... 1511
78599  1400  ......... 1408
146892 1122  ......... 1130
144636 901   ......... 893
120423 76280 ......... 76288
119466 29474 ......... 29482
111402 22509 ......... 22501
110111 54002 ......... 54010
40734  7406  ......... 7414
35922  899   ......... 891
124211 6701  ......... 6709
89199  2810  ......... 2802
  Database: all_GEMS_chile.fasta
    Posted date:  Apr 8, 2023  6:51 AM
  Number of letters in database: 4,024,030,644
  Number of sequences in database:  166,761

Lambda      K        H
    1.37    0.711     1.31

Gapped
Lambda      K        H
    1.37    0.711     1.31


Matrix: blastn matrix:1 -3
Gap Penalties: Existence: 5, Extension: 2
Number of Sequences: 166761
Number of Hits to DB: 37,092,669
Number of extensions: 11950
Number of successful extensions: 11950
Number of sequences better than 1.0e-200: 91
Number of HSP's gapped: 11863
Number of HSP's successfully gapped: 91
Length of query: 1086
Length of database: 4,024,030,644
Length adjustment: 21
Effective length of query: 1065
Effective length of database: 4,020,528,663
Effective search space: 4281863026095
Effective search space used: 4281863026095
X1: 11 (21.8 bits)
X2: 15 (29.7 bits)
X3: 50 (99.1 bits)
S1: 15 (30.2 bits)
S2: 357 (708.2 bits)
```
