# Supplementary material for: The role of the minor colonization factor CS14 in adherence to intestinal cell models by geographically diverse ETEC isolates
Source: mSphere. 2023 Oct 3;8(5):e00302-23. doi: 10.1128/msphere.00302-23 (PMC10597352; doi:10.1128/msphere.00302-23)
Supplement: Legends — Supplemental figure legends. [file msphere.00302-23-s0008.docx]

**Supplemental Material – Supplemental Figure Legends**

**Figure S1. Multiple sequence alignment of CS14 operon from CS14+ GEMS clinical ETEC isolates studied determined using clustal format alignment tool MAFFT.**

**Figure S2. Multiple amino acid sequence alignment of CS14 operon from GEMS clinical ETEC isolates determined using clustal format alignment tool MAFFT.** Residues highlighted in yellow represent the amino acid changes as a result of the nucleotide differences at the “hot spot” locations, compared to the reference strain WS3294A.

**Figure S3. Multiple sequence alignment of *csuD* gene of 91 total ETEC isolates, including the seven CS14+ GEMS clinical ETEC isolates, determined using gapped BLASTn and PSI-BLAST.**

**Figure S4. Multiple sequence alignment of CS14 promoter and upstream region (321bp) from GEMS clinical ETEC isolates studied using clustal format alignment tool MAFFT.** Nucleotides highlighted in yellow represent the three Rns binding sites and nucleotides highlighted in blue represent the single IscR putative binding site. Nucleotides highlighted in green represent the overlapping binding sites. The second Rns binding site is on the noncoding strand.

**Figure S5. CS14 surface expression was not observed in GEMS clinical ETEC isolates grown on CFA agar without DFOM.** ETEC strains grown on CFA agar were stained with uranyl acetate and visualized using TEM. Strains are as follows: 100576 (A); 200023 (B); 300316 (C); 400599 (D); 503825 (E); 6026762 (F); and 700434 (G). Scale bar size indicated in μM.

**Figure S6.** **CS14 mediates adherence of ETEC grown in bile salts to human intestinal cell monolayers.** Human HT-29 cell monolayers were infected with wildtype ETEC strains grown on CFA agar with (+) or without (-) 0.15% bile salts for 2 hrs. Monolayers were washed and lysed to quantify adherent bacteria expressed as % of initial inoculum (CFU recovery). Data presented are pooled from four independent experiments. Each dot represents data collected from an individual monolayer. Error bars indicate standard deviations from the means. The asterisks above the bars indicate statistically significant differences determined using Welch ANOVA with Sidak multiple-comparison test. *, p<0.05; **, p<0.01; ****, p<0.0001.

**Figure S7. Antibodies to CS14, but not nonspecific antibodies, inhibit adherence by control strain *E.coli*(pBAD-CS14) to human intestinal cell monolayers.** Human HT-29 cell monolayers were infected with *E.coli*(pBAD-CS14) strain for 2 hrs with or without pre-incubation with rabbit anti-CS14 antibodies or rabbit nonspecific antibodies. Monolayers were washed and lysed to quantify adherent bacteria expressed as % of initial inoculum (CFU recovery). Data presented are pooled from two independent experiments. Each dot represents data collected from an individual monolayer. Error bars indicate standard deviations from the means. The asterisks above the bars indicate statistically significant differences determined using Welch ANOVA with Sidak multiple-comparison test. ****, p<0.0001.
