# Supplementary material for: The role of the minor colonization factor CS14 in adherence to intestinal cell models by geographically diverse ETEC isolates
Source: mSphere. 2023 Oct 3;8(5):e00302-23. doi: 10.1128/msphere.00302-23 (PMC10597352; doi:10.1128/msphere.00302-23)
Supplement: Supplemental Tables — Tables S1 and S2. [file msphere.00302-23-s0009.docx]

**Supplemental Material – Supplemental Tables**

**Table S1. Percent nucleotide identity of the *csuD* gene from the CS14 operon of GEMS clinical ETEC isolates determined by multiple sequence alignment using clustal format alignment tool MAFFT.**

|  | **WS3294A** | **300316** | **200023** | **400599** | **700434** | **100576** | **503825** | **602762** |
| --- | --- | --- | --- | --- | --- | --- | --- | --- |
| **WS3294A** | 100 | 100 | 99.91 | 99.91 | 99.82 | 99.72 | 99.72 | 99.72 |
| **300316** | 100 | 100 | 99.91 | 99.91 | 99.82 | 99.72 | 99.72 | 99.72 |
| **200023** | 99.91 | 99.91 | 100 | 99.82 | 99.72 | 99.82 | 99.82 | 99.82 |
| **400599** | 99.91 | 99.91 | 99.82 | 100 | 99.91 | 99.63 | 99.63 | 99.63 |
| **700434** | 99.82 | 99.82 | 99.72 | 99.91 | 100 | 99.63 | 99.63 | 99.63 |
| **100576** | 99.72 | 99.72 | 99.82 | 99.63 | 99.63 | 100 | 100 | 100 |
| **503825** | 99.72 | 99.72 | 99.82 | 99.63 | 99.63 | 100 | 100 | 100 |
| **602762** | 99.72 | 99.72 | 99.82 | 99.63 | 99.63 | 100 | 100 | 100 |

Colors indicate lowest level of identity (white) to the highest level of identity (dark blue).

**Table S2. Specific shared and unique genes in CS14+ GEMS clinical ETEC isolates using LSBSR analysis.**

|  | ***csuA1*** | ***csuA2*** | ***csuB*** | ***csuC*** | ***csuD*** | ***sth*** | ***etpA*** | ***etpB*** | ***etpC*** | ***fimH*** | ***yghJ*** | ***eatA*** | ***hns*** | ***iscR*** | ***rns*** |
| --- | --- | --- | --- | --- | --- | --- | --- | --- | --- | --- | --- | --- | --- | --- | --- |
| **100576** | + | + | + | + | + | + | + | + | + | - | + | + | + | + | + |
| **200023** | + | + | + | + | + | + | + | + | + | - | + | - | + | + | + |
| **300316** | + | + | + | + | + | + | + | + | + | - | + | - | + | + | + |
| **400599** | + | + | + | + | + | + | + | + | + | - | + | - | + | + | + |
| **503825** | + | + | + | + | + | + | + | + | + | + | + | + | + | + | + |
| **602762** | + | + | + | + | + | + | + | + | + | + | + | + | + | + | + |
| **700434** | + | + | + | + | + | + | + | + | + | + | + | - | + | + | + |

+ indicates virulence gene was present with a minimum BLASTn BSR > 0.80 and - is equivalent to any genes <0.40. The genes *csuA1/csuA2*, *sth* and *etpA* were confirmed by PCR.
